# Combined Inductive and Dispersion Effects Enhance Bioorthogonal Reactivity of Tetrazines Toward Isonitriles

**DOI:** 10.1002/anie.202501235

**Published:** 2025-08-10

**Authors:** Suprakash Biswas, Andreas Löffler, Pushkar Bansal, Randall T. Peterson, Dennis Svatunek, Raphael M. Franzini

**Affiliations:** ^1^ Department of Medicinal Chemistry University of Utah 30 S 2000 E Salt Lake City UT 84112 USA; ^2^ Institute of Applied Synthetic Chemistry TU Wien, Getreidemarkt 9 Vienna 1060 Austria; ^3^ Department of Pharmacology and Toxicology University of Utah 30 S 2000 E Salt Lake City UT 84112 USA; ^4^ Huntsman Cancer Institute 2000 Circle of Hope Salt Lake City UT 84112 USA

**Keywords:** Bioorthogonal chemistry, Chemoselectivity, Cycloaddition, Density functional calculations, Isonitriles

## Abstract

Inverse‐electron demand cycloaddition reactions of tetrazines are widely used in bioorthogonal chemistry, but the opposing effects of substituents on reactivity and stability make optimizing tetrazines for chemical biology applications challenging. Building on the discovery that bulky substituents can unexpectedly enhance both the stability of tetrazines and their reactivity toward isonitriles, we hypothesized that substituents that are both bulky and electron‐withdrawing could yield tetrazines with desirable properties. We synthesized a series of tetrazines designed to explore these kinetic properties. A novel computational method quantifying intermolecular atomic contributions to dispersion forces supported the analysis substituent effects on tetrazine reactivity. Study results indicate that tetrazine reactivity is governed by an interplay of frontier‐orbital levels, dispersion forces, and conformational preferences. These insights were apparent in the form of a “bromo effect,” where bromine atoms enhanced tetrazine reactivity by influencing frontier‐orbital levels and increasing dispersion forces. Notably, 3,6‐bis(2‐bromopropan‐2‐yl)‐1,2,4,5‐tetrazine exhibited a ∼80‐fold increase in reactivity to isonitriles compared to dimethyltetrazine with high orthogonality to other dienophiles. The 2‐bromoprop‐2‐yl group could also be incorporated into asymmetric tetrazines for tuning reactivity properties. In addition to introducing novel tetrazines for bioorthogonal chemistry, this work provides valuable insights into the role of dispersion forces in the transition states of cycloaddition reactions.

## Graphical abstract

Bulky, electron‐withdrawing substituents can significantly enhance the reactivity and stability of tetrazines toward isonitriles, addressing a key challenge in bioorthogonal chemistry. This study explores the role of frontier‐orbital interactions, dispersion forces, and molecular conformation in optimizing tetrazine performance, leading to highly reactive and orthogonal bioorthogonal reagents.

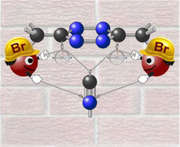

The groundbreaking idea of elucidating biological processes through chemistry has spurred a collective effort to develop reactions compatible with living organisms.^[^ ^1^, ^2^, ^3^ ^]^ To meet the needs of bioconjugation, imaging, and drug delivery, considerable efforts have been dedicated for uncovering bioorthogonal reagent pairs that offer high reactivity while preserving orthogonality to biomolecules and ideally to each other.^[^ ^4^, ^5^, ^6^ ^]^ Cycloaddition reactions, particularly those involving 1,2,4,5‐tetrazines (Tz), have proven to be prolific for in vivo chemistry.^[^ ^7^, ^8^ ^]^ Tz readily engage in inverse‐electron demand Diels–Alder (IEDDA) reactions with alkenes,^[^ ^9^, ^10^ ^]^ alkynes,^[^ ^11^ ^]^ enamines,^[^ ^12^ ^]^ and isonitriles.^[^ ^13^, ^14^ ^]^

Numerous researchers have advanced IEDDA reactions by fine‐tuning the structural strain and orbital geometry of alkenes/alkynes^[^ ^1^, ^3^, ^15^ ^]^ and identifying alternative dienes.^[^ ^16^, ^17^, ^18^, ^19^ ^]^ However, efforts to enhance the reactivity of Tz are complicated by a reactivity/stability tradeoff.^[^ ^20^, ^21^ ^]^ Modifications that increase Tz reactivity, such as lowering LUMO levels and minimizing substituent bulkiness, often make them unstable (Figure 1a). Despite recent advances based on orbital repulsions,^[^ ^22^, ^23^ ^]^ second‐orbital interactions,^[^ ^24^ ^]^ and chelation,^[^ ^25^, ^26^ ^]^ the Tz used nowadays are largely the same as when this bioorthogonal chemistry was first established.^[^ ^9^, ^10^ ^]^

**Figure 1:**
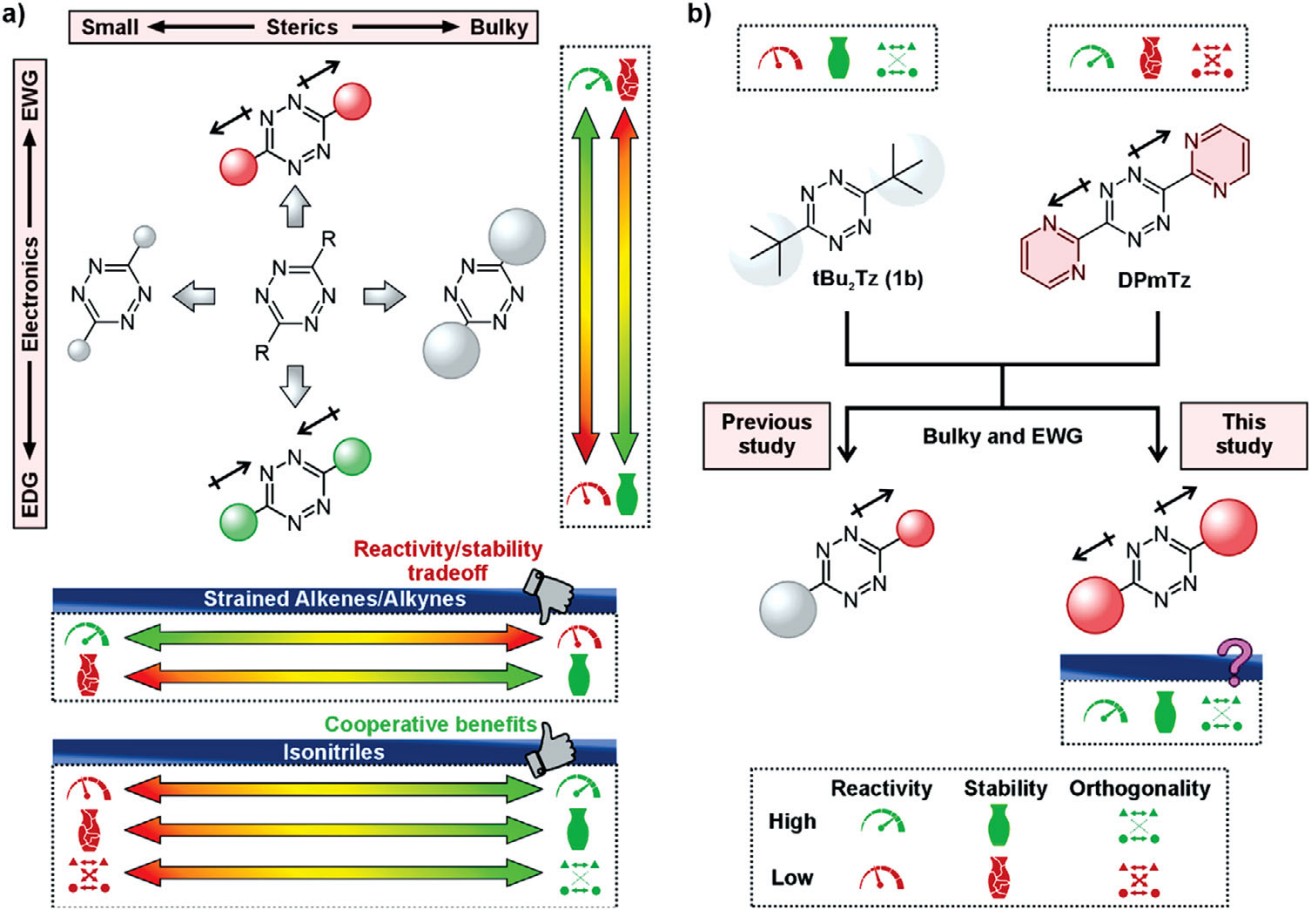
Effects of substituents on the reactivity of Tz in IEDDA reactions. a) The reactivity/stability trade‐off in Tz design for bioorthogonal chemistry: EWGs increase Tz reactivity but make them prone to degradation, while EDGs reduce reactivity. Bulky substituents stabilize Tz but decrease reactivity with alkenes/alkynes; however, bulky substituents accelerate reactions with isonitriles, shield Tz from decomposition, and support orthogonal reaction schemes. b) This study investigates Tz with aliphatic substituents that combine bulkiness and electronegativity toward the goal of achieving high reactivity, stability, and chemoselectivity.

Isonitriles present an opportunity to overcome the reactivity/stability tradeoff in Tz‐based reactions.^[^ ^27^, ^28^ ^]^ The isocyano group^[^ ^29^ ^]^ undergoes a [4 + 1]‐cycloaddition reaction with Tz, yielding pyrazoles.^[^ ^13^, ^14^ ^]^ The compactness and unique reactivity of the isocyano group make it ideal for probing proteins^[^ ^30^, ^31^ ^]^ and carbohydrates.^[^ ^14^, ^32^ ^]^ In contrast to alkenes/alkynes, strain cannot be incorporated to enhance the reactivity of isonitriles, requiring improvement of Tz reactivity. Remarkably, isonitriles react faster with Tz having bulky substituents.^[^ ^33^ ^]^ Understanding the interactions responsible for this effect is of high interest because it may lead to a better understanding of physical organic chemistry of isonitriles and uniquely allow for simultaneous improvement of reactivity, stability, and chemoselectivity of bioorthogonal reagents (Figure 1a).

Studies on the substituent effects of Tz reactions with isonitriles have primarily focused on aryl groups and simple alkanes. Here, we investigate the reactivity of Tz with aliphatic substituents that incorporate electron‐withdrawing groups (EWGs).

We hypothesized that Tz with bulky and electron‐withdrawing substituents would exhibit high reactivity toward isonitriles and maintain high orthogonality to other dienophiles, while the bulky substituent would provide shielding of the Tz ring. To explore substituent‐reactivity trends, we synthesized symmetrical dialkyl‐Tz with benzylic EWGs (Figure 2a; Table S1 and S2).

**Figure 2:**
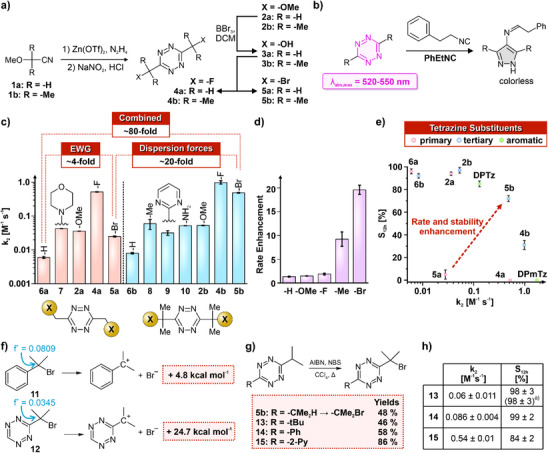
Effect of aliphatic substituents bearing electron‐withdrawing groups (EWGs) on the reactivity and stability of tetrazines (Tz). a) Synthesis of representative Tz. b) Disappearance of Tz absorbance band for measuring reaction rates. c) Impact of the introduction of EWGs and *gem*‐dimethyl groups on the reactivity of Tz toward PhEtNC (DMSO: PBS, 4:1, pH = 7.4, *T* = 25 °C). EWGs significantly enhance the reactivity of Tz with isonitriles, while the influence of *gem*‐dimethyl groups depends on the EWG. d) Rate‐acceleration by *gem*‐dimethyls on the reaction rate. The polarizability of the benzylic substituents influences the rate‐accelerating effect of *gem*‐dimethyl groups. e) Dependency of stability and isonitrile‐reactivity of Tz. *gem*‐Dimethyl groups shield the Tz, achieving satisfactory stability for highly reactive Tz such as **5b**. f) Reaction free energies for the heterolytic C─Br bond cleavage and Fukui electrophilicity indices for benzylic carbon atoms in model compounds **11** and **12**. The calculations suggest that ─CMe_2_Br group on Tz are inert to nucleophilic substitution. g) Access of different Tz with ─CMe_2_Br substituents through Wohl–Ziegler bromination. h) Tuning of rate constants with isonitrile (DMSO: PBS, 4:1, pH = 7.4, *T* = 25 °C, PhEtNC) and stability after 12 h by combining 2‐bromoprop‐2‐yl groups with different substituents. S_12h: Stability of compound quantified by remaining compound after 12 h._^a)^_Stability in 80% PBS and 20% DMSO._

EWGs increase the reactivity of Tz in IEDDA reactions,^[^ ^21^ ^]^ but the effect of aliphatic substituents with EWGs is unknown. Monitoring the disappearance of the n→π* transition of Tz upon their reaction with phenylethyl isonitrile (PhEtNC; Figure 2b) confirmed the rate‐accelerating impact of such substituents. Fluoromethyl groups (**4a**) accelerated the reaction rate by 87‐fold compared to dimethyl tetrazine (**4a**; k_2_ = 0.52 ± 0.012 M^−1^ s^−1^ versus **6a**; k_2_ = 0.006 ± 0.0004 M^−1^ s^−1^; Figure 2c). Other substituents also enhanced reaction rates, with the magnitude influenced by their electronic properties; the ─CH_2_OMe derivative (**2a**) reacted 9.6‐fold faster, the ‐CH_2_Br derivative (**5a**) reacted 4.1‐fold faster, and the morpholine derivative (**7**) reacted 7.2‐fold faster than **6a** (Figure 2c). This data establishes that aliphatic EWGs enhance the reactivity of Tz toward isonitriles.

Having verified the rate‐accelerating impact of aliphatic EWGs, we investigated the influence of benzylic methyl groups on the reaction with isonitriles. A prior study found that such methyl groups could enhance reactions through dispersion forces.^[^ ^33^ ^]^ We synthesized a series of Tz with 3°‐substituents containing benzylic EWGs and monitored their reaction with PhEtNC. Replacing the ─Me groups of **6a** with a ─tBu group (**8**) increased the reaction rate 10‐fold (Figure 2c,d). In contrast, the effect of *gem*‐dimethyl groups on the isonitrile reactivity was modest for benzylic ─OMe (k_2_[**2b**] = 0.053 ± 0.001 M^−1^ s^−1^) and ─F (*k*_2_[**4b**] = 0.98 ± 0.01 M^−1^ s^−1^) groups, resulting in only a 1.5‐fold and 1.9‐fold increase in the rate compared to Tz with 1°‐substituents **2a** and **4a**, respectively (Figure 2c,d).

An explanation for this outcome could be that the dispersion interactions between the fluorine and oxygen atoms and the isocyano group are low because of their small size and high electronegativity. If correct, *gem*‐dimethyl groups should significantly accelerate the reaction of Tz with ─CH_2_Br groups as bromine is highly polarizable. Indeed, **5b** (*k*_2_ = 0.49 ± 0.014 M^−1^ s^−1^; Figure S1) exhibited a notable 20‐fold rate acceleration compared to **5a** (Figure 2c,d). The rate of reaction of **5b** was comparable to that of the fluorine derivative **4b** and was ∼80‐fold higher than that of **6a**. This result highlights the significant contribution of the benzylic substituents to potential dispersion interactions.

The synergistic rate acceleration through combining bulky and EWG prompted an exploration of whether *gem*‐dimethyl groups could shield Tz (Figure 2e). As expected, EWGs heightened the susceptibility of Tz to decomposition in the presence of water. For instance, **4a** and **5a** degraded within minutes (4:1 DMSO:PBS, pH 7.4, *T* = 25 °C). The introduction of *gem*‐dimethyl groups enhanced Tz stability, and the stability of **5b** was significantly higher than that of **5a** despite its 20‐fold faster reaction with isonitriles. For comparison, under these conditions, the reactivity of **5b** was slightly lower than that of the widely used 3,6‐dipyridyltetrazine (DPTz) but higher than 3,6‐dipyrimidyl tetrazine (DPmTz; Figures 2e, S4, and S5). This outcome underscores the validity of the outlined design principle.

The presence of the ─CMe_2_Br group in compound **5b** may raise concerns for use in biological contexts because of the susceptibility of bromoalkanes to nucleophilic substitution. However, we had reason to believe that this group would exhibit good stability as a Tz substituent. Steric hindrance disfavors S_N_2 reactions at tertiary carbons, and the electron‐withdrawing nature of the Tz ring makes the carbocation intermediate required for an S_N_1 reaction energetically inaccessble. To gain insight into the susceptibility of **5b** to degradation via nucleophilic attack on the bromoalkane, we compared model Tz **12** to the corresponding phenyl derivative **11** (Figure 2f). To evaluate the relative electrophilicity of the bromide site in **12**, we calculated orbital‐weighted Fukui f^+^ indices using Multiwfn 3.8, which quantify susceptibility to nucleophilic attack. **12** exhibits a significantly lower f⁺ value (0.0345) compared to the phenyl analogue (0.0809). As both molecules are sterically similar, this result suggests that **12** is even more resistant to S_N_2 reactions than **11**, which is known to be resistant to S_N_2 reactions. Regarding S_N_1 reactions, calculated free energies for heterolytic C─Br bond cleavage (Figure 2f) indicate that the electron‐withdrawing Tz ring strongly disfavors ionization, supporting the high stability of the bromide functionality.

Beyond the stability of the bromoalkyl substituent, the stability of the Tz ring is a critical parameter. Unsurprisingly, the stability of **5b** decreased with increasing water content, with a decomposition rate constant of *k*_1_ = 3.0 × 10^−4^ s^−1^ in 80% PBS (See Figure S2 and S3). We, therefore, explored whether the ─CMe_2_Br group could be combined with other substituents in asymmetric Tz to tune reactivity and stability. This effort first required a more efficient synthetic route. Encouragingly, Wohl–Ziegler bromination of isopropyl‐1,2,4,5‐tetrazines proceeded smoothly, enabling the straightforward synthesis of asymmetric Tz pairing *tert*‐butyl (**13**), phenyl (**14**), and pyrid‐2‐yl (**15**) groups with ─CMe_2_Br (Figure 2g).

The reactivity of **15** was comparable to that of **5b** with a slightly higher stability, demonstrating the possibility of using a single ─CMe_2_Br group to achieve favorable reactivity. No measurable decomposition was observed for compounds **13** and **14**, including for **13** even in 80% PBS, although this was accompanied by reduced reactivity (Figure 2h). These data confirm that Tz stability is governed by the electronic properties of the heterocyclic ring, with no intrinsic instability associated with the ─CMe_2_Br substituent. Importantly, **15** integrates the previous strategy of combining bulky groups with EWGs with the one presented here of combining bulkiness and EWGs into one substituent (Figure 1). These data establish that the outlined strategy enables the reactivity/stability tuning of Tz.

A computational study using B3LYP‐D3(BJ)/6–311 + G(d,p) in CPCM(water) was conducted to shine light on the difference in behavior for fluoroalkyl and bromoalkyl Tz **4a/b** and **5a/b** (Figure 3). A detailed description of the computational methods can be found in the Supporting Information. Enthalpies of activation are in excellent agreement with experimental results and show that the barrier is only slightly reduced when going from **4a** to **4b** (−0.5 kcal mol^−1^) while the change from **5a** to **5b** shows a significant reduction in barrier height (−2.1 kcal mol^−1^; Table S3). In agreement with a prior study,^[^ ^33^ ^]^ increased dispersion interaction was identified as the main factor for the decrease in activation energies. Intermolecular dispersion interaction ∆*E*_disp_ increased from −5.23 to −6.84 kcal mol^−1^ for the fluoro derivatives and from −5.44 to −8.55 kcal mol^−1^ for the bromo derivatives (Table S3). Recently, Baldinelli and coworkers proposed a method to assess the atomic contributions to London dispersion energy.^[^ ^34^ ^]^ We use an extension of this method (see Supporting Information page S24), which allows us to obtain the atomic contributions of the intermolecular dispersion interaction. Figure 3 lists these atomic contributions next to the atoms, with contributions of hydrogen atoms summed into the corresponding carbon atom. Contributions of the Tz heterocycle are provided as the sum of all ring atoms.

**Figure 3:**
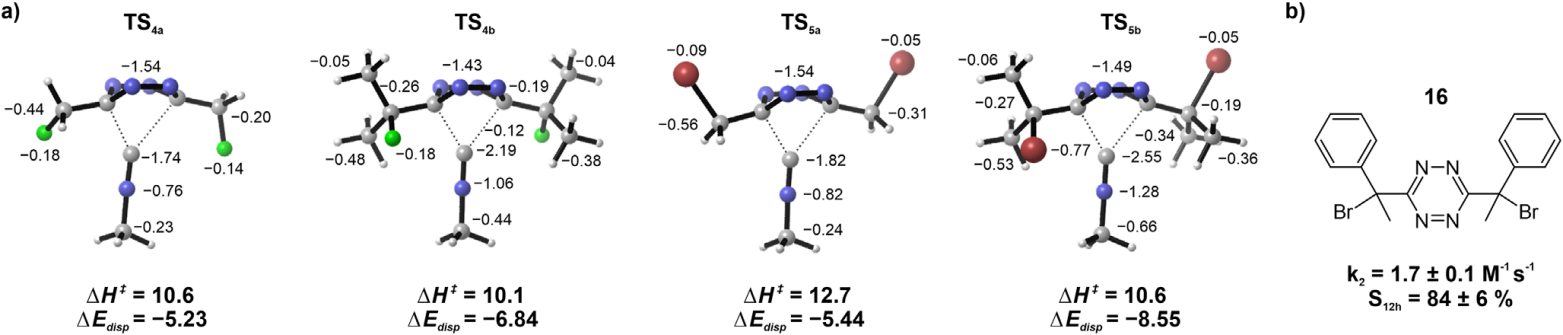
Effect of dispersion forces and conformation on reactivity. a) Computational investigations of the reaction between **4a**, **4b**, **5a**, and **5b** with the model isonitrile methyl isocyanide. Energies are shown in kcal mol^−1^. Labels at the structures indicate the atomic contribution to the intermolecular dispersion interaction. Contributions of hydrogens were summed into corresponding carbon atoms. The label above the ring structure denotes the sum of interaction for all six tetrazine atoms. b) Introducing phenyl groups into the tetrazine substituents enhances reactivity toward isonitriles (DMSO: PBS, 4:1, pH = 7.4, *T* = 25 °C, PhEtNC), likely by locking the bromo groups into a *gauche* conformation.

In TS_5a_, both bromine atoms point upwards and away from the isonitrile not contributing significantly to the intermolecular dispersion interaction. Meanwhile, in TS_5b_ one of the bromine atoms is moved closer to the isonitrile because a methyl group is taking the position the bromine occupied in TS_5a_. Our analysis shows that a bromine atom contributes roughly twice as much as a methyl group (−0.77 versus approx. −0.4 kcal mol^−1^) in this orientation, while substituents pointing away from the isonitrile do not contribute. The strong increase in dispersion interaction from **5a** to **5b** is caused by switching from no group pointing down in TS_5a_ to three methyl and one bromine being in proximity to the isonitrile. In contrast to TS_5b_, in TS_4b_, the preferred transition state conformer has both halogen atoms pointing downwards. However, the smaller and less polarizable fluorine contributes much less to dispersion interactions than bromine, or even methyl groups. In fact, methyl groups show interactions that are about twice as strong as those of fluorine atoms. Additionally, even in TS_4a_ fluorine groups are pointing toward the isonitrile, thus the difference between these two transition states is even lower as only the interaction of two nearby methyl groups is added.

This investigation highlights the importance of both the nature and orientation of substituents in accelerating bioorthogonal cycloadditions through dispersion interactions. While atoms like bromine allow for stronger dispersion interactions compared to fluorine or methyl groups, the orientation of these substituents is critical. Given these insights, further enhancement of Tz reactivity might be achieved by enforcing a *gauche* conformation of the bromo group. This could be accomplished by introducing a bulky substituent that favors a staggered orientation. To test this hypothesis, we synthesized Tz **16** bearing two 1‐bromo‐1‐phenylethyl substituents. The reaction rate of **16** and PhEtNC was measured at 1.7 ± 0.1 M^−1^ s^−1^, clearly surpassing that of **5b**. As the electronic properties of the two substituents are comparable, this result reinforces the role of dispersion forces in accelerating Tz‐isonitrile reactions and provides direct evidence for the impact of conformation on reactivity. These findings suggest that controlling the orientation of dispersion‐contributing groups offers a promising strategy for optimizing such cycloadditions.

Water content and elevated temperatures both accelerate the Tz‐isonitrile reaction.^[^ ^35^ ^]^ Under physiologically relevant conditions (80% PBS:10% DMSO at 37 °C), the bimolecular rate constants for **5b** reacting with PhEtNC reached *k*_2_ = 16 M^−1^ s^−1^ and for trimethylsilylmethyl isonitrile (TMSMeNC) *k*_2_ = 31 M^−1^ s^−1^ (Figure 4a). These rates surpassed those observed for reactions involving widely used DPTz by 6‐fold and are only slightly lower than those of the most reactive asymmetric Tz bearing *tert*‐butyl and pyrimidyl substituents^[^ ^33^ ^]^ while offering the advantage of easier synthesis. The bimolecular reaction reached completion well before significant Tz decomposition occurred. Therefore, **5b** exhibits bimolecular rates with isonitriles comparable to many established bioorthogonal reactions.

**Figure 4:**
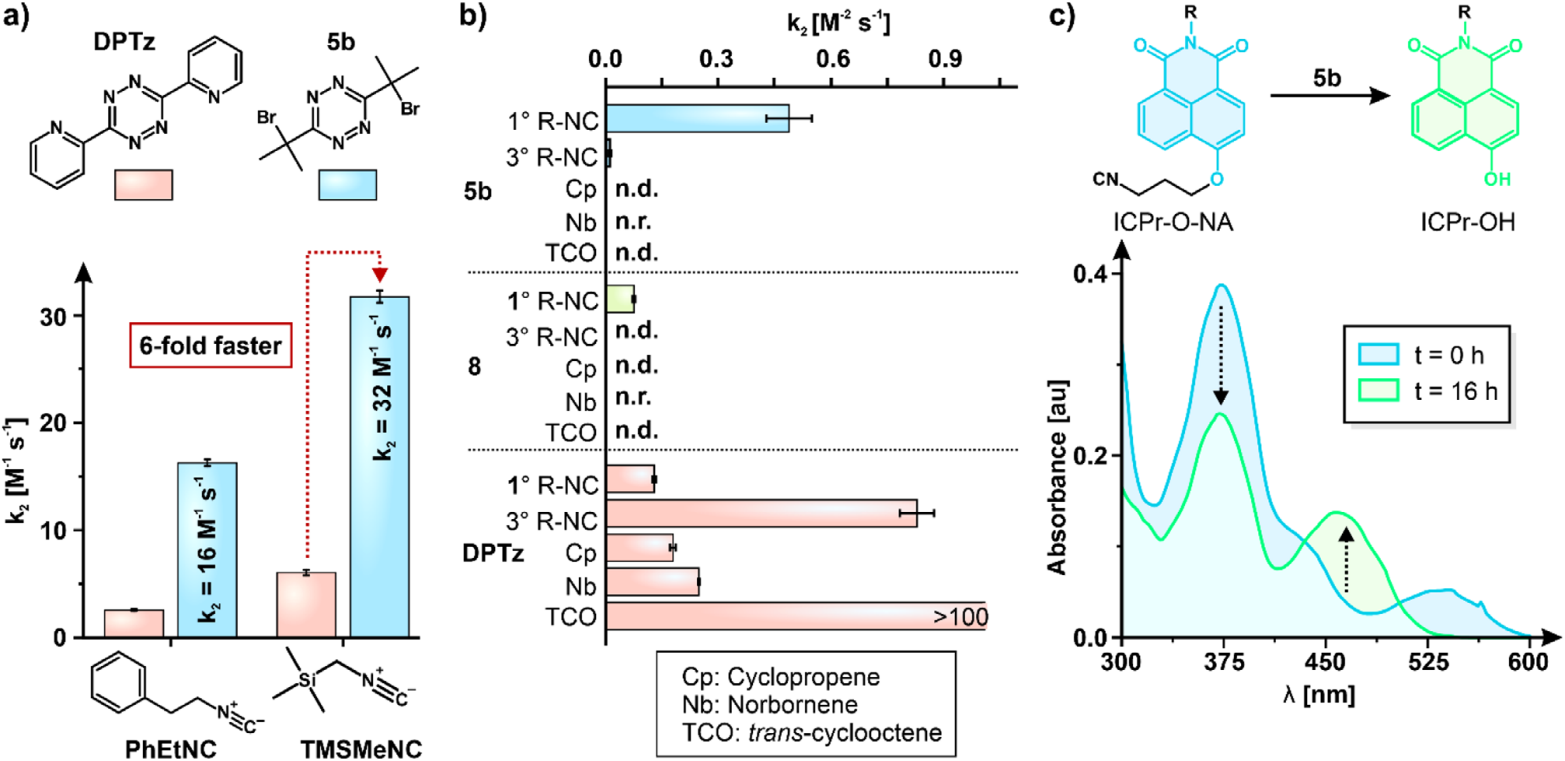
Assessment of **5b** in bioorthogonal scenarios. a) Bimolecular reaction rates of **5b** and isonitriles PhEtNC and TMSMeNC under physiologically relevant conditions (4:1 PBS:DMSO; pH = 7.4; *T* = 37 °C) and a comparison of rates with the widely used DPTz. The reaction rate between **5b** and isonitriles is comparable to other bioorthogonal reactions and those of isonitriles with other Tz. b) Reactivity of **5b** across dienophiles. **5b** exhibits preference for 1° isonitriles with substantial orthogonality towards other dienophiles and 3° isonitriles, comparable to that for the slower‐reacting **8**. Orthogonality of DPTz is low, and 3°‐isonitriles are preferred over 1°‐isonitriles (DMSO:H_2_O; 4:1, pH = 7.4, *T* = 25 °C; n.d.: rate not determinable due to slow reaction; n.r.: no reaction observed). c) Uncaging of 3‐isocyanopropyl groups by **5b**. Changes in the UV–vis absorbance spectrum of ICPr‐O‐NA (0.1 mM) in the presence of **5b** (0.3 mM; 1:1 DMSO‐PBS, pH = 7.4, *T* = 37 °C) confirm the liberation of the dye.

A favorable characteristic of Tz with bulky substituents is their selective reactivity with isonitriles while being inert toward other dienophiles.^[^ ^33^ ^]^ This selectivity, combined with the orthogonality of isonitriles to certain dienes,^[^ ^36^ ^]^ makes this reaction ideal for developing pairs of compatible bioorthogonal reactions. Intrigued by the reactivity of **5b**, we tested its orthogonality toward other dienophiles. Indeed, **5b** exhibited negligible reactivity with *trans*‐cyclooctene, norbornene, and cyclooctyne (Figure 4b, Table S4). Even across isonitriles, **5b** demonstrates remarkable selectivity (Figure 4b). The reaction rates of **5b** with primary (PhEtNC) and secondary (CyHexNC; cyclohexyl isonitrile) isonitrile are comparable whit the secondary isonitrile (*k*_2_ = 0.59 ± 0.08 M^−1^ s^−1^) exhibiting a 1.2‐fold higher rate constant than PhEtNC. In contrast, the reaction of CyHexNC is 27.2‐fold faster than that with *tert*‐butyl isonitrile (^t^BuNC), which is the inverse trend observed for the reaction of isonitriles with DPTz (Table S4 and Figure S6). The combination of favorable orthogonality and reactivity makes these molecules promising candidates for the simultaneous implementation of multiple bioorthogonal reactions.^[^ ^4^, ^5^ ^]^

Tz‐isonitrile reactions have found application in the release of drugs,^[^ ^35^, ^37^ ^]^ gasotransmitters,^[^ ^38^ ^]^ fluorophores,^[^ ^39^, ^40^, ^41^ ^]^ and biomacromolecules.^[^ ^31^, ^42^ ^]^ We therefore tested whether **5b** could remove isocyanopropyl (ICPr) groups.^[^ ^35^ ^]^ Exposure of a ratiometric naphthalimide‐ICPr probe to **5b** resulted in changes in the absorbance spectrum consistent with ICPr removal (Figure 4c). Given that Tz with 3°‐aliphatic substituents form stable imine conjugates, the release with **5b** is gradual, consistent with previous observations involving **8**.^[^ ^43^ ^]^ Additionally, we tested the release of resorufin from a caged resorufin dye (ICPr‐Rsf) upon treatment with compound **5b** and monitored the fluorescence recovery kinetics. The fluorescence was fully recovered within 20 h (Figure S7), demonstrating the bioorthogonal release ability of **5b**.

To demonstrate that **5b** meets the criteria of bioorthogonality, we further investigated the uncaging of an ICPr‐caged resorufin dye (ICPr‐Rsf) in zebrafish embryos (Figures 5 and S7). ICPr‐Rsf was injected into the yolk sac of zebrafish embryos, followed by the injection of **5b** after a brief incubation period. The Tz elicited a strong fluorescence signal localized to the yolk sac, whereas embryos injected with ICPr‐Rsf and DMSO control showed low fluorescence (Figures 5 and S6). The relative fluorescence increase in the yolk sac was 6.7‐fold (p ≤ 0.0001). These experiments confirm that **5b** is effective in releasing molecules of interest in vivo. To test for adverse effects of **5b**, it was injected into the yolk sac of zebrafish embryo, and heart rate was measured as a metric of physiological impact. A dose‐dependent decrease in heart rate was observed immediately after injection, but this effect normalized within a few hours (Figure S8).

**Figure 5:**
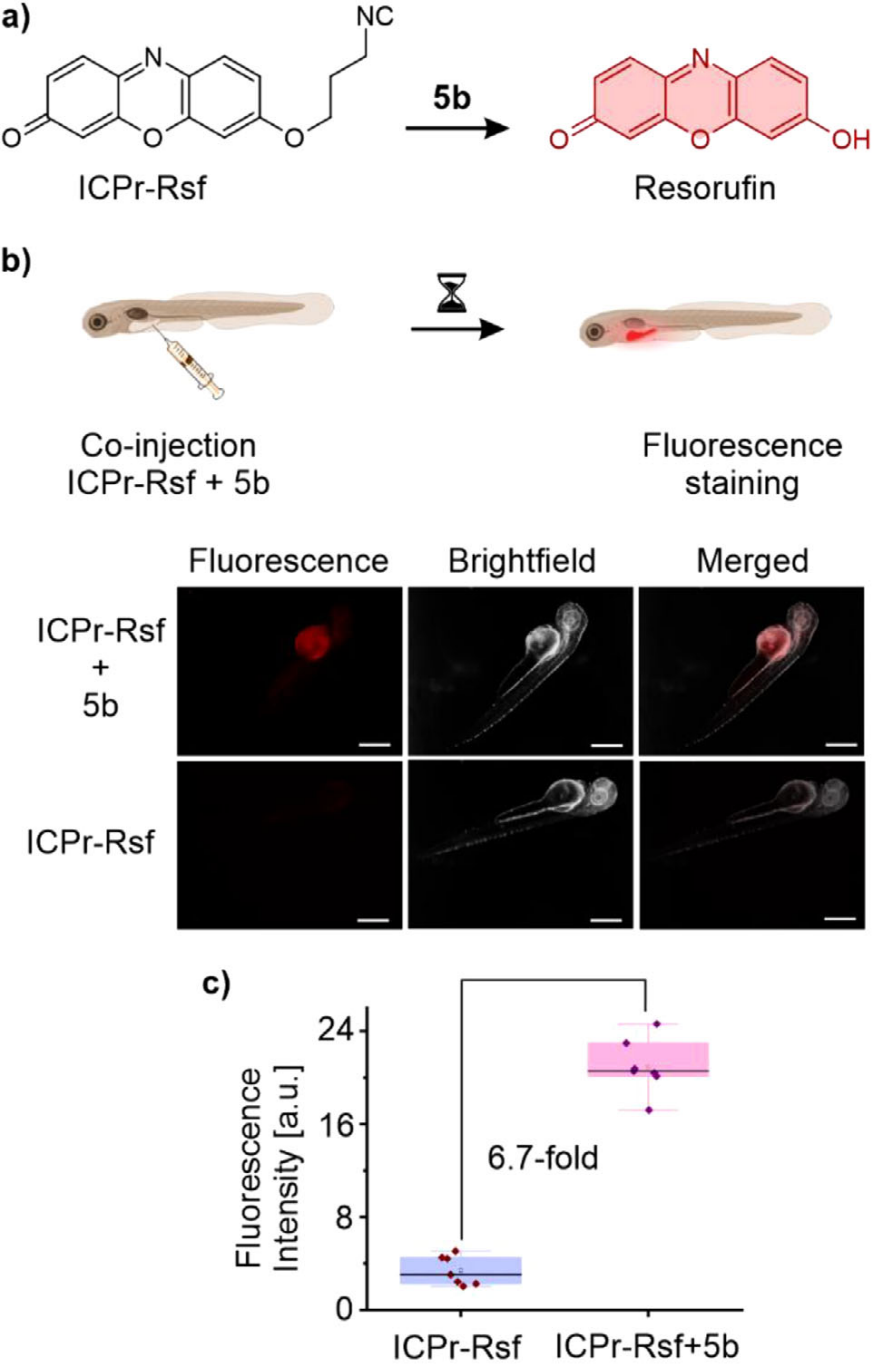
Biocompatibility of the developed tetrazines was demonstrated in zebrafish embryos. a) Schema depicting structures of the resorufin pro‐fluorophore masked with a 3‐isocyanopropyl group (ICPr‐Rsf) and uncaged resorufin. b) Injection of ICPr‐Rsf into the zebrafish embryos yolk sac followed by the injection of tetrazine **5b**. Visualization of the fluorescence signal from un‐caged resorufin in live zebrafish (scale bar = 500 µm) co‐injected with 3 nL of ICPr‐Rsf (3.5 mM) and **5b** (0.35 mM) after 2 h of incubation. c) Treatment with **5b** resulted in a 6.7‐fold increase in resorufin fluorescence within the yolk sac compared to embryos injected with ICPr‐Rsf but not treated with **5b**.

In conclusion, this study demonstrated that aliphatic substituents that are both bulky and electron‐withdrawing significantly enhance the reactivity of Tz toward isonitriles. Computational studies highlighted the importance of dispersion forces between the benzylic bromine substituents and the incoming isonitrile, with a favorable molecular conformation being key to maximizing this effect. The optimized compound **5b** exhibited reaction rates with isonitriles as high as 32 M^−1^ s^−1^, surpassing traditional Tz like dimethyltetrazine and DPTz, and comparable to other widely used bioorthogonal reactions such as strain‐promoted azide‐alkyne cycloadditions. Additionally, these Tz retain high orthogonality to strained alkenes and can effectively deprotect ICPr groups in living organisms. Future studies will aim to leverage the insights gained here to explore potential biological applications of these tetrazines. The demonstrated stability of the ─CMe_2_Br group as a Tz substituent, along with its straightforward incorporation into asymmetric tetrazines via Wohl–Ziegler bromination, represent important steps toward this goal. This study not only provides a foundation for designing more reactive bioorthogonal reagents but also advances our understanding of rate acceleration through bulky substituents and dispersion forces.

## Supporting Information

The authors have cited additional references within the Supporting Information.^[^ ^33^, ^34^, ^37^, ^43^, ^44^, ^45^, ^46^, ^47^ ^]^

## Conflict of Interests

The authors declare no conflict of interest.

## Supporting information



Supporting Information

## Data Availability

The data that support the findings of this study are available from the corresponding author upon reasonable request.
