# Supplementary material for: Combined Inductive and Dispersion Effects Enhance Bioorthogonal Reactivity of Tetrazines Toward Isonitriles
Source: Angew Chem Int Ed Engl. 2025 Aug 10;64(39):e202501235. doi: 10.1002/anie.202501235 (PMC12455400; doi:10.1002/anie.202501235)
Supplement: Supplementary file 1 — Supporting Information [file ANIE-64-e202501235-s001.pdf]

Supporting Information

Combined Inductive and Dispersion Effects Enhance Bioorthogonal Reactivity of Tetrazines Toward Isonitriles

Suprakash Biswas,[a] Andreas Löffler,[b] Pushkar Bansal,[c] Randall T. Peterson,[c] Dennis Svatunek,[b] and Raphael M. Franzini*[a,d]

[a] Dr. Suprakash Biswas, Prof. Dr. R. M. Franzini
Department of Medicinal Chemistry
College of Pharmacy
30 S 2000 E, Salt Lake City, UT 84112, USA
E-mail: Raphael.franzini@utah.edu

[b] A. Löffler, Dr. D. Svatunek
Institute of Applied Synthetic Chemistry
TU Wien
Getreidemarkt 9, 1060 Vienna, Austria

[c] Dr. P. Bansal, Prof. Dr. R. T. Peterson
Department of Pharmacology and Toxicology
College of Pharmacy
30 S 2000 E, Salt Lake City, UT 84112, USA

[d] Prof. Dr. R. M. Franzini
Huntsman Cancer Institute
2000 Circle of Hope, Salt Lake City, UT 84112, USA

Table of Contents:

Materials and instrumentation 3

Methods computational analysis 3

Ethical Approval Statement for Animal Experiments 3

Synthetic procedures 4-13

Photo-spectrometric analysis of reaction kinetics 14-21

Photo-spectrometric stability measurement of the tetrazines 21

HPLC analysis for stability measurement of 3,6-bis(2-bromopropan-2-yl)-1,2,4,5-tetrazine 22

Computational analysis for reaction rate 23-24

Photospectrometric analysis of tetrazine orthogonality 25-26

Effects of 1°, 2°, 3° Isonitriles on reactivity against tetrazine 27

Photospectrometric analysis of resorufin release 28

Physiological impact of 3,6-bis(2-bromopropan-2-yl)-1,2,4,5-tetrazine on zebrafish 29

References 30

^{1}H NMR of synthesized compounds 31-40

^{19}F-NMR of the synthesized compounds 41

^{13}C-NMR of the synthesized compounds 42-50

Mass spectrometry data of the synthesized compounds 51-54

Cartesian co-ordinates of TS for theoretical calculations 55-61

Materials and Instrumentation

All chemical reagents and solvents were obtained from commercial sources (Sigma-Aldrich, Alfa-Aesar, Combi-Blocks, Oakwood Chemical, Enamine, Acros-Organic, RPI, TCI) and used without further purification. BBr_3 solution in DCM (1 M) was purchased from Aldrich and directly used. Thin-layer chromatography (TLC) analysis was carried out to monitor the progress of reactions. The synthesized compounds were purified using silica gel 300-400 mesh column chromatography. ^{1}H-NMR and ^{13}C-NMR spectra were recorded on a Varian Mercury-400 or Varian Inova-500 spectrometer as indicated, with chemical shifts expressed as ppm (in $CDCl_3$, or DMSO-d_6) using Me_4Si (TMS) as an internal standard. High-resolution mass spectra were measured using the Mass Spectrometry and Proteomics Core Facility at the University of Utah. Mass spectrometry equipment was obtained through a Shared Instrumentation Grant 1 S10 OD018210 01A1 for the University of Utah. UV-Vis. photo-spectrometic kinetic measurements were performed with a SpectraMax M5 (Molecular Device, USA) in 96-well plates or 3 mL quartz cuvettes. For the spectroscopic measurement, DMSO and Phosphate buffer saline (PBS, pH 7.4) were analytical grade, purchased from commercial sources. All analytical HPLCs were performed on a Dionex Ultimate3000 equipped with an autosampler, diode array detector and robotic fraction collector (Dionex Thermo Scientific, USA) using a LUNA C18 column (5 μM, 150×2.0 mm, Phenomenex, USA). Zebrafish imaging was performed with Zeiss SteREO Discovery.V8 microscope (Zeiss, Jena, Germany) fitted with a PentaFluor S 120 vertical illuminator and coupled with an Xcite Series 120PC light source.

Methods computational analyses

Gaussian16 A.03 was used for DFT calculations. The B3LYP functional was used together with the 6-311+G(d,p) basis set. The implicit CPCM model was used to include the solvent effects of water implicitly. D3 dispersion correction using BJ dampening was employed to model dispersion effects. This method has previously been shown to recreate relative barriers of similar reactions with good accuracy.[1] Conformer searches were conducted using CREST 2.12 together with XTB 6.6.1 and default settings.[2, 3] For transition state conformer searches, both forming bonds were frozen. All conformers were reoptimized at the DFT level. The lowest energy conformers were used for the analysis of dispersion interactions. Coordinates are provided as xyz-files.

Ethical Approval Statement for Animal Experiments

All animal experiments were conducted in accordance with protocols approved by the Institutional Animal Care and Use Committee (IACUC) at the University of Utah. The zebrafish studies described in this work were performed under IACUC Protocol #00001487 (Principal Investigator: Randall Peterson). The University of Utah is accredited by AAALAC International.

Synthetic Procedures

3,6-di(pyridine-2-yl)-1,2,4,5-tetrazine (DPTz) was purchased from Alfa Aesar and used without further purification. 3,6-dimethyl-1,2,4,5-tetrazine (6a), 3,6-diisopropyl1,2,4,5-tetrazine (6b), 3,6-di-tert-butyl-1,2,4,5-tetrazine (8), 3,6-di(pyrimidine-2-yl)-1,2,4,5-tetrazine (DPmTz) and the caged fluorophores *N*-(methyl-PEG4)-4-(3-isocyanopropyl-1-oxy)-1,8-naphthalimide (ICPr-O-NA), 3-isocyanopropyl resorufin ether (ICPr-Rsf) were synthesized as previously reported and their characterizations were consistent with reported data.[1, 4]

General Synthetic Procedure A

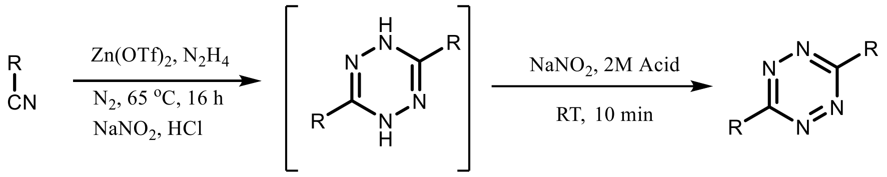

The general synthetic procedure was adapted from the procedure reported by Devaraj *et al.*[5]

To an oven-dried reaction flask equipped with a stir bar, the nitrile (1 eq.) was added together with solid zinc triflate ($Zn(OTf)_2$, 0.05 eq.). A minimal amount of dry dioxane was added to dissolve the $Zn(OTf)_2$ in that mixture. The flask was sealed and kept under an inert atmosphere. Anhydrous hydrazine (5 eq.) was added dropwise to the stirred solution at 0°C. After completion of the addition, the reaction flask was sealed and stirred at 65°C for 16 hours. Upon complete consumption of the starting material, sodium nitrite (10 eq.) in water at a final solution of 20% (w/v), was slowly added to the reaction mixture cooled to 0°C. The dropwise addition of concentrated aqueous HCl (or different acid used as stated in procedure) at 0°C caused the solution to turn red and induced the evolution nitrogen oxide (Caution: toxic). Addition of concentrated aqueous HCl was stopped once gas evolution ceased and the pH reached ~3. The resulting solution was brought to room temperature and stirred for another 10 minutes. The mixture was extracted with DCM. The combined organic layers were washed with brine, dried over Na_2SO_4 or $MgSO_4$, filtered and evaporated. Finally, the crude compounds were purified by silica gel column chromatography to afford the desired product.

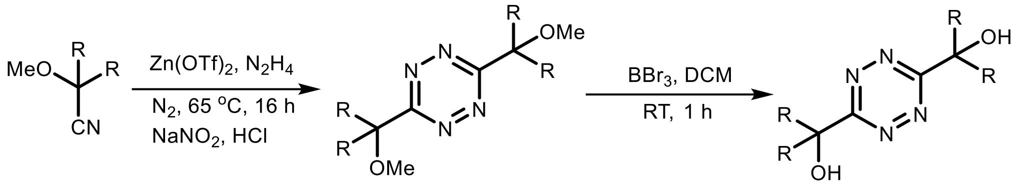

***3,6-bis(methoxymethyl)-1,2,4,5-tetrazine* (2a):** Synthesized following general procedure A. Methoxyacetonitrile (200 mg, 2.01 mmol), anhydrous N_2H_4 (633 µL, 21.1 mmol), and $Zn(OTf)_2$ (76.5 mg, 0.21 mmol), were reacted as described in 2 mL of dry dioxane. The product was purified with EtOAc/Hexanes (3:7) (R_f = 0.3). The desired product **2a** was isolated as a deep purple liquid in a yield of 130 mg (38%). ^{1}H-NMR (400 MHz, $CDCl_3$) δ 5.10 (s, 4H), 3.62 (s, 6H) ppm. ^{13}C NMR (100 MHz, $CDCl_3$) δ 167.41, 72.12, 59.71 ppm. ESI-HRMS M/z [M+Na$^+$] calculated for $C_6H_{10}N_4O_2Na$ is 193.070 Da and found 193.069 Da.

***3,6-bis(2-methoxypropan-2-yl)-1,2,4,5-tetrazine* (2b):** Synthesized following general procedure A. 2-Methoxy-2-methylpropanenitrile (300 mg, 4.22 mmol), dry N_2H_4 (320 µL, 10 mmol), and $Zn(OTf)_2$ (36.3 mg, 0.1 mmol), were reacted as described in 2 mL of dry dioxane. The product was purified by column chromatography with EtOAc/hexane (1:5) (R_f = 0.3). The desired product **2b** was isolated as a deep purple liquid in a yield of 168 mg (37%). ^{1}H NMR (400 MHz, $CDCl_3$) δ 3.32 (s, 6H), 1.84 (s, 12H). ^{13}C NMR (100 MHz, $CDCl_3$) δ 171.68, 77.61, 51.74, 25.66 ppm. ESI-HRMS M/z [M+H$^+$] calculated for $C_{10}H_{19}N_4O_2$ is 227.150 Da and found 227.150 Da

***3,6-bis(hydroxymethyl)-1,2,4,5-tetrazine* (3a):** In an oven-dried round bottom flask, compound **2a** (80 mg, 0.46 mmol) was dissolved in dry DCM (5 mL). A 1 M BBr_3 solution in DCM (1.4 mL, 1.4 mmol) was added dropwise at 0°C over 10 minutes. The reaction was then allowed to proceed at room temperature for 1 hour. After completion of the reaction as determined by TLC monitoring, it was quenched with 0.5 mL of MeOH added at 0°C, which caused the brown reaction mixture to turn purple. The reaction mixture was diluted with DCM, and washed with brine. The brine wash solution was re-extracted with DCM. The combined organic layers were dried over anhydrous Na_2SO_4, filtered, and purified by silica gel column chromatography using EtOAC/hexanes (2:3) (R_f = 0.2), affording the desired product **3a** as a red solid in a yield of 18 mg (27%). ^{1}H NMR (400 MHz, $CDCl_3$) δ 4.97 (s, 4H) ppm.[6]

***3,6-bis(2-hydroxyprop-2-yl)-1,2,4,5-tetrazine* (3b):** In an oven-dried round bottom flask, compound **2b** (226 mg, 1 mmol) was dissolved in dry DCM (4 mL). A 1 M BBr_3 solution in DCM (4 mL, 4 mmol) was added dropwise at 0°C over 10 minutes. The reaction was then allowed to proceed at room temperature for 1 hour. After completion of the reaction as determined by TLC monitoring, it was quenched with 0.5 mL of MeOH added at 0°C, which caused the brown reaction mixture to turn purple. The reaction mixture was washed with brine. The brine wash solution was re-extracted with DCM several times. The combined

organic layers were dried over anhydrous Na_2SO_4, filtered, and purified by silica gel column chromatography using EtOAC/hexanes (1:4) (R_f = 0.2), affording the desired product **3b** as a red solid in a yield of 61 mg (31%). ^{1}H NMR (400 MHz, $CDCl_3$) δ 3.70 (s, 2H), 1.84 (s, 12H) ppm. ^{13}C NMR (100 MHz, $CDCl_3$) δ 173.5, 73.0, 29.5 ppm. ESI-HRMS M/z [M+Na$^+$] calculated for $C_8H_{14}N_4O_2Na$ is 221.101 Da and found 221.100 Da.

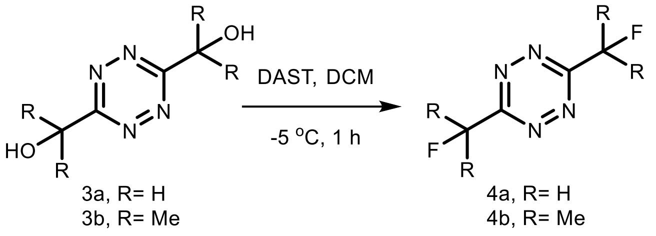

***3,6-bis(fluoromethyl)-1,2,4,5-tetrazine* (4a):** Compound **3a** (90 mg, 0.63 mmol) was dissolved in dry DCM. Diethylaminosulfur trifluoride (DAST; 50% in DCM; 450 μL, 1.4 mmol) was added to the solution at -5°C. The reaction was allowed to proceed at -5°C for 30 minutes. After completion of the reaction as determined by TLC monitoring, it was quenched with saturated aqueous $NaHCO_3$. The organic layer was separated and the aqueous layer extracted with DCM. The combined organic layers were dried over anhydrous Na_2SO_4, filtered, and evaporated. The crude product was purified by silica gel flash chromatography using EtOAc/hexanes (1:9) (R_f = 0.4). The desired product **4a** was isolated as a deep purple solid in a yield of 27.3 mg (30%). ^{1}H NMR (400 MHz, $CDCl_3$) δ 6.07 (s, 2H), 5.95 (s, 2H) ppm. ^{13}C NMR (100 MHz, $CDCl_3$) δ 165.98, 81.63, 80.22 ppm. ^{19}F NMR (376 MHz, $CDCl_3$) δ -225.30 ppm.

***3,6-bis(2-fluoropropan-2-yl)-1,2,4,5-tetrazin*e (4b):** Compound **3b** (80 mg, 0.41 mmol) was dissolved in dry DCM. Diethylaminosulfur trifluoride (DAST; 50% in DCM; 293 μL, 0.91 mmol) was added to the solution -5°C. The reaction was allowed to proceed at -5°C for 30 minutes. After completion of the reaction as determined by TLC monitoring, it was quenched with saturated aqueous $NaHCO_3$. The organic layer was separated and the aqueous layer extracted with DCM. The combined organic layers were dried over anhydrous Na_2SO_4, filtered and evaporated. The crude product was purified by silica gel flash chromatography using EtOAc/hexanes (1:9) (R_f = 0.4). The desired product **4b** was isolated as a deep purple solid in a yield of 32 mg (38%). ^{1}H NMR (400 MHz, $CDCl_3$) δ 2.03 (s, 6H), 1.98 (s, 6H) ppm. ^{13}C NMR (100 MHz, $CDCl_3$) δ 170.43, 95.05, 93.30, 26.92 ppm. ^{19}F NMR (376 MHz, $CDCl_3$) δ -143.30. ESI-HRMS M/z [M+H$^+$] calculated for $C_8H_{13}N_4F_2$ is 203.110 Da and found 203.110 Da.

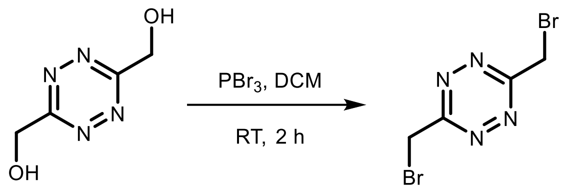

3a 5a

***3,6-bis(bromomethyl)-1,2,4,5-tetrazine* (5a):** In an oven-dried reaction flask, compound **3a** (50 mg, 0.35 mmol) was dissolved in anhydrous DCM (10 mL). To this solution under an argon atmosphere, PBr_3 (189 mg, 0.7 mmol) in dry DCM (5 mL) was added dropwise at 0°C. Upon completion of the addition, the ice bath was removed, and the mixture was allowed to stir for 2 hours at room temperature. After completion of the reaction as determined by TLC monitoring, the reaction was quenched by dropwise addition of ice-cold brine solution. The organic layer was separated and the aqueous layer extracted with DCM (25 mL × 3). The combined organic layers were dried over Na_2SO_4, filtered and evaporated. The crude product was purified by silica gel column chromatography using EtOAc/hexanes (1:9) (R_f = 0.5) to afford the desired product **5a** as a red solid in a yield of 45 mg (47%). ^{1}H NMR (400 MHz, $CDCl_3$) δ 4.97. (s, 4 H). ^{13}C NMR (101 MHz, $CDCl_3$) δ 167.66, 27.04 ppm.

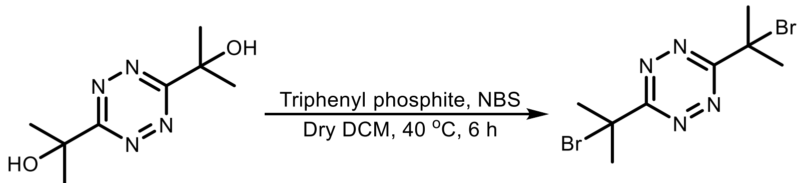

3b 5b

***3,6-bis(2-bromopropan-2-yl)-1,2,4,5-tetrazine* (5b) – protocol 1:** In a reaction vessel compound, **3b** (30 mg, 0.151 mmol) and NBS (107 mg, 0.6 mmol) were dissolved in of dry DCM (3 mL). Triphenyl phosphite (186 mg, 0.6 mmol) was added dropwise to the solution stirred at room temperature. The reaction mixture was heated to 40°C and stirred for 6 h at this temperature. After completion of the reaction as determined by TLC monitoring, the reaction mixture was filtered through a bed of celite, which was washed with ether. The organic layer was washed with water, dried over anhydrous Na_2SO_4, filtered, and evaporated. The residue was purified by silica gel column chromatography using EtOAc/hexane (1:19) (R_f = 0.5) to afford the desired product **5b** as a red solid in a yield of 5.8 mg (12%). ^{1}H NMR (400 MHz, CDC_3) δ 2.71 (s, 12H). ^{13}C NMR (101 MHz, $CDCl_3$) δ 171.54, 57.33, 32.06 ppm. ESI-HRMS M/z [M+H$^+$] calculated for $C_8H_{13}N_4Br_2$ is 324.948 Da and found 324.948 Da.

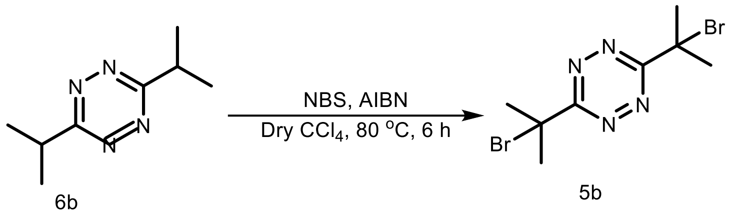

6b 5b

***3,6-bis(2-bromoprop-2-yl)-1,2,4,5-tetrazine* (5b) – protocol 2:** In a reaction vessel, 3,6-diisopropyl-1,2,4,5-tetrazine (**6b**; 83 mg, 0.5 mmol), was dissolved in dry CCl_4 (5 mL). Azobisisobutyronitrile (24.6 mg, 0.15 mmol) and *N*-bromosuccinimide (222 mg, 1.25 mmol) were added to the mixture, and the

reaction mixture was refluxed for 6 hours. After completion of the reaction as determined by TLC, the reaction mixture was allowed to cool to room temperature and a white precipitate was removed by filtration. The filtrate was evaporated under reduced pressure, and the crude mixture was purified using silica gel column chromatography using EtOAc/hexane (1:19) as the eluent (R_f = 0.5) to afford the desired product **5b** as a red solid in a yield of 65 mg (48%). ^{1}H NMR (400 MHz, CDC_3) δ 2.71 (s, 12H). ^{13}C NMR (101 MHz, $CDCl_3$) δ 171.54, 57.33, 32.06 ppm. ESI-HRMS M/z [M+H$^+$] calculated for $C_8H_{13}N_4Br_2$ is 324.948 Da and found 324.948 Da.

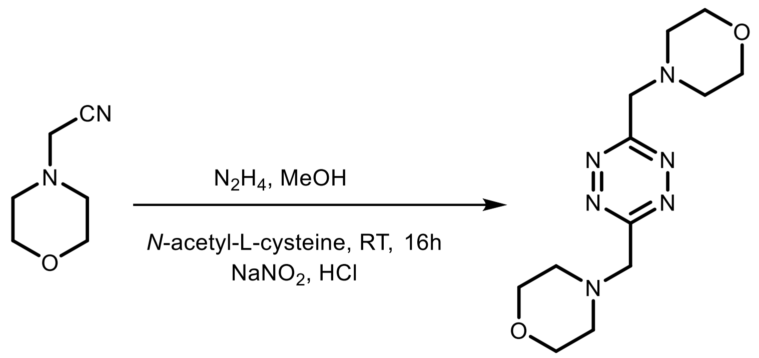

3,6-bis(morpholinomethyl)-1,2,4,5-tetrazine **(7):** Compound **7** was synthesized following a modified literature-reported procedure.[7] 4-morpholineacetonitrile (380 mg, 3 mmol) and *N*-acetyl-L-cysteine (490 mg, 3 mmol) were dissolved in methanol (3 mL). To the mixture at 0 °C was added hydrazine monohydrate (360 µL, 12 mmol) dropwise. The mixture was stirred at 50°C under an argon atmosphere for 16 hours. A white solid precipitated, which was filtered and re-dissolved in THF. Subsequently, a solution of $NaNO_2$ (1 g, 15 mmol) in 5 mL of water was added dropwise at 0°C, followed by the dropwise addition of AcOH until the evolution of gas ceased. The pH of the mixture was adjusted to pH 7 using dropwise addition of 4 M $NaHCO_3$ in water. The organic layer was separated and the aqueous layer extracted with DCM. The combined organic layers were dried over anhydrous Na_2SO_4, filtered, and evaporated under reduced pressure. The obtained red solid was washed with THF and dry ether to afford the desired product **7** in a yield of 89 mg (21 % yield). ^{1}H NMR (400 MHz, $CDCl_3$) δ 4.21 (s, 4H), 3.75 (dd, 8H, J = 8.20 Hz), 2.70 (dd, 8H, J = 8.12 Hz) ppm. ^{13}C NMR (101 MHz, $CDCl_3$) δ 163.31, 63.57, 57.19, 52.32 ppm. ESI-HRMS M/z [M+H$^+$] calculated for $C_8H_{21}N_6O_2$ is 281.172 Da and found 281.172 Da

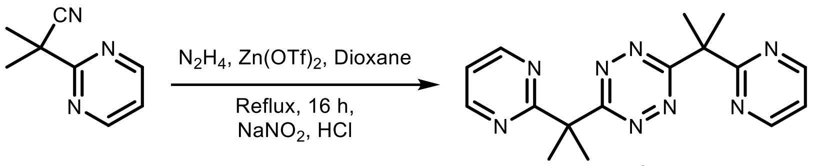

9

3,6-bis(2-(pyrimidin-2-yl)propan-2-yl)-1,2,4,5-tetrazine **(9):** Synthesized following the general procedure A. *α,α*-Dimethyl-2-pyrimidineacetonitrile (147 mg, 1 mmol), anhydrous N_2H_4 (150 µL, 5 mmol),

and $Zn(OTf)_2$ (18 mg, 0.05 mmol), were reacted as described in 1 mL of dry dioxane. The mixture was purified by column chromatography with 40% EtOAc/hexane (1:4) (R_f = 0.2). The desired product **9** was obtained as a deep purple liquid in a yield of 60 mg (37%). ^{1}H NMR (400 MHz, $CDCl_3$) δ 8.71 (d, 4H), 7.19 (t, 2H), 2.08 (s, 12H) ppm. ^{13}C NMR (101 MHz, $CDCl_3$) δ 173.98, 173.84, 156.88, 118.83, 50.38, 27.13 ppm. ESI-HRMS M/z [M+H$^+$] calculated for $C_{16}H1_9N_8$ is 323.173 Da and found 323.172.

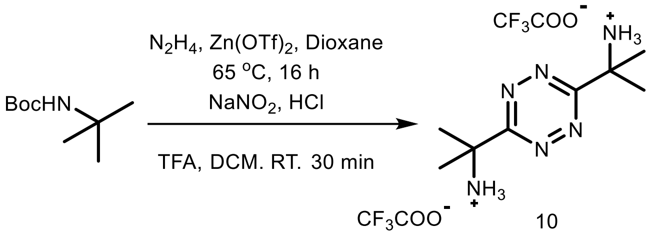

3,6-bis(2-ammoniumprop-2-yl)-1,2,4,5-tetrazine trifluoroacetate **(10):** Synthesized following the general procedure A. 1,1-Dimethylethyl *N*-(1-cyano-1-methylethyl)carbamate (92 mg, 0.5 mmol), anhydrous N_2H_4 (125 μL, 2.5 mmol), and $Zn(OTf)_2$ (9 mg, 0.05 mmol), were reacted in 1 mL of dry dioxane. The mixture was purified by column chromatography using EtOAc/hexanes (1:4) (R_f = 0.3) as the eluent. The Boc-protected intermediate was obtained as a purple solid with quantitative yield. The solid product was redissolved in 5 mL DCM and then 0.5 mL of trifluoroacetic acid (TFA) was added and the mixture stirred for 1 hour at room temperature to deprotect the Boc group. After the completion of the reaction, volatiles were evaporated under reduced pressure. The crude product was washed with diethyl ether several times to obtain the desired compound **10** as a purple solid in a yield of 50 mg (48%). ^{1}H NMR (400 MHz, DMSO-d_6) δ 9.02 (s, 6H), 1.84 (s, 12H). ^{13}C NMR (101 MHz, DMSO-D_6) δ 176.12, 56.60, 26.36 ppm. ESI-HRMS M/z [M+H$^+$] calculated for $C_8H_{17}N_6$ is 197.151 Da and found 197.150.

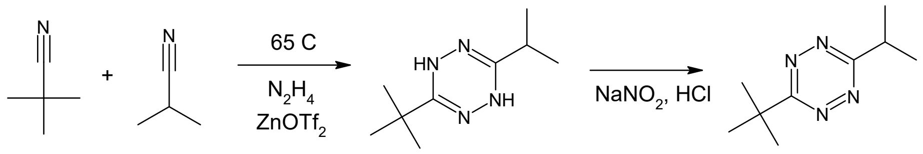

3-tert-butyl-6-(prop-2-yl)-1,2,4,5-tetrazine: Synthesized according to general procedure A. To an oven-dried round bottom flask (250 mL) was added pivalonitrile (3.32 mL, 2.49 g, 30 mmol), isobutyronitrile (1.35 mL, 1.04 g, 15 mmol), and zinc triflate (0.4 g, 1.1 mmol). The mixture was placed under a nitrogen atmosphere, and anhydrous hydrazine (3.5 mL, 3.6 g, 112.5 mmol) was added dropwise. The reaction mixture was stirred at 65 °C for 24 h. Upon completion, the mixture was diluted with DCM and cooled in an ice bath. Aqueous sodium nitrite (17.6 g, 255 mmol) in water (100 mL) was added. Concentrated aqueous HCl was added dropwise until gas formation ceased and the solution reached pH ~3, developing a distinct red/pink color. The organic layer was separated, and the aqueous layer was extracted twice with DCM. Combined organic extracts were washed with water, dried over $MgSO_4$, filtered, and evaporated. The residue was dissolved in DCM and purified by column chromatography (Hexanes/DCM 1:1). Product-containing fractions were combined and evaporated to yield the product as a purple solid in a yield of 300 mg (11%).

^{1}H NMR (500 MHz, $CDCl_3$) δ 3.64 (hept, 1H, J = 7.0 Hz), 1.60 (s, 9H), 1.57 (d, 6H, J = 7.0 Hz). ^{13}C NMR (125 MHz, $CDCl_3$) δ 176.04, 173.50, 38.25, 34.58, 29.61, 21.68 ppm.

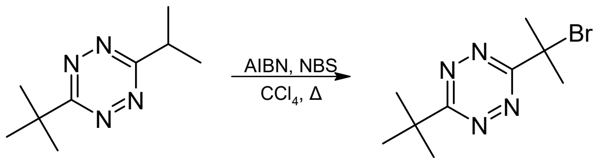

3-(2-bromoprop-2-yl)-6-*tert*-butyl-1,2,4,5-tetrazine (13): 3-*tert*-butyl-6-(prop-2-yl)-1,2,4,5-tetrazine (75 mg, 0.42 mmol) was dissolved in carbon tetrachloride (4 mL). Azobisisobutyronitrile (20 mg, 0.125 mmol; explosive!) and *N*-bromosuccinimide (NBS; 93 mg, 0.525 mmol) were added. The mixture was refluxed under nitrogen for 6 h. The solution, containing a white precipitate, was directly loaded onto a silica gel column and purified by flash chromatography (Hexanes/DCM, 3:2). Product-containing fractions were combined and evaporated to yield the product as a purple solid in a yield of 62 mg (46%).

^{1}H NMR (500 MHz, $CDCl_3$) δ 2.44 (s, 6H), 1.61 (s, 9H). ^{13}C NMR (125 MHz, $CDCl_3$) δ 175.93, 171.83, 58.37, 38.54, 32.68, 29.60 ppm.

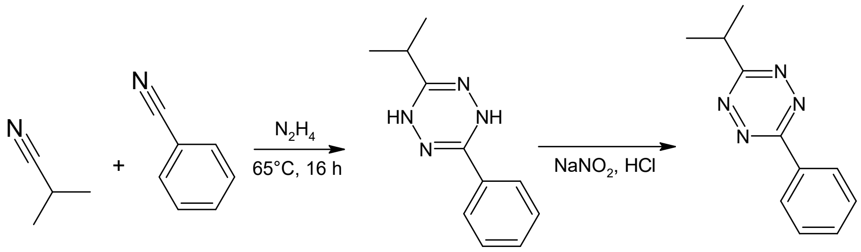

3-phenyl-6-(prop-2-yl)-1,2,4,5-tetrazine: Synthesized according to general procedure A. To an oven-dried round bottom flask (10 mL) was added benzonitrile (309 µL, 309 mg, 3 mmol), isobutyronitrile (539 µL, 415 mg, 6 mmol), and zinc triflate (109 mg, 0.3 mmol). The mixture was placed under a nitrogen atmosphere, and anhydrous hydrazine (471 µL, 480 mg, 15 mmol) was added dropwise. The reaction mixture was stirred at 65 °C for 16 h. Upon completion, the mixture had solidified. It was dispersed in DCM (20 mL), transferred to a larger round-bottom flask, and cooled in an ice bath. Aqueous sodium nitrite (4.1 g, 60 mmol) in water (50 mL) was added. Concentrated aqueous HCl was added dropwise until gas formation ceased and the solution reached pH ~3, developing a distinct pink/purple color. The organic layer was separated, and the aqueous layer was extracted with DCM. Combined organic extracts were dried over $MgSO_4$, filtered, and evaporated. The residue was purified by column chromatography (Hexanes/DCM 2:3). Product-containing fractions were combined and evaporated to yield the product as a purple solid in a yield of 170 mg (28%).

^{1}H NMR (500 MHz, $CDCl_3$) δ 8.60 (d, 2H, J = 7.0 Hz), 7.56-7.62 (m, 3H), 3.69 (hept, 1H, 6.75 Hz), 1.57 (d, 6H, J = 7.0 Hz). ^{13}C NMR (125 MHz, $CDCl_3$) δ 174.09, 164.71, 132.92, 132.38, 129.70, 128.38, 34.68, 21.72 ppm.

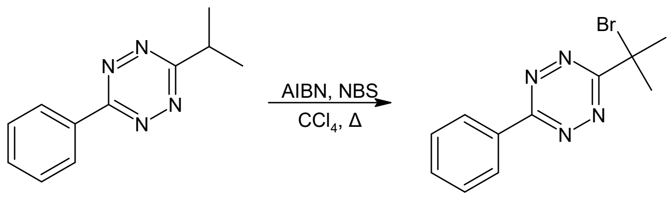

3-(2-bromoprop-2-yl)-6-phenyl-1,2,4,5-tetrazine (14): 3-phenyl-6-(prop-2-yl)-1,2,4,5-tetrazine (105 mg, 0.52 mmol) was dissolved in carbon tetrachloride (5 mL). Azobisisobutyronitrile (26 mg, 0.16 mmol; explosive!) and *N*-bromosuccinimide (117 mg, 0.66 mmol) were added. The mixture was refluxed and monitored by TLC. After 6 h, the reaction had reached completion, and the mixture was cooled to room temperature. The solution, containing a white precipitate, was directly loaded onto a silica gel column and purified by flash chromatography (Hexanes/DCM, 1:1). Product-containing fractions were combined and evaporated to yield the product as a pink solid in a yield of 84 mg (58%).

^{1}H NMR (500 MHz, $CDCl_3$) δ 8.63 (d, 2H, J = 7.5), 7.55-7.75 (m, 3H), 2.48 (s, 6H) ppm. ^{13}C NMR (101 MHz, $CDCl_3$) δ 171.89, 163.62, 133.16, 131.44, 129.46, 128.43, 58.07, 32.29 ppm.

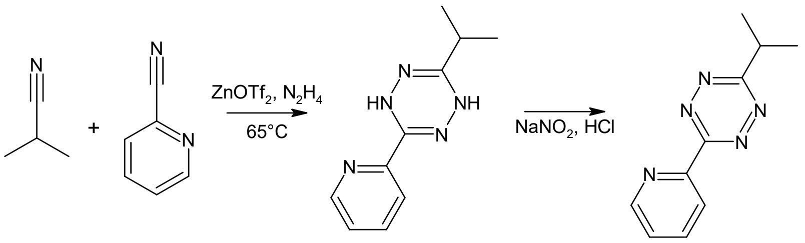

3-(prop-2-yl)-6-(pyrid-2-yl)-1,2,4,5-tetrazine: Synthesized according to general procedure A. To an oven-dried round bottom flask (10 mL) was added 2-pyridinecarbonitrile (312 mg, 3 mmol), isobutyronitrile (539 µL, 415 mg, 6 mmol), and zinc triflate (109 mg, 0.3 mmol). The mixture was placed under a nitrogen atmosphere, and anhydrous hydrazine (471 µL, 480 mg, 15 mmol) was added dropwise. The reaction mixture was stirred at 65 °C for 16 h. Upon completion, the mixture had solidified. It was dispersed in DCM (20 mL), transferred to a larger round-bottom flask, and cooled in an ice bath. Aqueous sodium nitrite (4.1 g, 60 mmol) in water (50 mL) was added. Concentrated aqueous HCl was added dropwise until gas formation ceased and the solution reached pH ~3, developing a distinct pink/purple color. The organic layer was separated, and the aqueous layer was extracted with DCM. Combined organic extracts were dried over $MgSO_4$, filtered, and evaporated. The residue was purified by column chromatography (DCM + 2% MeOH). Product-containing fractions were combined and evaporated to yield the product as a deep purple liquid in a yield of 160 mg (27%).

^{1}H NMR (500 MHz, $CDCl_3$) δ 8.95 (d, 1H, J = 4.5 Hz), 8.64 (d, 1H, J = 8.0 Hz) 7.99 (dd, 1H), 7.56 (dd, 1H, J_1 = 7.0 Hz, J_2 = 5.0 Hz), 3.77 (hept, 1H, J = 7.0 Hz), 1.59 (d, 6H, J = 7.0 Hz). ^{13}C NMR (125 MHz, $CDCl_3$) δ 174.86, 164.23, 151.27, 150.91, 137.86, 126.67, 124.27, 34.83, 21.71 ppm.

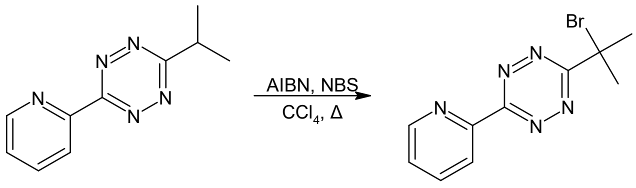

3-(2-bromoprop-2-yl)-6-(pyrid-2-yl)-1,2,4,5-tetrazine (15): 3-(prop-2-yl)-6-(pyrid-2-yl)-1,2,4,5-tetrazine (110 mg, 0.52 mmol) was dissolved in carbon tetrachloride (5 mL). Azobisisobutyronitrile (27 mg, 0.16 mmol; explosive!) and *N*-bromosuccinimide (122 mg, 0.68 mmol) were added. The mixture was refluxed under nitrogen. After 6 h, the reaction had reached completion. The mixture was purified by silica gel column chromatography (DCM + 0.5% MeOH). Product-containing fractions were combined and evaporated to yield the product in a yield of 142 mg (86%).

^{1}H NMR (500 MHz, $CDCl_3$) δ 8.97 (d, 1H, J = 4.5 Hz), 8.69 (d,1H, J = 8.0Hz), 8.01 (ddt, 1H, J_1 = J_2 = 7.75 Hz, J_3 = 1.0 Hz), 7.59 (dd, 1H, J_1 = 7.5 Hz, J_2 = 5.0 Hz), 2.50 (s, 6H) ppm. ^{13}C NMR (125 MHz, $CDCl_3$) δ 172.81, 163.38, 151.47, 150.37, 127.05, 124.79, 58.16, 32.54 ppm.

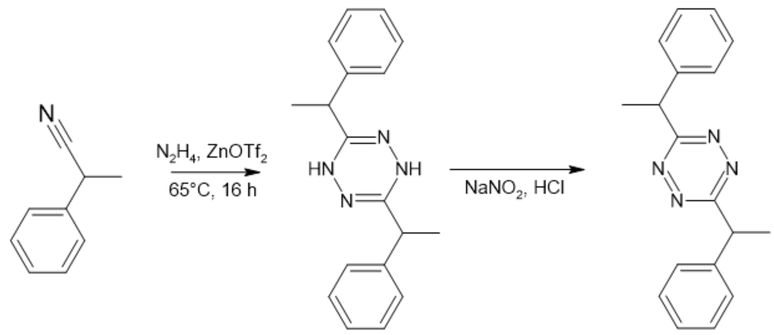

3,6-bis(1-phenylethyl)-1,2,4,5-tetrazine. Synthesized according to general procedure A. To an oven-dried round bottom flask (25 mL) was added 2-phenylpropionitrile (1.33 mL, 1.31 g, 10 mmol) and zinc triflate (182 mg, 0.5 mmol). The mixture was placed under a nitrogen atmosphere, and anhydrous hydrazine (1.57 mL, 1.60 g, 50 mmol) was added dropwise. The reaction mixture was stirred at 65 °C for 16 h. The mixture was diluted with DCM (20 mL), transferred to a larger round-bottom flask, and cooled in an ice bath. Aqueous sodium nitrite (6.9 g, 100 mmol) in water (75 mL) was added. Concentrated aqueous HCl was added dropwise until gas formation ceased and the solution reached pH ~3. The organic layer was separated, and the aqueous layer was extracted with DCM. Combined organic extracts were washed with brine, dried over $MgSO_4$, filtered, and evaporated. The residue was purified by column chromatography (Hexanes/DCM 1:2). Product-containing fractions were combined and evaporated to yield the product as a purple liquid in a yield of 743 mg (26%).

^{1}H NMR (500 MHz, $CDCl_3$) δ 7.23 (d, 2H, J = 7.5 Hz), 7.31 (dd, 2H, J_1 = J_2 = 7.5 Hz), 7.23 (d, 1H, J = 7.5 Hz), 4.79 (q, 2H, J = 7.0 Hz), 1.89 (d, 6H, J = 7.5). ^{13}C NMR (101 MHz, $CDCl_3$) δ 172.50, 142.16, 129.29, 128.33, 127.80, 45.80, 20.446, 20.415 (diastereomers) ppm.

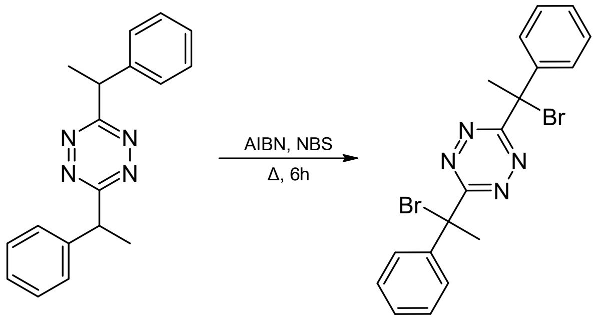

3,6-bis(1-bromo-1-phenylethyl)-1,2,4,5-tetrazine (16): 3,6-bis-(1-phenylethyl)-1,2,4,5-tetrazine (145 mg, 0.5 mmol) was dissolved in carbon tetrachloride (5 mL) in a 50 mL two-neck round-bottom flask. Azobisisobutyronitrile (46 mg, 0.3 mmol) and *N*-bromosuccinimide (222 mg, 1.25 mmol) were added. The mixture was refluxed under nitrogen for 24 h. The mixture was directly loaded onto a silica gel column and purified by silica column chromatography (Hexanes/DCM, 4:1). Product-containing fractions were combined and evaporated to yield the product as a purple solid in a yield of 40 mg (18%). The *mono*-brominated side product was isolated in a yield of 35 mg (19%).

^{1}H NMR (500 MHz, $CDCl_3$) δ 7.65 (d, 4H, J = 8.0 Hz), 7.30-7.40 (m, 6H), 2.80 (s, 6H) ppm. ^{13}C NMR (101 MHz, $CDCl_3$) δ 171.95, 142.21, 129.03, 128.23, 128.21, 63.36, 32.80 ppm.

Photo-spectrometric Analysis of Reaction Kinetics

Determination of Bimolecular rate constants

DMSO: H_2O = 80:20 (v/v) at T = 25°C:

The bimolecular rate constant was measured in a solvent mixture of DMSO: H_2O = 80:20 (v/v) in 96-well plates at T = 25°C. The tetrazines were incubated at T = 25°C for 10 minutes to equilibrate the temperature, followed by adding the corresponding dienophile. The reaction kinetics were monitored by measuring the disappearance of tetrazines (c = 0.3 mM) color at λ_{abs} = 525 nm in DMSO: H_2O = 80:20 (v/v) at T = 25°C against different dienophiles (c = 1.5-3 mM) for 120 minutes to 16 hours, depending on the rate of reaction.

DMSO: H_2O = 20:80 (v/v) at T = 37°C:

In a 96-well plate, the dienophiles were incubated at T = 37 °C for 5 minutes to equilibrate the temperature. To that prewarmed solution, tetrazine was added and vigorously shaken to initiate the reaction. The reactions of tetrazines (c = 0.3 mM) with dienophile (c = 1.5 mM) in DMSO: H_2O = 20:80 (v/v) at T = 37 °C were monitored by the disappearance of tetrazine at λ_{abs} 525 nm.

Experimentally obtained rate constants were determined by fitting the time-dependent tetrazine absorbance signal to a mono-exponential decay equation. All pseudo-first-order reaction kinetic experiments were performed in triplicate. The rate constants represent the meaning of the three individual rate measurements, and the error is provided as the standard error.

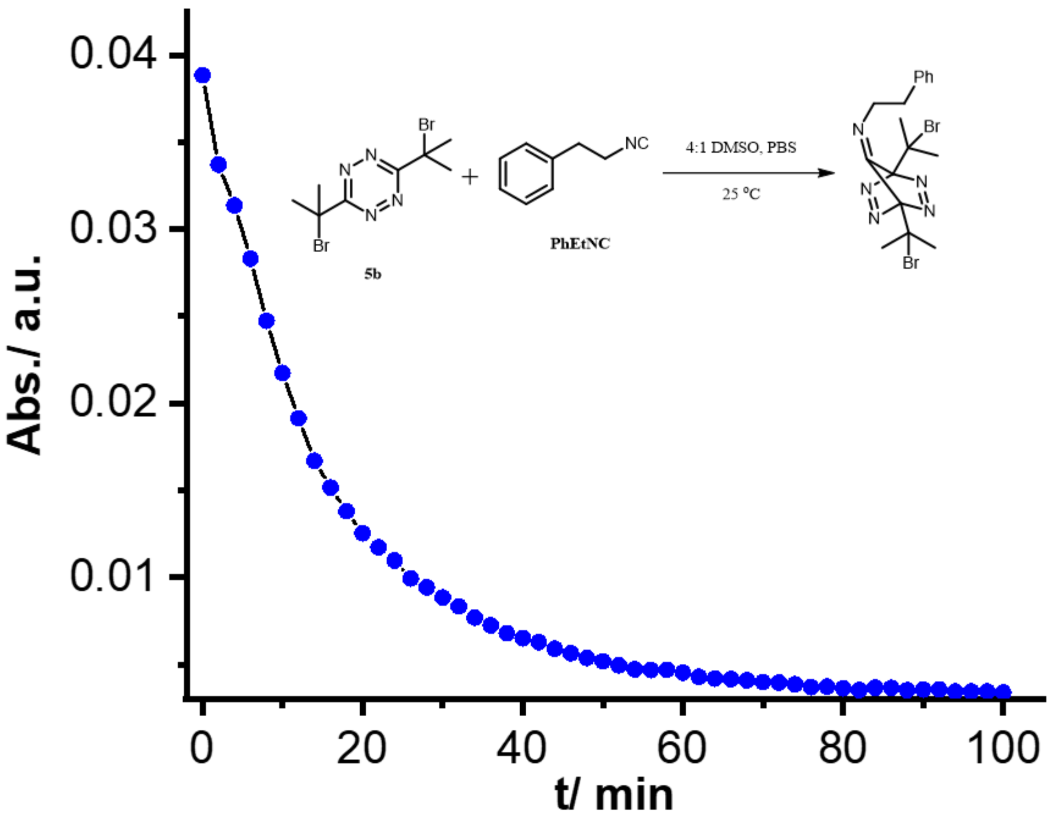

Figure S1: Representative example of measuring bimolecular rate through disappearance of tetrazine absorbance. Reaction kinetics of 3,6-bis(2-bromopropan-2-yl)-1,2,4,5-tetrazine (c = 0.3 mM) against PhEtNC (c = 1.5 mM) an exponential decay in DMSO: H_2O = 80:20 (v/v) at T = 25 ˚C monitored at λ_{abs} 525 nm.

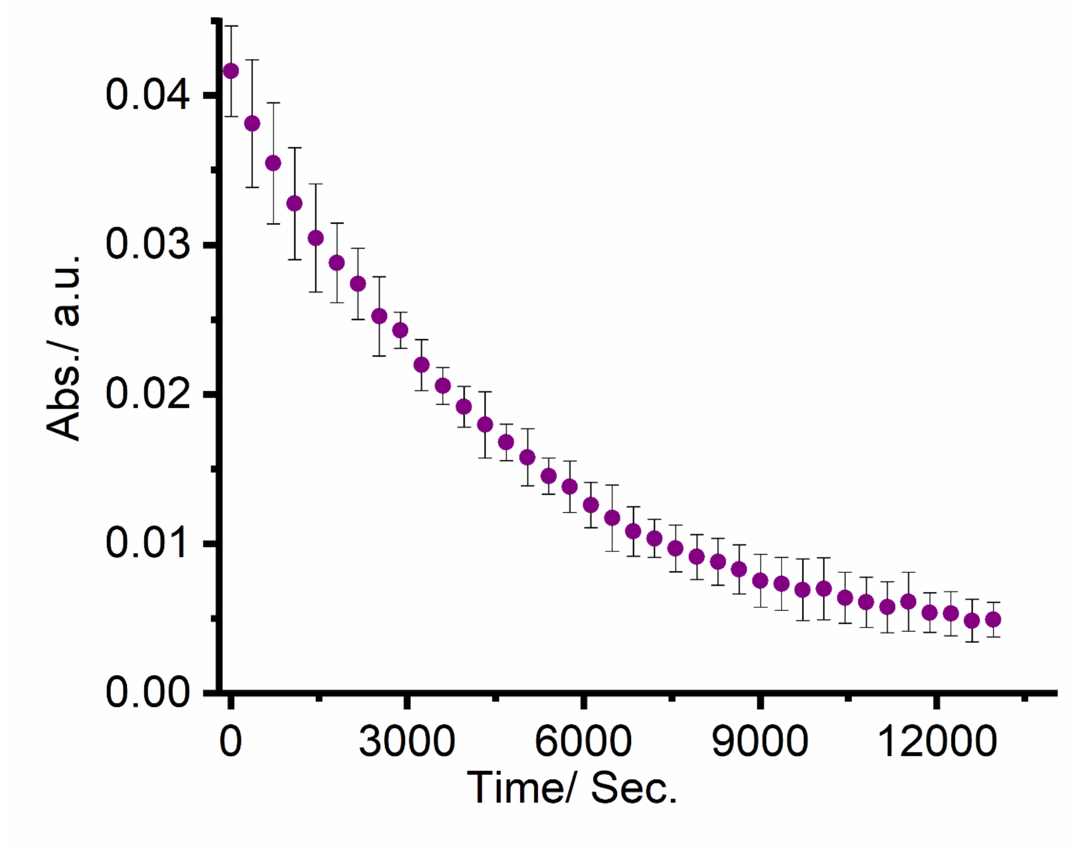

Figure S2: Stability of **5b** in high water buffer. A plot of the absorption of **5b** (0.3 mM) against time in 4:1 (PBS-DMSO), pH 7.4, at 25 °C, showing its decomposition kinetics. The pseudo-first-order decomposition of **5b** exhibits a rate constant of $k_1 = 3.0 \times 10^{-4}\ s^{-1}$.

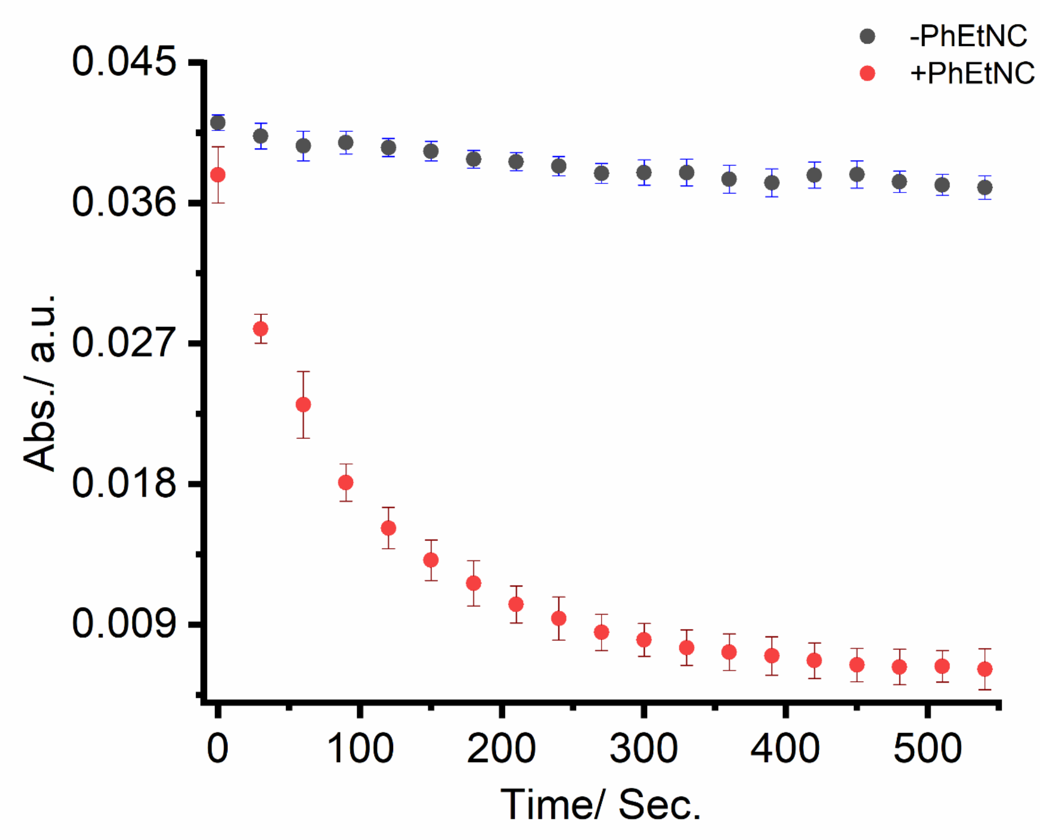

Figure S3: Representative example of measuring bimolecular rate constants through disappearance of tetrazine absorbance. Reaction kinetics of 3,6-bis(2-bromopropan-2-yl)-1,2,4,5-tetrazine (5b, c = 0.3 mM) with and without addition of PhEtNC (1.5 mM) an exponential decay in DMSO: PBS = 20:80 (v/v) at T = 25 ˚C monitored at λ_{abs} 525 nm. The experimentally obtained bimolecular rate constant is 7.15 ± 0.20 $M^{-1}S^{-1}$.

Table S1: Bimolecular rate constant (k_2) for the indicated tetrazines reacted with PhEtNC. [PhEtNC] = 1.5-3 mM, [tetrazines] = 0.3 mM, DMSO: PBS = 80:20 (v/v) at T = 25 ˚C

Tetrazine	Structure	k_2 [M^{-1} s^{-1}]
**6a**	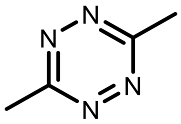	0.006 ± 4×10^{-4}
**7**	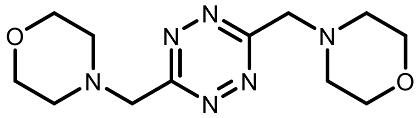	0.043 ± 4×10^{-4}
**2a**	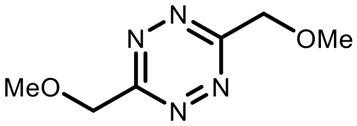	0.036 ± 5×10^{-4}
**4a**	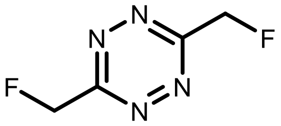	0.520 ± 1.2×10^{-2}
**5a**	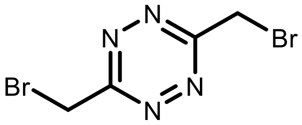	0.025 ± 1×10^{-3}

Table S2: Bimolecular rate constant (k_2) for the indicated tetrazines reacted with PhEtNC. [PhEtNC] = 1.5-3 mM, [substituted dimethyl tetrazines] = 0.3 mM, DMSO: PBS = 80:20 (v/v) at T = 25 ˚C

Tetrazine	Structure	k_2 [$M^{-1}s^{-1}$]
**6b**	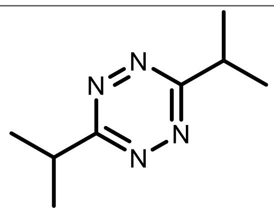	0.008 ± 4×10^{-4}
**8**	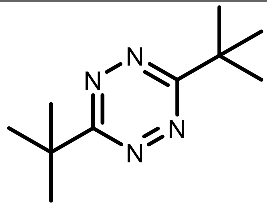	0.06 ± 2×10^{-2}
**10**	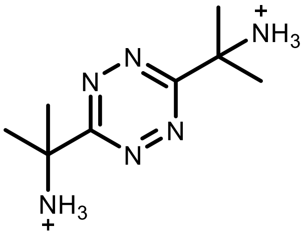	0.052 ± 8×10^{-4}
**2b**	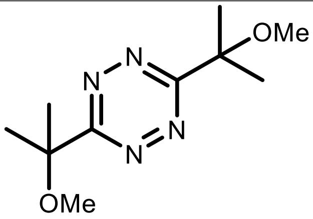	0.053 ± 1×10^{-3}
**4b**	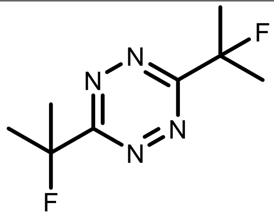	0.980 ± 9.8×10^{-2}
**5b**	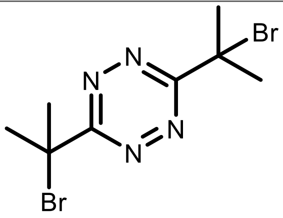	0.490 ± 1.4×10^{-2}
**9**	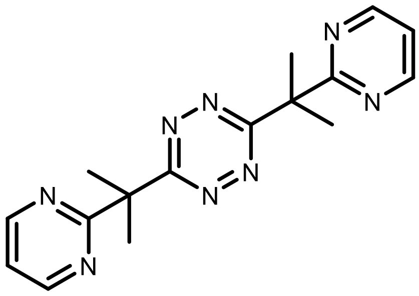	0.032 ± 5.1×10^{-3}

**13**	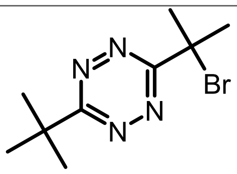	0.061 ± 0.011
**14**	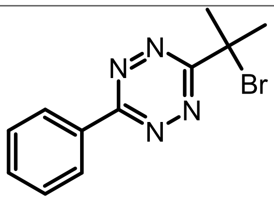	0.086 ± 0.004
**15**	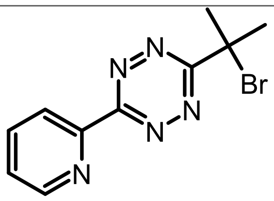	0.54 ± 0.01
**16**	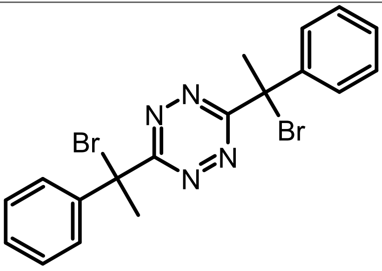	1.7 ± 0.1

Photo-spectrometric stability measurement of the tetrazines

In 96-well plates, tetrazines (0.3 mM) in DMSO: PBS pH 7.4 = 80:20 (v/v), were incubated at T = 25 °C for 10 minutes to equilibrate the temperature. The stability of tetrazines (c = 0.3 mM) was monitored by the disappearance of tetrazine at λ_{abs} = 525 nm in one-minute intervals. The absorbance of control wells was subtracted from the absorbance recorded from the tetrazines. All experiments were run in triplicates. The percentage remaining reported in the main text corresponds to the mean of the three individual absorbance measured and the error is provided as the standard error. The percentage remaining was calculated as the fraction of the initial absorbance after background subtraction.

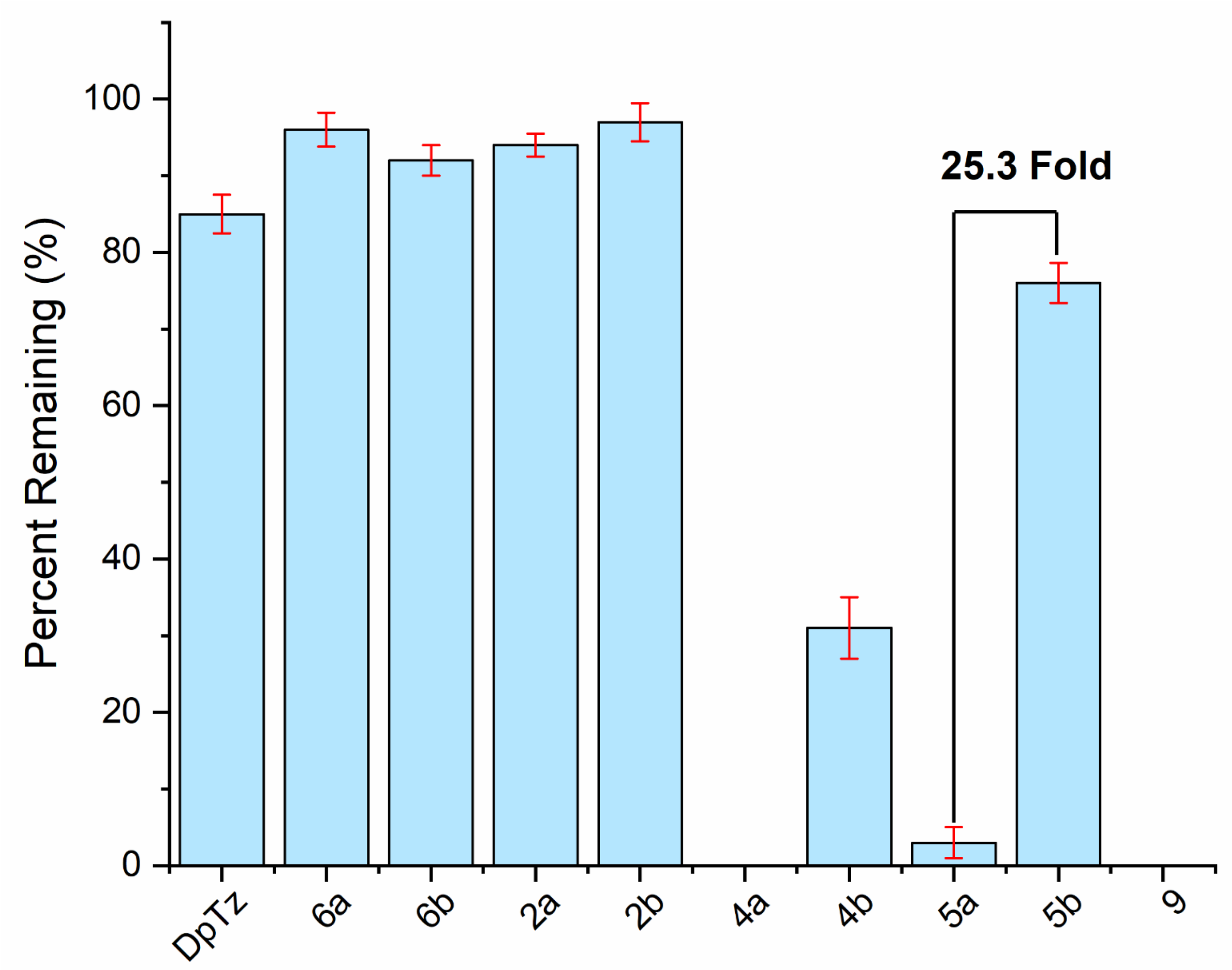

Figure S4: Percent remaining for tetrazines (c = 0.3 mM) displaying decay in DMSO: PBS = 80:20 (v/v) at T = 25 ˚C monitored at λ_{abs} 525 nm up to 12 hours.

HPLC analysis for stability measurement of 3,6-bis(2-bromopropan-2-yl)-1,2,4,5-tetrazine (5b)

After thoroughly investigating the reactivity stability trade-off **(Figure 2e)** of all the synthesized tetrazines, it was essential to measure the stability of 5b in an aqueous system. We performed an HPLC stability analysis of 5b (30 μM) in DMSO: H_2O (4:1, v/v) and DMSO: PBS (4:1, v/v; pH 7.4), 25°C at 0 min and 24 h. The result indicates that more than 75% of the **5b** is intact over 24 h in aqueous solution.

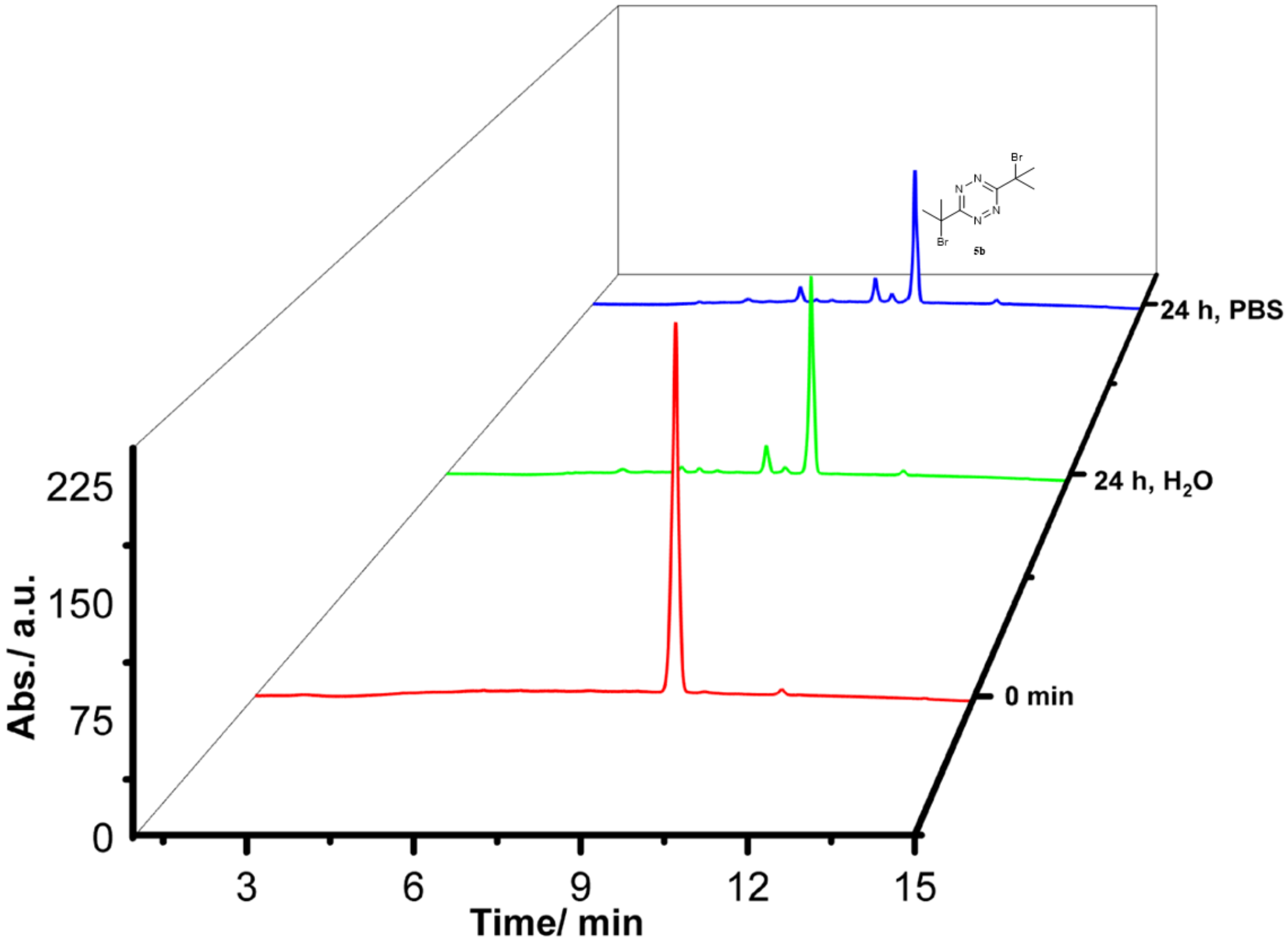

Figure S5: HPLC analysis of the 5b over different periods in DMSO: H_2O (4:1, v/v) and in DMSO: PBS (4:1, v/v) at 25°C.

Computational analysis for the reaction rate of different tetrazine

For the calculation of atomic contributions to intermolecular dispersion interactions we used an extension of the method described by Bistoni and coworkers.[8] Using their python script, together with the DFT-D3 utility by Grimme and coworkers,[2] we first calculated the atomic contributions to dispersion energies of the whole transition state, followed by calculating it for each reactant separately at the transition state geometry. The difference between the atomic dispersion energies in the isolated reactants and the transition state can be attributed to the intramolecular interaction.

Table S3: B3LYP-D3BJ/6-311+G(d,p) CPCM(water) calculated energy of lowest energy conformers. Energies in Hartree.

Compounds	Structures	ΔE	ΔH	ΔG (298 K)
Methyl isocyanide	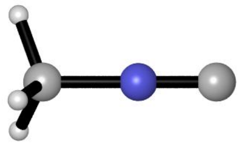	-132.770235	-132.720406	-132.749282
4a 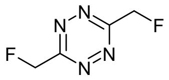	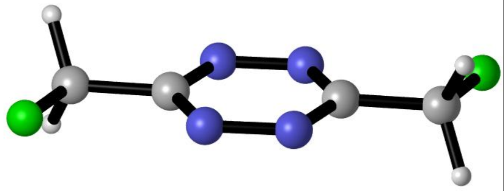	-573.614565	-573.513095	-573.559869
4b 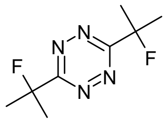	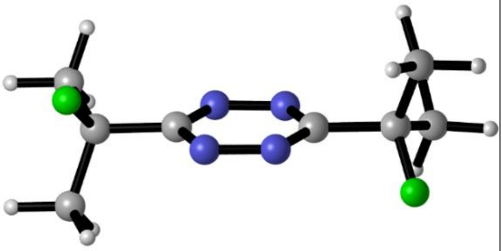	-730.959303	-730.742156	-730.799579
5a 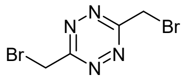	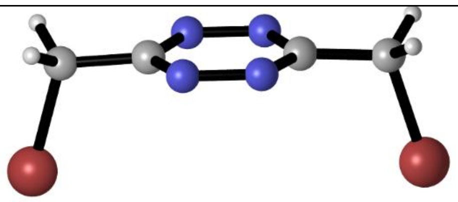	-5522.18625	-5522.08765	-5522.13823
5b 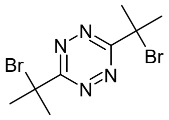	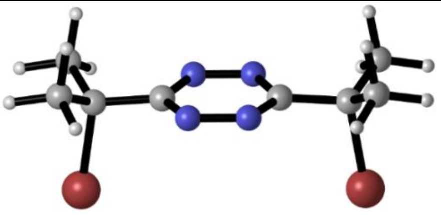	-5679.51682	-5679.30188	-5679.36468

TS_{4a}	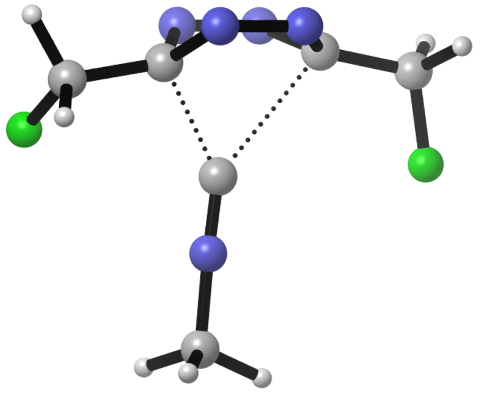	-706.369075	-706.216635	-706.272290
TS_{4b}	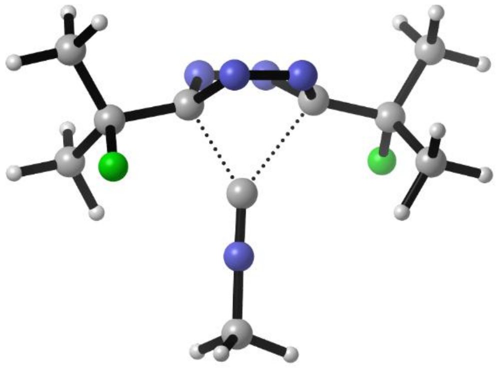	-863.714304	-863.446537	-863.513000
TS_{5a}	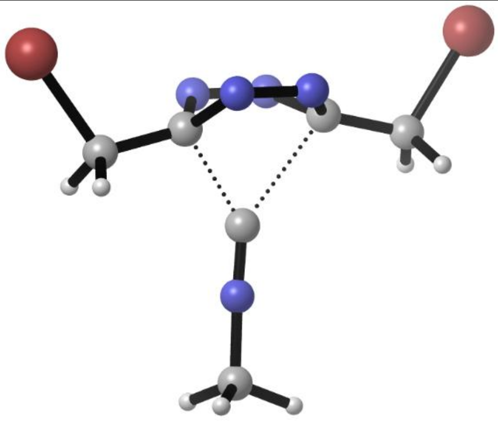	-5654.93709	-5654.78778	-5654.84757
TS_{5b}	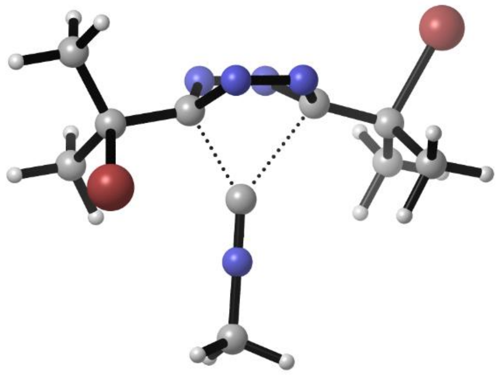	-5812.27088	-5812.00533	-5812.07510

Photospectrometric analysis of tetrazine orthogonality

To test the orthogonality of 5b we have determined the bimolecular rate constant for the reaction of 5b against different dienophiles in DMSO: H_2O; 4:1, pH = 7.4, T=25°C, and compared the values with other tetrazines. The values are provided in Table S4. 5b exhibits a high preference for primary isonitriles with substantial orthogonality towards other dienophiles and tertiary isonitriles. In contrast, the orthogonality of DPTz is low. Where n.d. (the reactions are not completing even over 24 hr.) is not determinable due to slow reaction, and n.r. is no reaction between the couples.

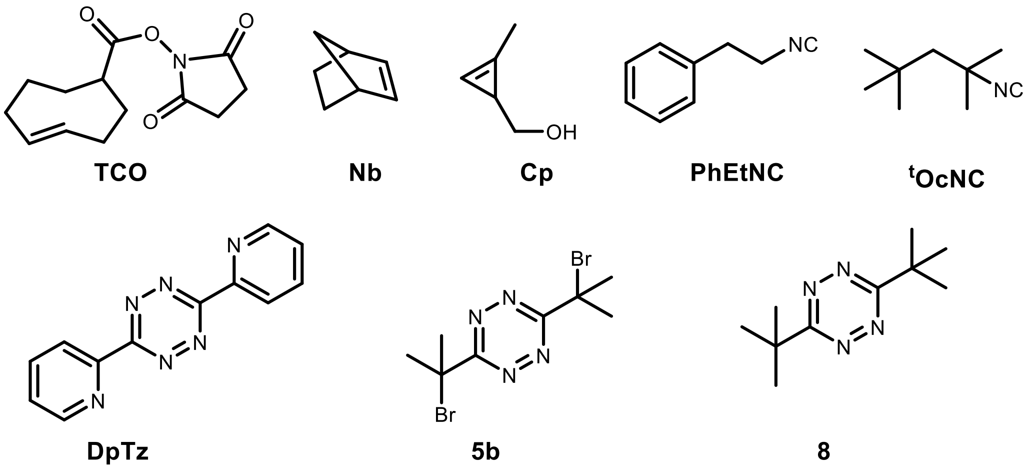

Chart S1: Structures of tested reactants in biorthogonality assay.

Table S4: Bimolecular rate constant (k_2) for the indicated tetrazines (0.3 mM) with various dienophile (1.5-3 mM), in DMSO: PBS = 80:20 (v/v) at T = 25 ˚C

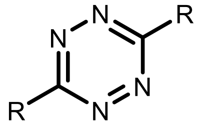 Tetrazine	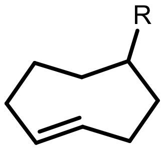 TCO k_2, TCO [L mol^{-1}S^{-1}]	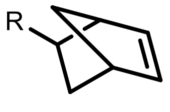 Norbornene (Nb) k_2, Nb [L mol^{-1}S^{-1}]	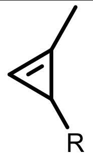 Methyl Cyclopropene (Cp) k_2, Cp [L mol^{-1}S^{-1}]	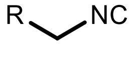 Primary Isonitrile (1˚ NC) k_2, 1˚ NC [L mol^{-1}S^{-1}]	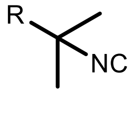 Tertiary Isonitrile (3˚ NC) k_2, 3˚ NC [L mol^{-1}S^{-1}]
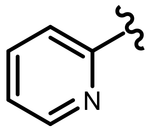	>100	0.25 ± 1.8 × 10^{-2}	0.18± 8× 10^{-3}	0.13± 4× 10^{-3}	0.83± 4.5× 10^{-2}

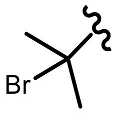	n.d.	n.r.	n.d.	0.49± 2× 10^{-2}	0.018± 3×10^{-3}
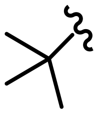	n.d.	n.r.	n.d.	0.077± 2×10^{-2}	n.d.

Effects of 1˚, 2˚, 3˚ Isonitriles on Reactivity against Tetrazine

We investigated the effect of different isonitrile structures on the bimolecular rate constants (k_2) with 5b in a DMSO: H_2O = 80:20 (v/v) mixture at 25 °C. The bar plot shows that primary and secondary isonitriles react similarly with isopropyl bromo-tetrazine, whereas the tertiary isonitrile has a significantly lower rate constant compared to the other two.

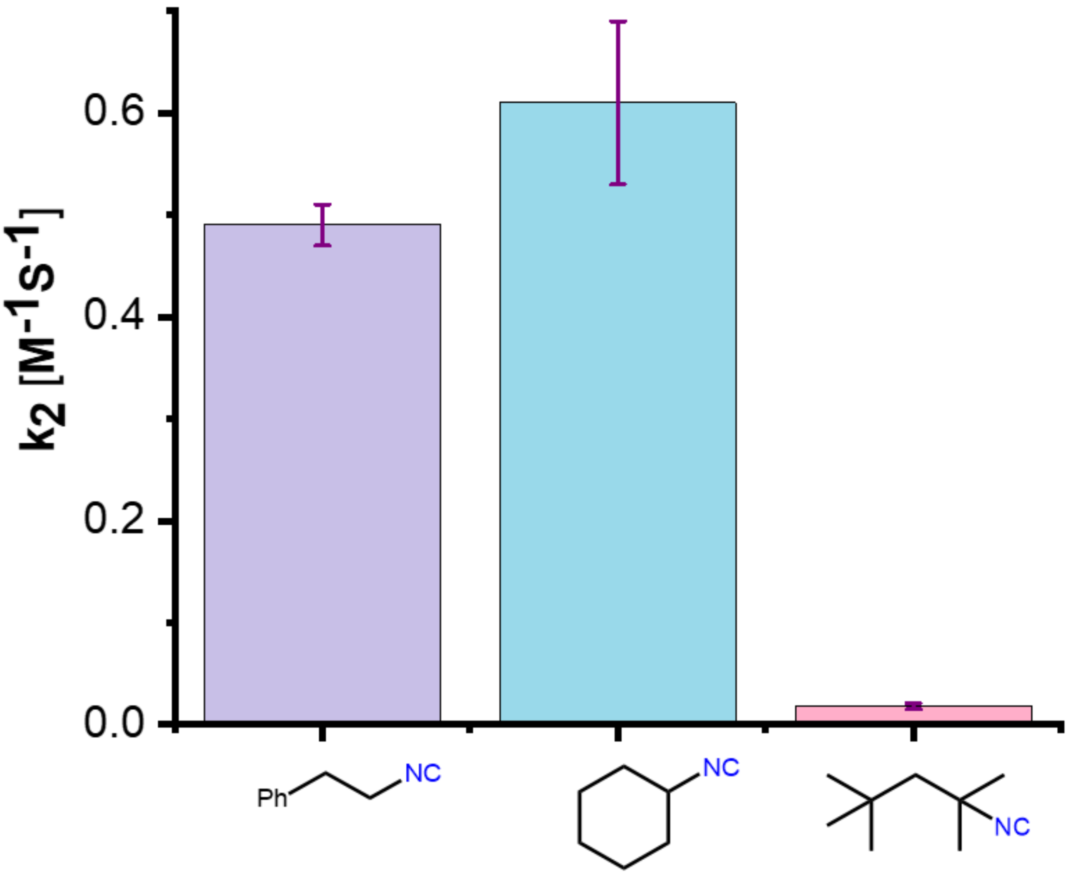

Figure S6: Bi-molecular rate constants (k_2) of the different isonitrile with 5b in DMSO: PBS = 80:20 (v/v), at 25 ˚C.

Photospectrometric Analysis Fluorophore Release

To a preheated solution of N-(methyl-PEG4)-4-(3-isocyanopropyl-1-oxy)-1,8-naphthalimide (ICPr-O-NA, 35 μM) in a DMSO: PBS = 1:1 (v/v) mixture at 37 ˚C, contained in a 3 mL quartz cuvette, 5b (100 μM) was added. The reaction was monitored by absorption spectroscopy.

Photospectrometric Analysis of Resorufin Release

To determine the in vivo applicability of the tetrazines revealed in this study, we performed a fluorescence recovery experiment using caged resorufin. In this experiment, a 1 mL cuvette containing a 10 μM solution of ICPr-res in a 1:1 DMSO-H_2O mixture was treated with 100 μM of 5b at 37 ˚C. The kinetics of the fluorescence turn-on from resorufin was monitored by exciting at 590 nm and measuring the emission at 610 nm.

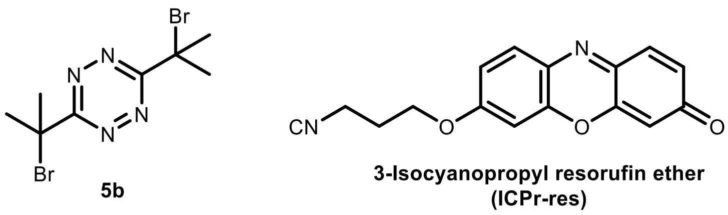

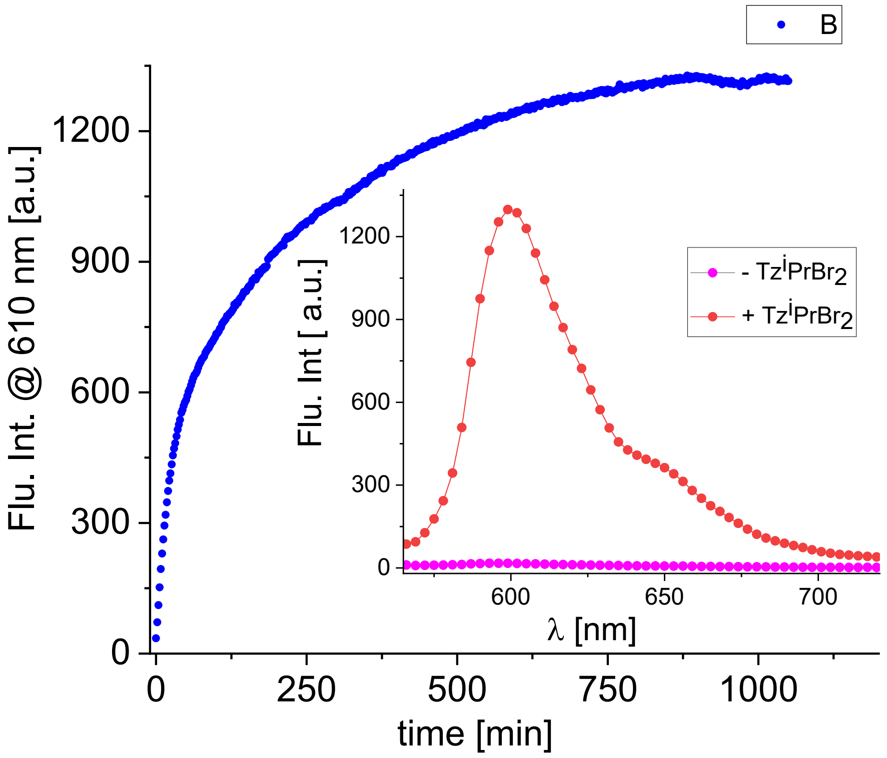

Figure S7: Time-dependent fluorescence recovery kinetics from the decaging of ICPr-res (10 mM) by 5b (100 μ*M*) monitored at 610 nm, in DMSO: PBS = 1:1 (v/v) mixture at 37 ˚C, inset shows the change in emission spectra due to release of free resorufin.

Physiological impact of 3,6-bis(2-bromopropan-2-yl)-1,2,4,5-tetrazine on zebrafish

We carried out a toxicity analysis by injecting three different concentrations (0.35mM, 0.55mM, 0.75mM) of **5b** separately or DMSO as a control and a constant concentration (3.5mM) of Resorufin (ICPr-Rsf) in four larval groups at the 2dpf stage. We then recorded and manually counted the heartbeats of the larvae post 2 and 4 hours of the incubation period. Our results showed a reduced heart rate after the first 2 hours of ICPr-Rsf-TzBr injections in all three concentrations compared to DMSO controls, with a moderate decrement as the concentration of **5b** was increased. Whereas a significant increase in heart rate was observed in post 4 hours of incubation in all treatment groups, and an increase in heart rate response in the 0.35mM treatment group was found to be close to DMSO control larvae after 4 hours. The results show the feasibility of using these compounds in living systems without posing a significant health risk to the subjects due to compound toxicity.

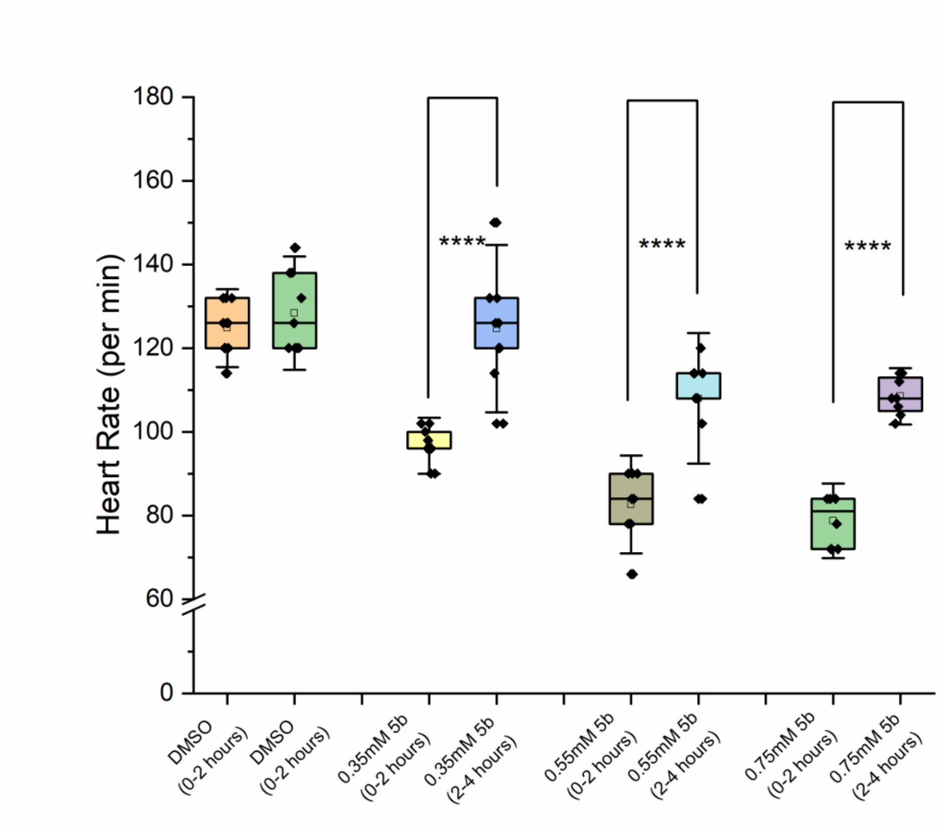

Figure S8: The toxicity of **5b** was measured in Zebrafish during the incubation time with various concentrations and times. Two-way repeated measures ANOVA ($F(5, 21) = 21.10$, $p < 0.01$) and post hoc analysis indicated a significant difference of dose-dependent response of TzBr on heart rate, where post-2-hour incubation showed a decreased heart rate compared to DMSO controls. However, post 4 hour incubation period, heart rate response significantly increased in the treatment groups only ($p < 0.0001$).

References

1. Tu, J.; Svatunek, D.; Parvez, S.; Liu, A. C.; Levandowski, B. J.; Eckvahl, H. J.; Peterson, R. T.; Houk, K. N.; Franzini, R. M., *Angew. Chem. Int. Ed.* **2019,** *58* (27), 9043-9048.
2. Grimme, S.; Antony, J.; Ehrlich, S.; Krieg, H., *J. Chem. Phys.* **2010,** *132* (15), 154104.
3. Bannwarth, C.; Ehlert, S.; Grimme, S., *J. Chem. Theory Comput.* **2019,** *15* (3), 1652-1671.
4. Xu, M.; Deb, T.; Tu, J.; Franzini, R. M., *J. Org. Chem.* **2019,** *84* (23), 15520-15529.
5. Yang, J.; Karver, M. R.; Li, W.; Sahu, S.; Devaraj, N. K., *Angew. Chem. Int. Ed.* **2012,** *51* (21), 5222-5225.
6. Tu, J.; Svatunek, D.; Parvez, S.; Eckvahl, H. J.; Xu, M.; Peterson, R. T.; Houk, K. N.; Franzini, R. M., *Chem. Sci.* **2020,** *11* (1), 169-179.
7. Savastano, M.; Bazzicalupi, C.; Giorgi, C.; García-Gallarín, C.; López de la Torre, M. D.; Pichierri, F.; Bianchi, A.; Melguizo, M., *Inorg. Chem.* **2016,** *55* (16), 8013-8024.
8. Baldinelli, L.; De Angelis, F.; Bistoni, G., *J. Chem. Theory Comput.* **2024,** *20* (5), 1923-1931.

^{1}H-NMR spectra of synthesized compounds

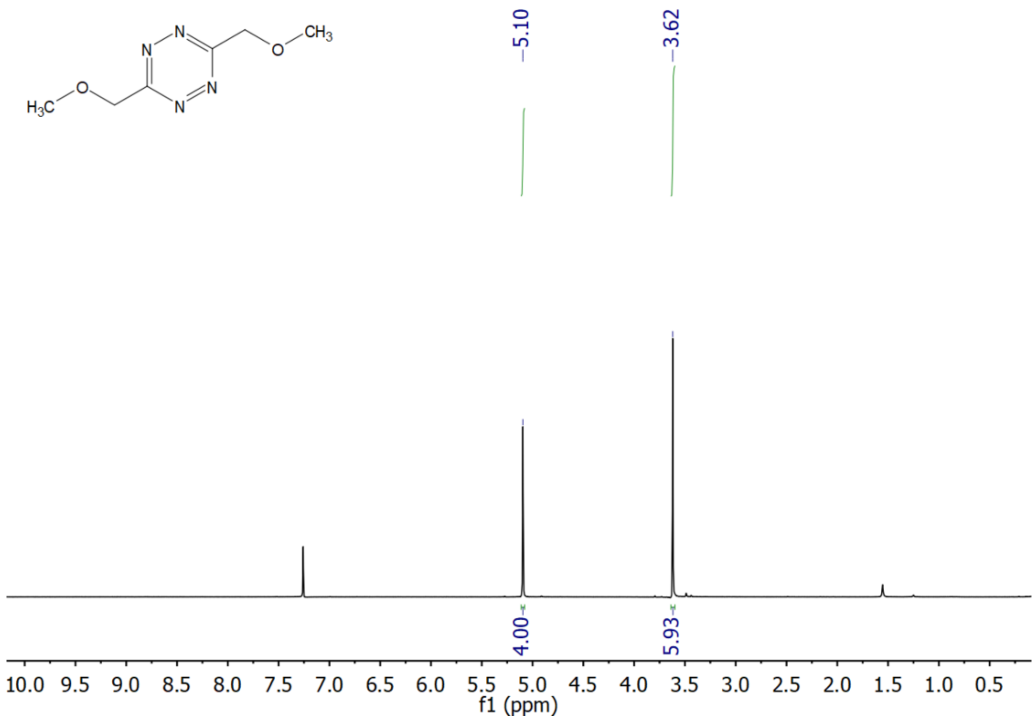

Figure S9: ^{1}H NMR spectrum of *3,6-bis(methoxymethyl)-1,2,4,5-tetrazine* (400 MHz; $CDCl_3$).

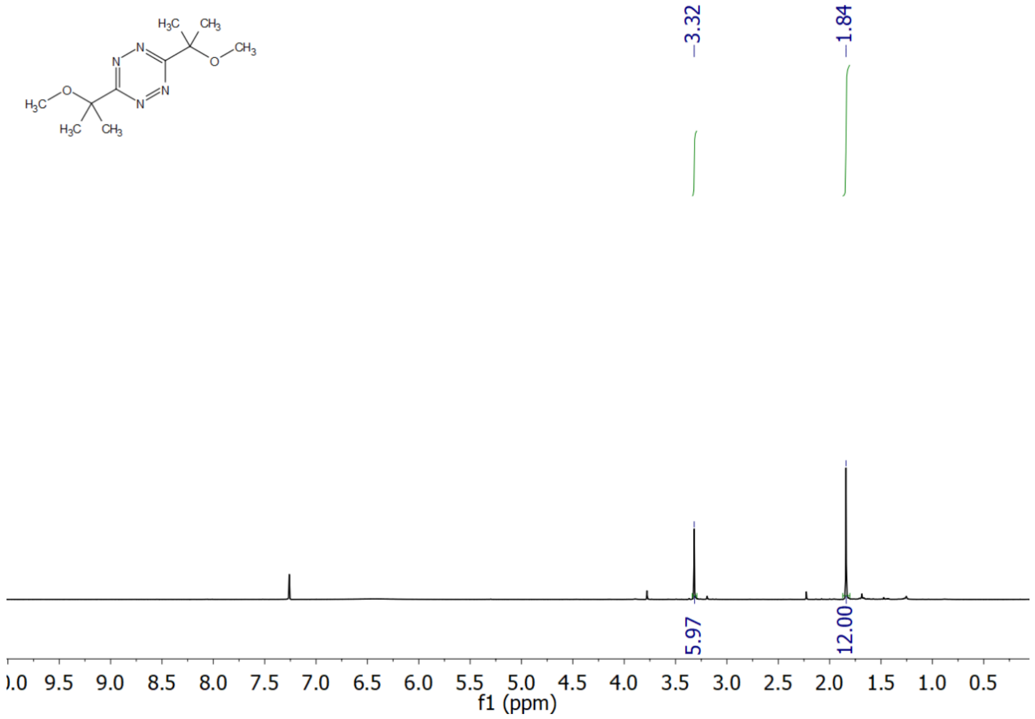

Figure S10: ^{1}H NMR spectrum of 3,6-bis(2-methoxyprop-2-yl)-1,2,4,5-tetrazine (400 MHz; $CDCl_3$).

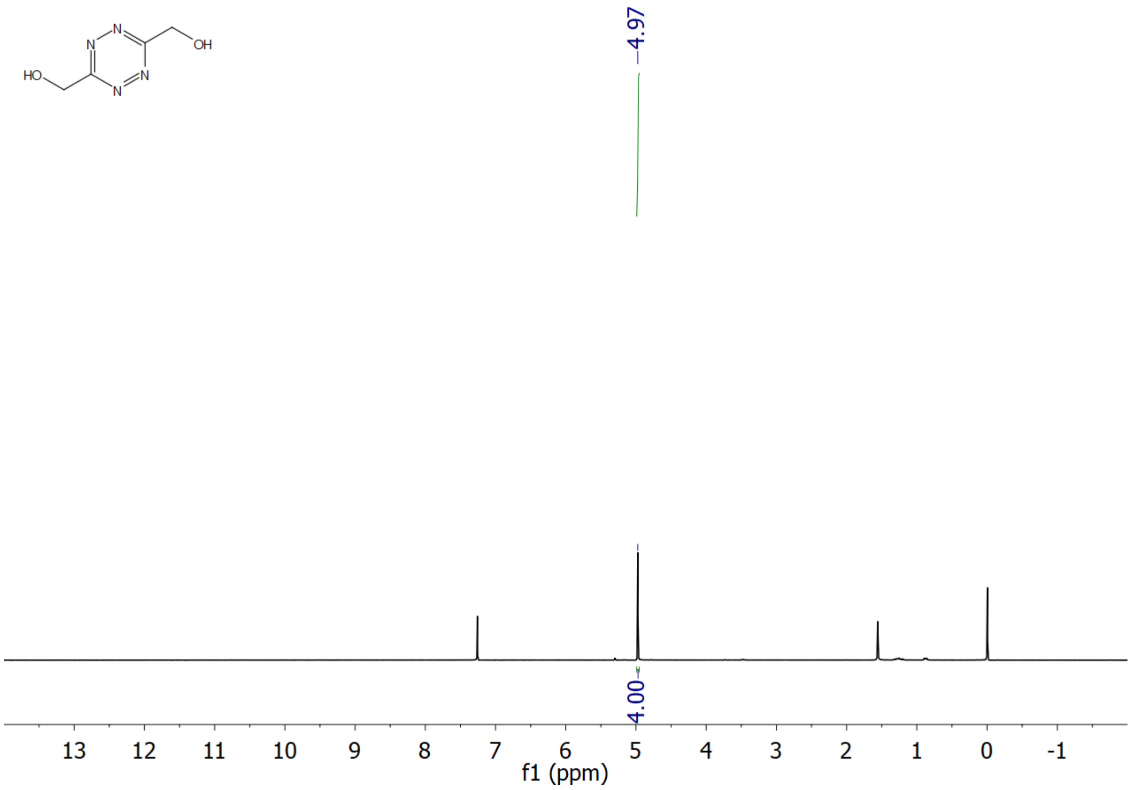

Figure S11: ^{1}H NMR spectrum of *(1,2,4,5-tetrazine-3,6-diyl)dimethanol* (400 MHz; $CDCl_3$).

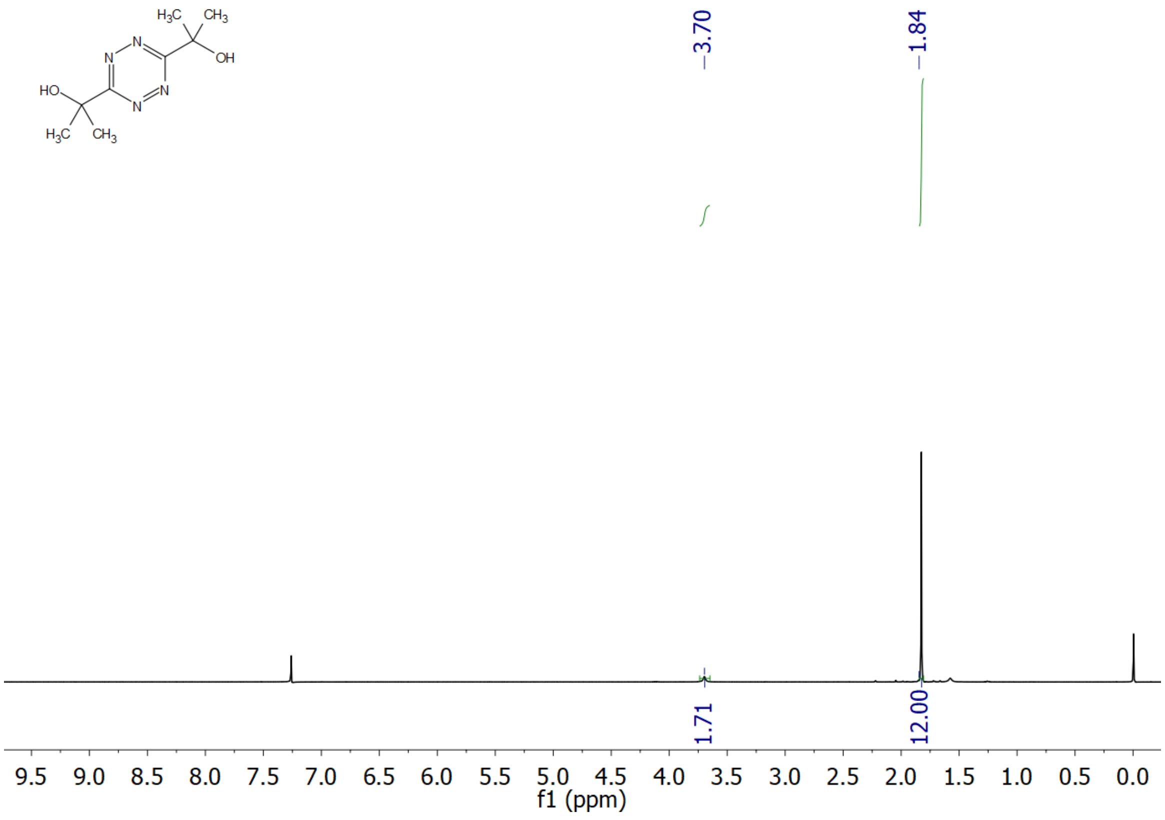

Figure S12: ^{1}H NMR spectrum of *2,2'-(1,2,4,5-tetrazine-3,6-diyl)bis(propane-2-ol)* (400 MHz; $CDCl_3$).

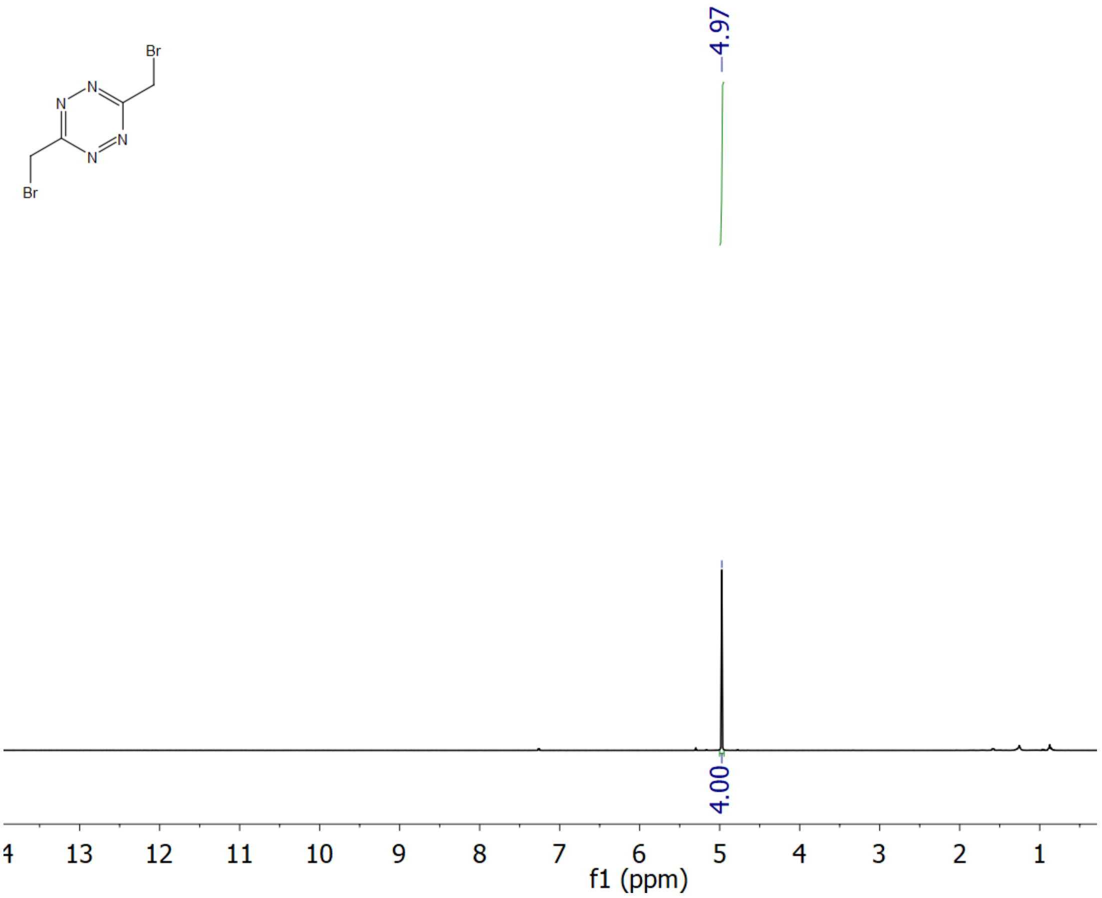

Figure S13: ^{1}H NMR spectrum of *3,6-bis(bromomethyl)-1,2,4,5-tetrazine* (400 MHz; $CDCl_3$).

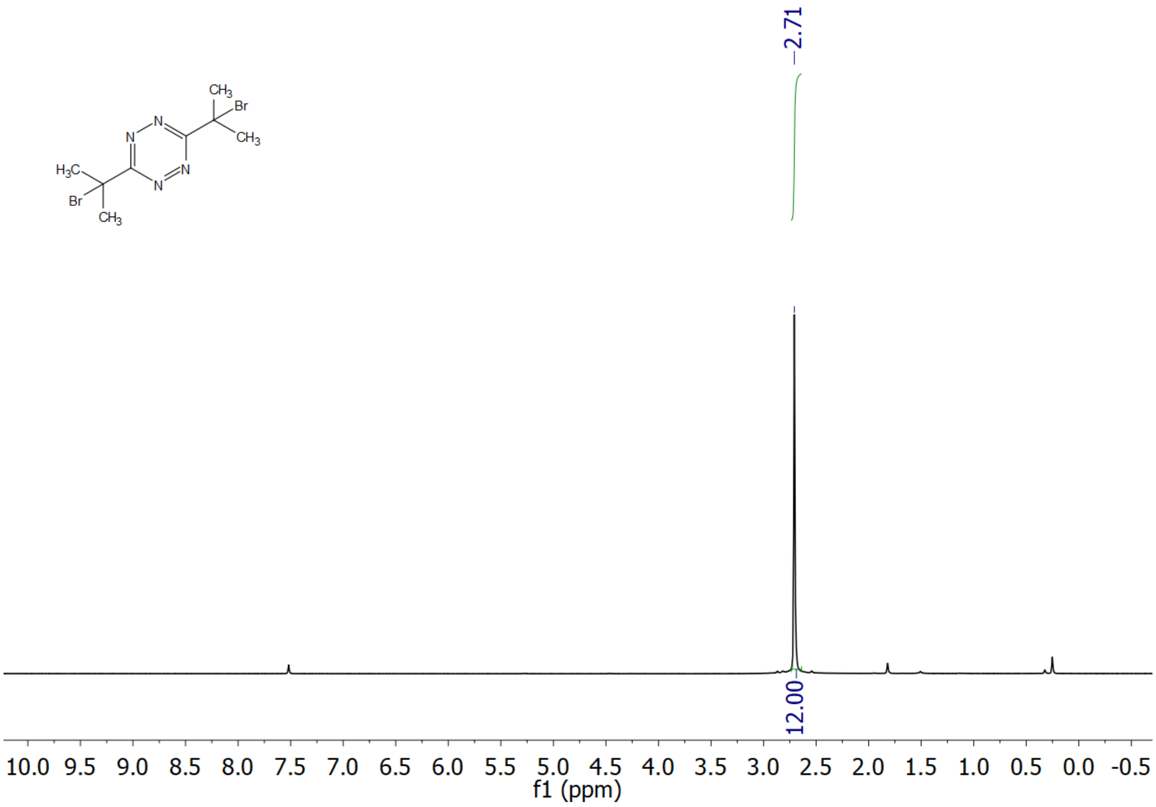

Figure S14: ^{1}H NMR spectrum of *3,6-bis(2-bromopropan-2-yl)-1,2,4,5-tetrazine* (400 MHz; $CDCl_3$).

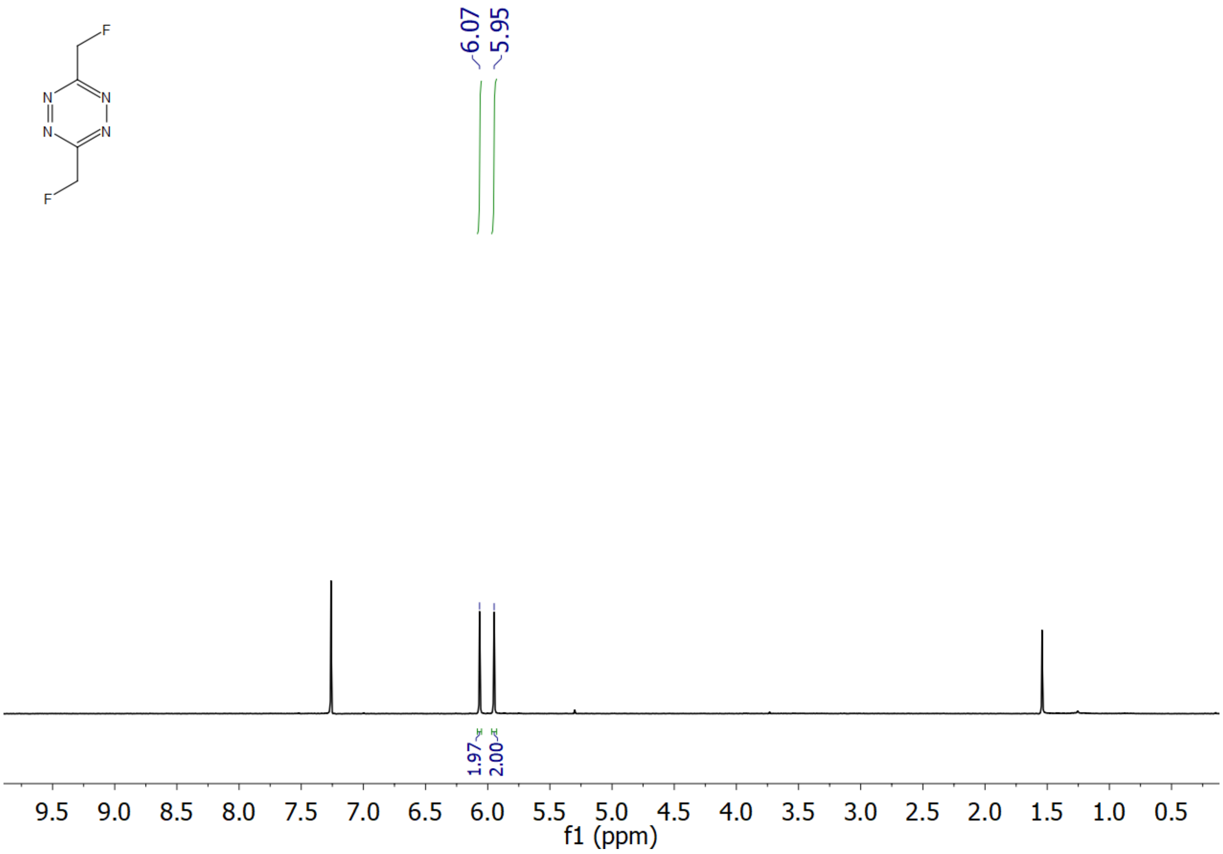

Figure S15: ^{1}H NMR spectrum of *3,6-bis(fluoromethyl)-1,2,4,5-tetrazine* (400 MHz; $CDCl_3$).

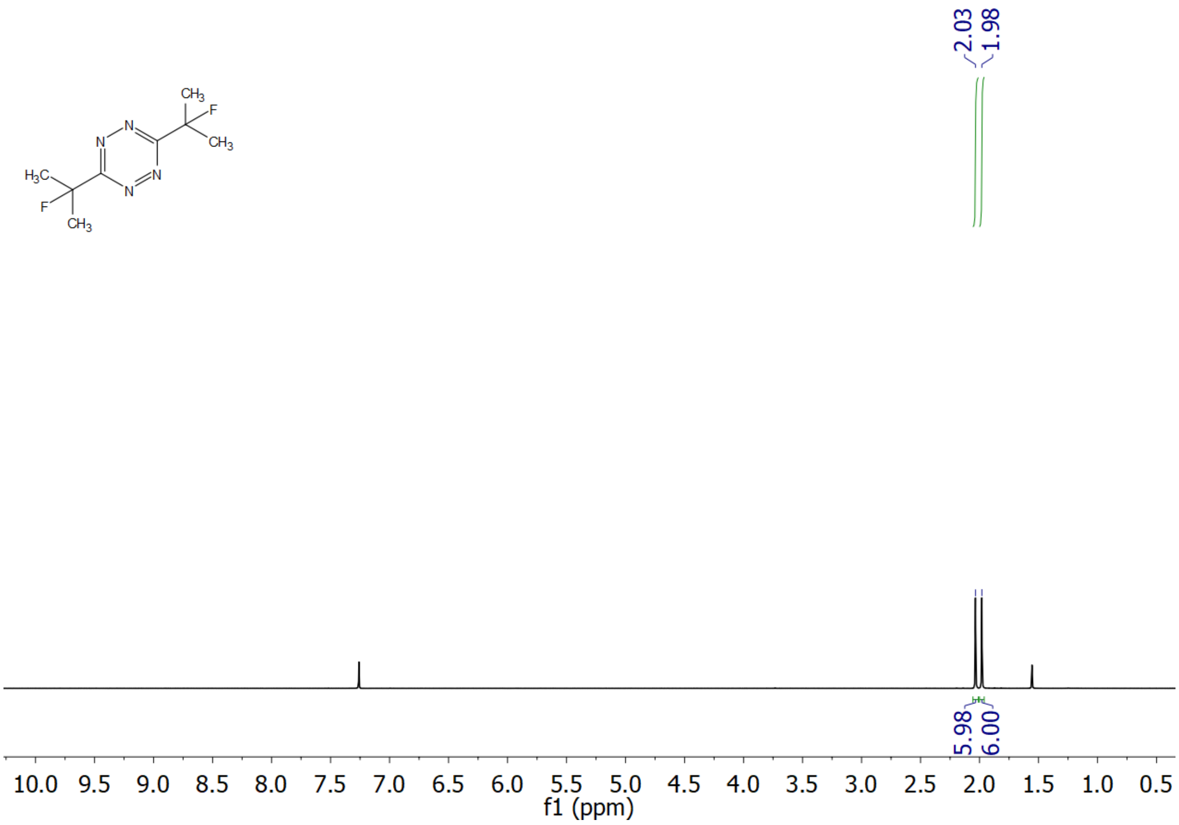

Figure S16: ^{1}H NMR spectrum of *3,6-bis(2-fluoropropan-2-yl)-1,2,4,5-tetrazine* (400 MHz; $CDCl_3$).

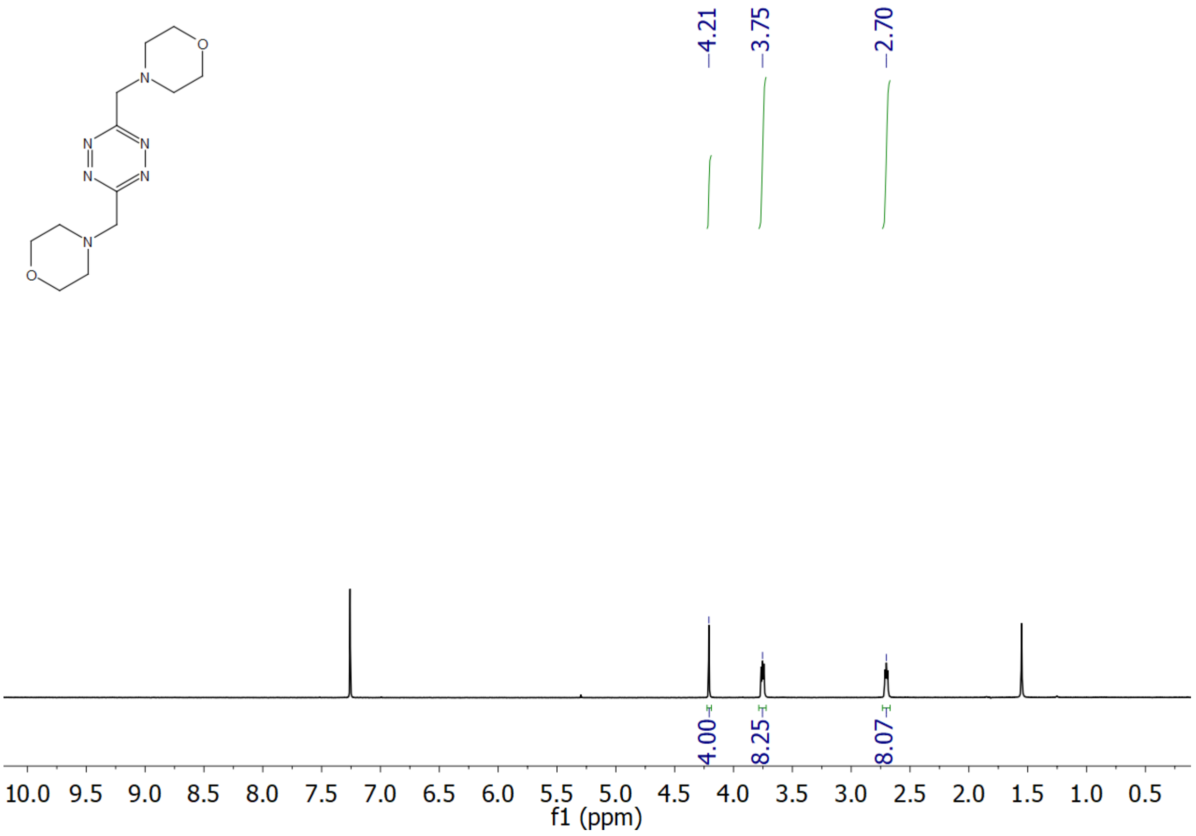

Figure S17: ^{1}H NMR spectrum of *3,6-bis(morpholinomethyl)-1,2,4,5-tetrazine* (400 MHz; $CDCl_3$).

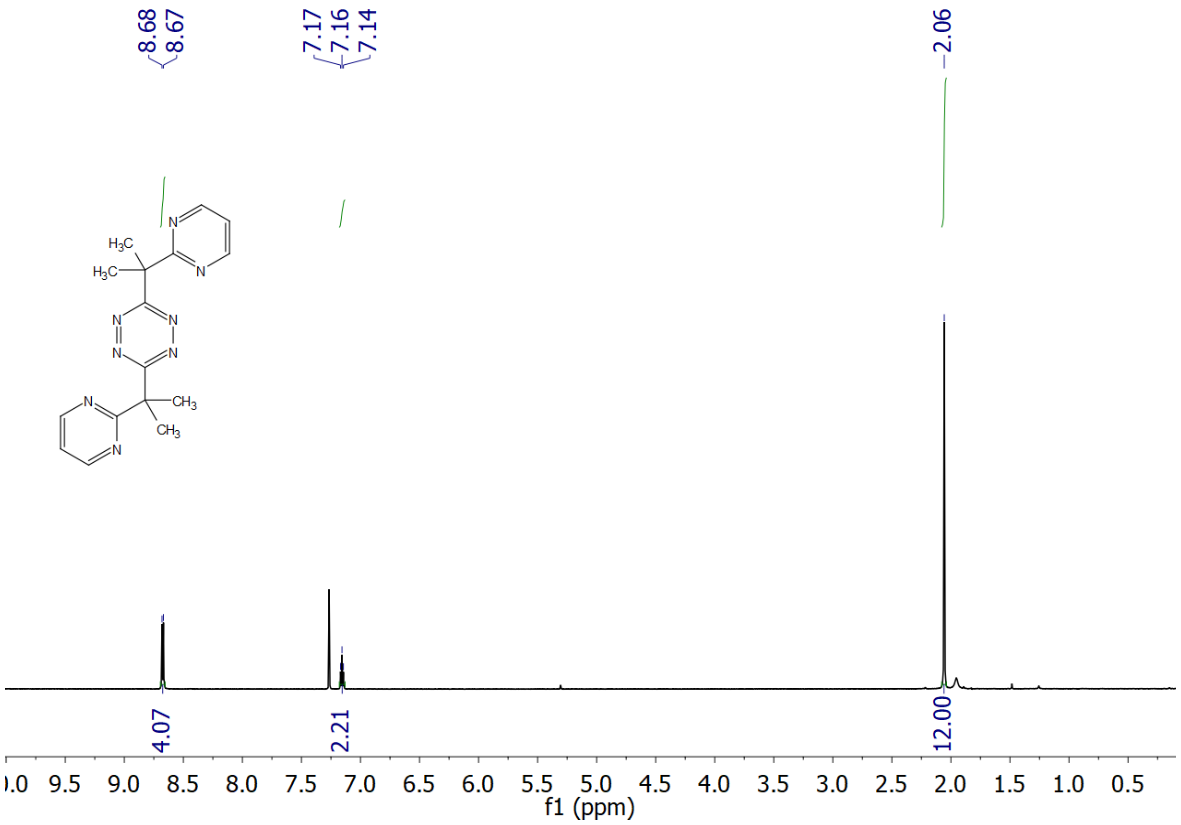

Figure S18: ^{1}H NMR spectrum of *3,6-bis(2-(pyrimidin-2-yl)propan-2-yl)-1,2,4,5-tetrazine* (400 MHz; $CDCl_3$).

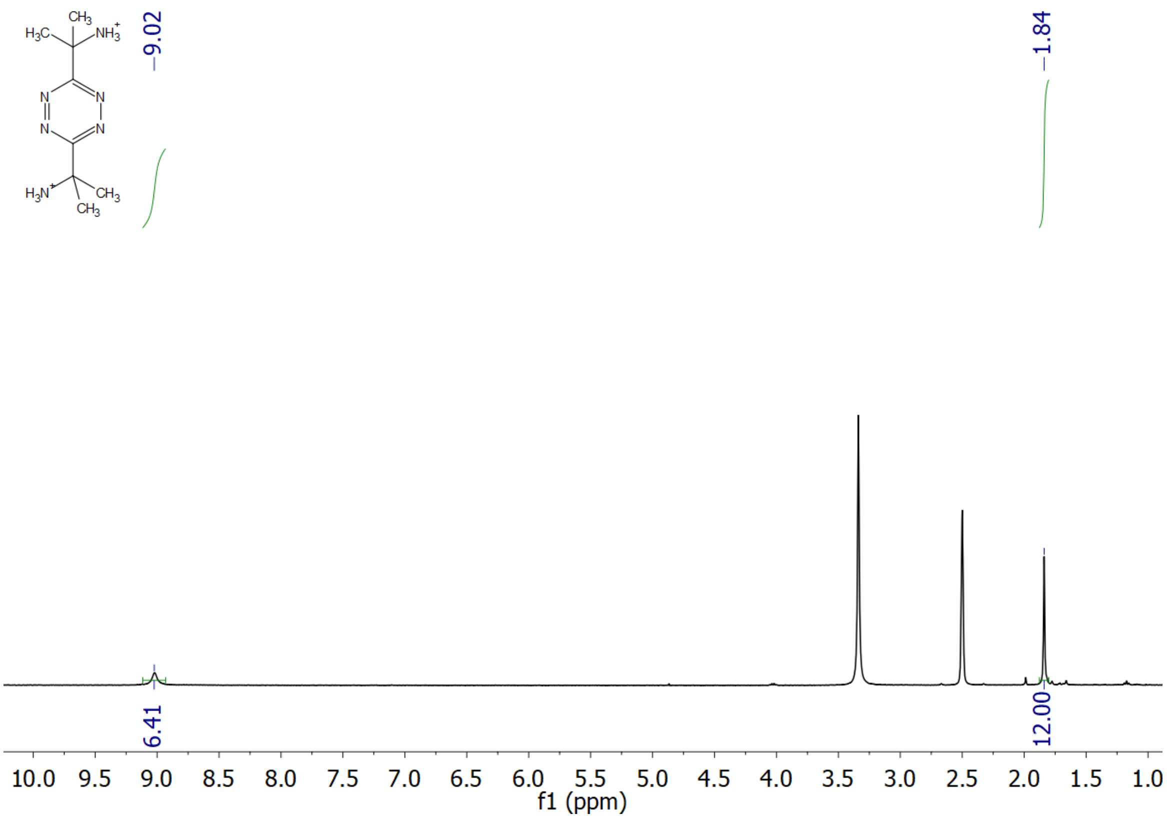

Figure S19: ^{1}H NMR spectrum of *3,6-bis(2-(pyrimidin-2-yl)propan-2-yl)-1,2,4,5-tetrazine* (400 MHz; DMSO-D_6).

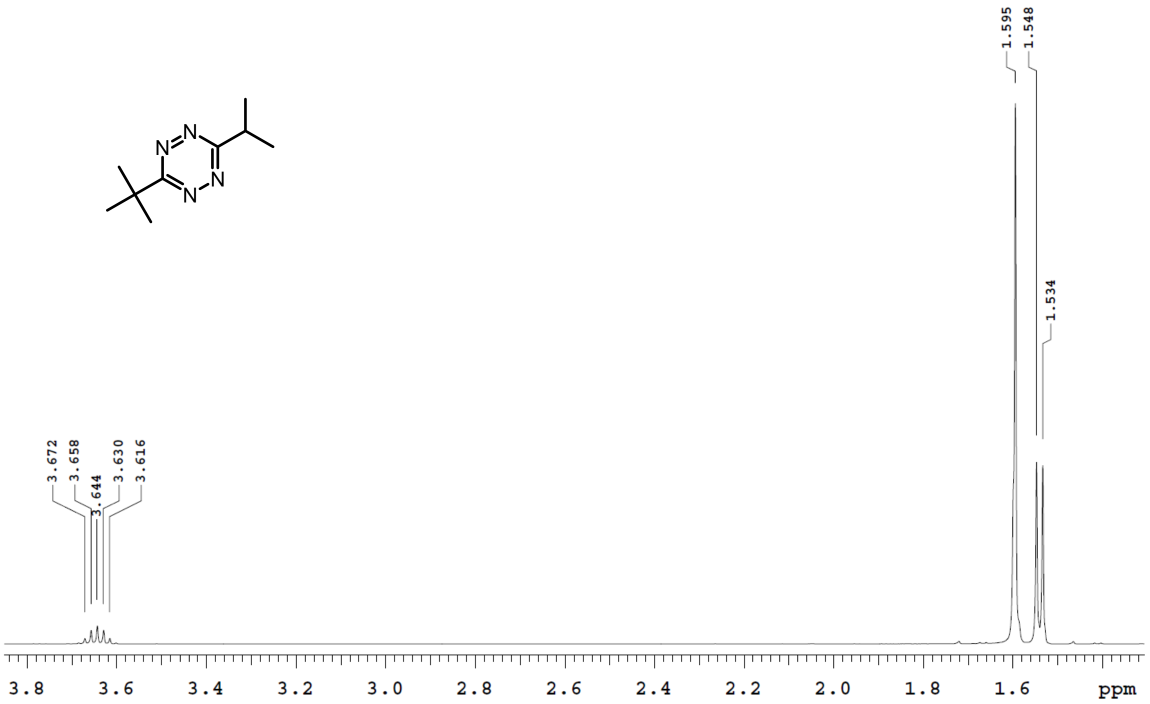

Figure S20: ^{1}H NMR spectrum of *3-tert-butyl-6-(prop-2-yl)-1,2,4,5-tetrazine* (500 MHz; $CDCl_3$).

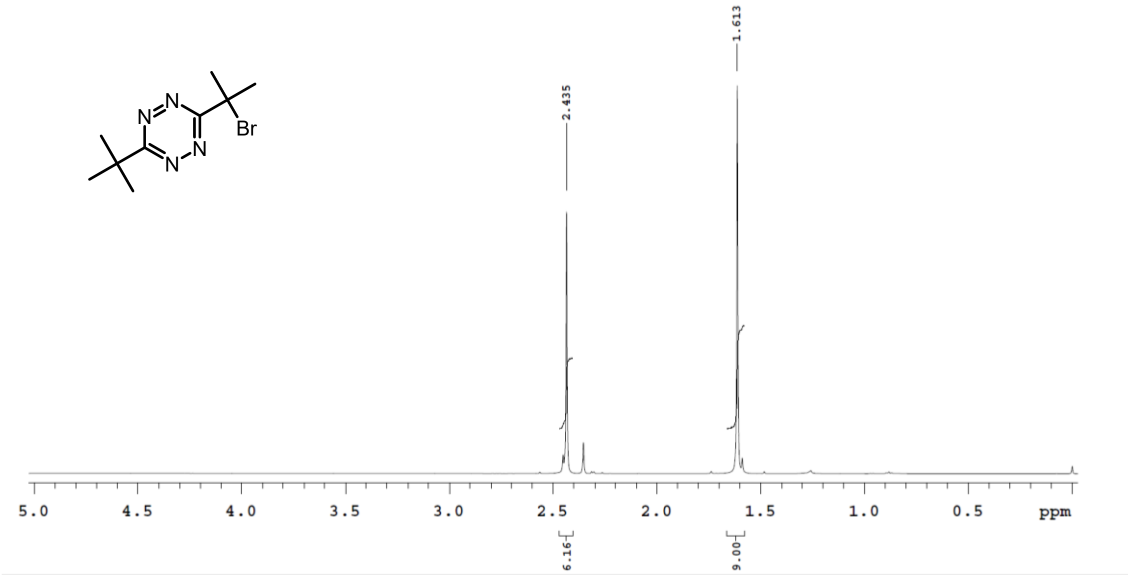

Figure S21: ^{1}H NMR spectrum of *3-(2-bromoprop-2-yl)-6-tert-butyl-1,2,4,5-tetrazine* (500 MHz; $CDCl_3$).

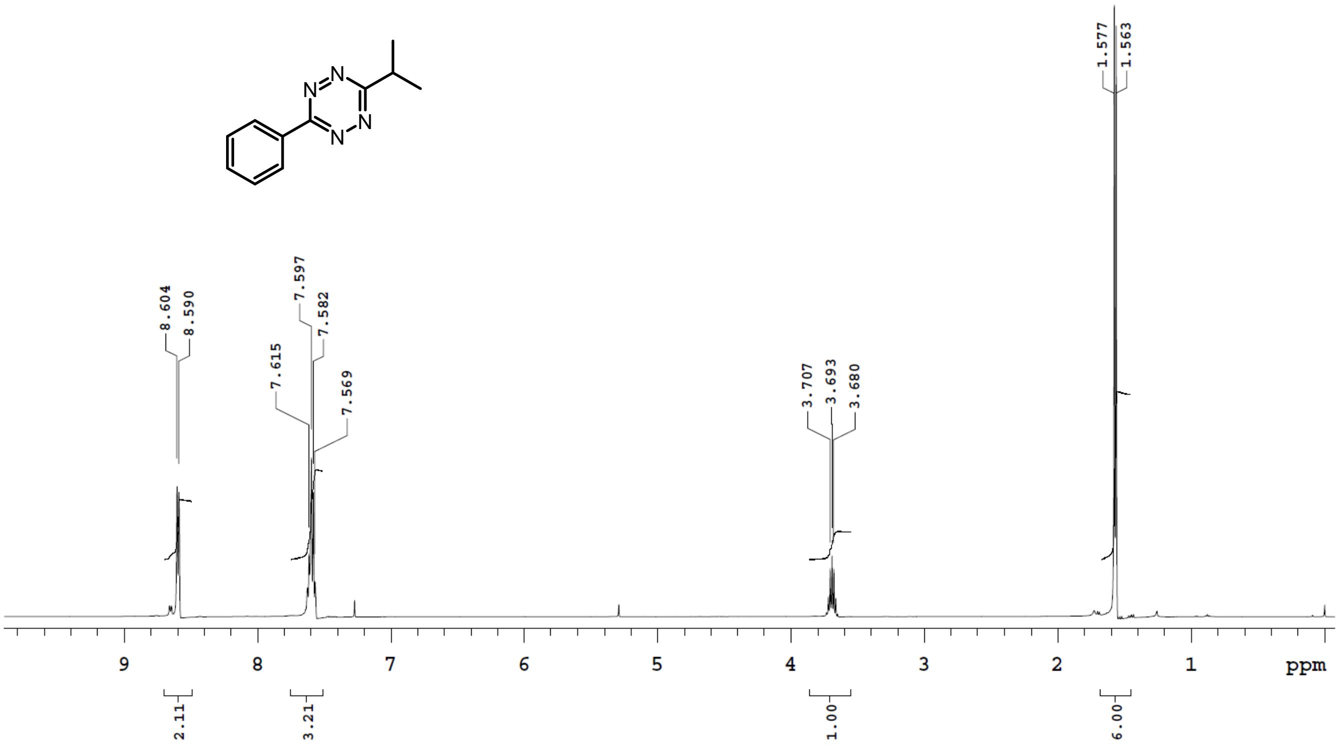

Figure S22: ^{1}H NMR spectrum of *3-phenyl-6-(prop-2-yl)-1,2,4,5-tetrazine* (500 MHz; $CDCl_3$).

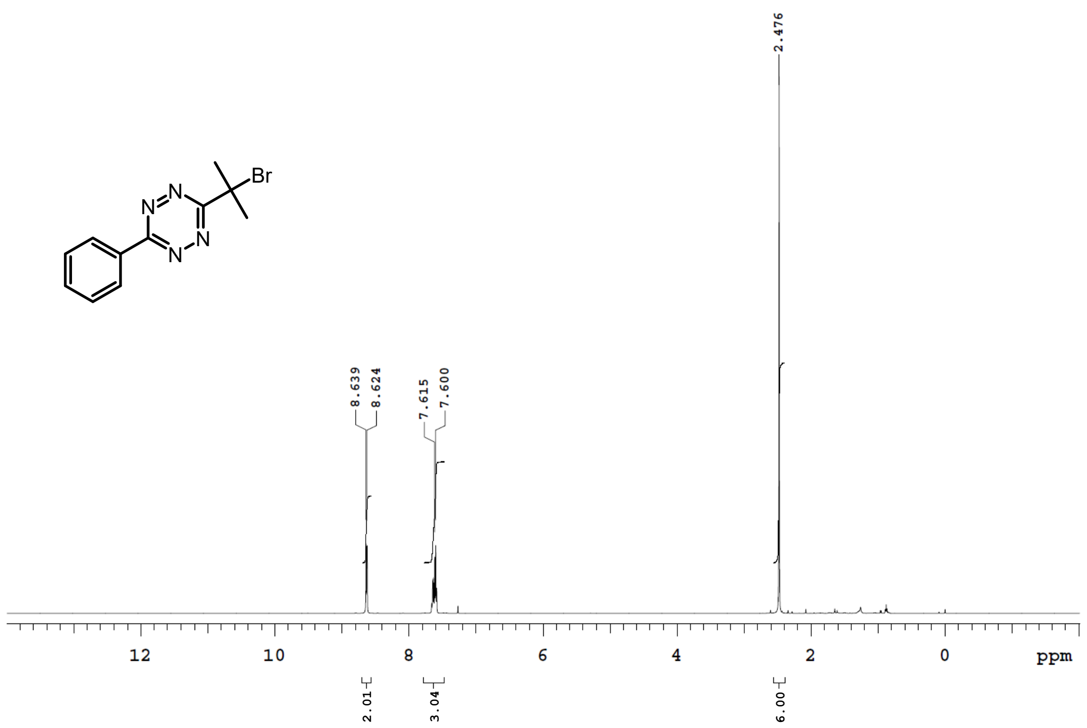

Figure S23: ^{1}H NMR spectrum of *3-(2-bromoprop-2-yl)-6-phenyl-1,2,4,5-tetrazine* (500 MHz; $CDCl_3$).

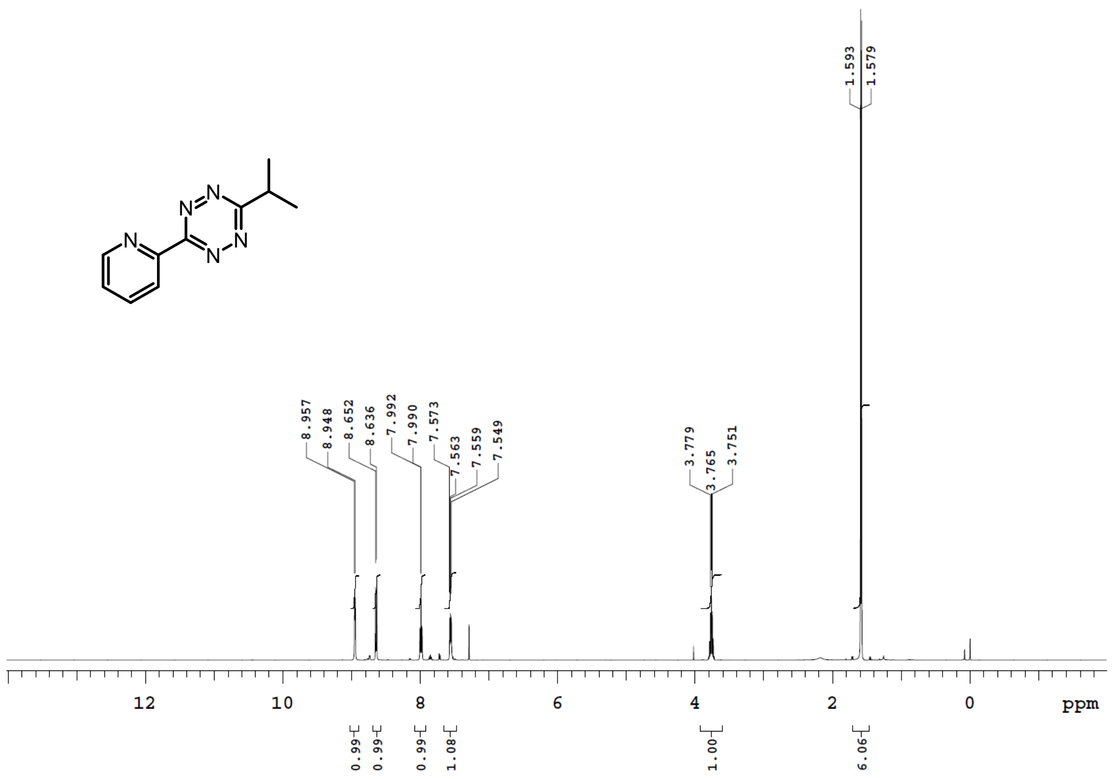

Figure S24: ^{1}H NMR spectrum of *3-(prop-2-yl)-6-(pyrid-2-yl)-1,2,4,5-tetrazine* (500 MHz; $CDCl_3$).

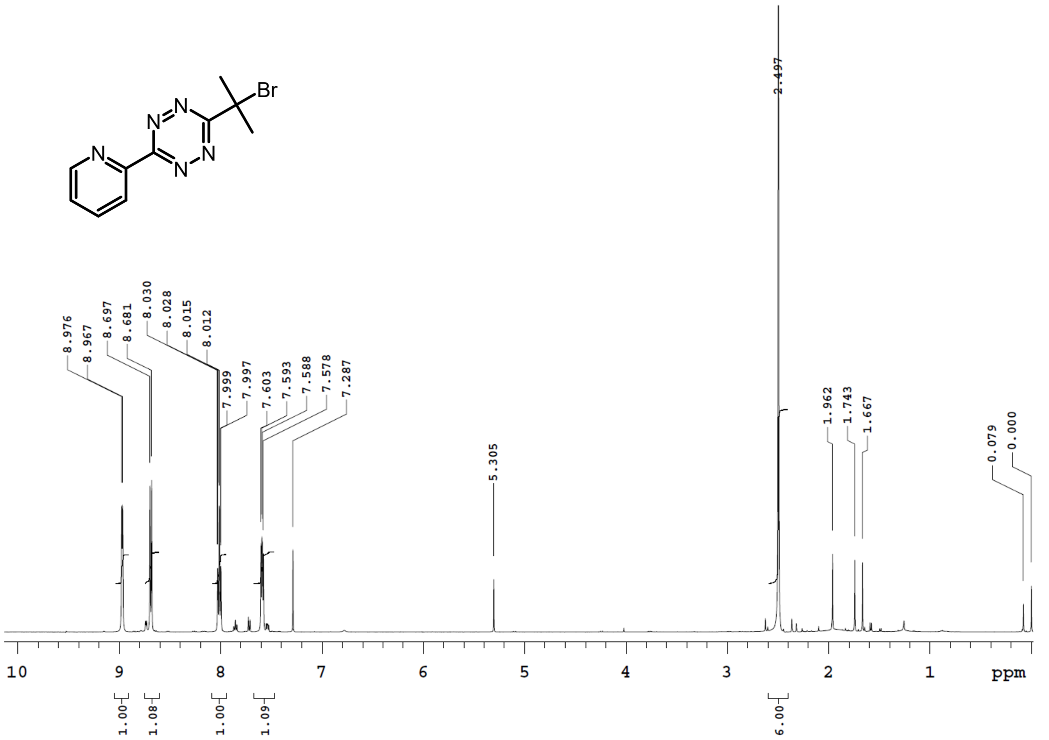

Figure S25: ^{1}H NMR spectrum of *3-(2-bromoprop-2-yl)-6-(pyrid-2-yl)-1,2,4,5-tetrazine* (500 MHz; $CDCl_3$).

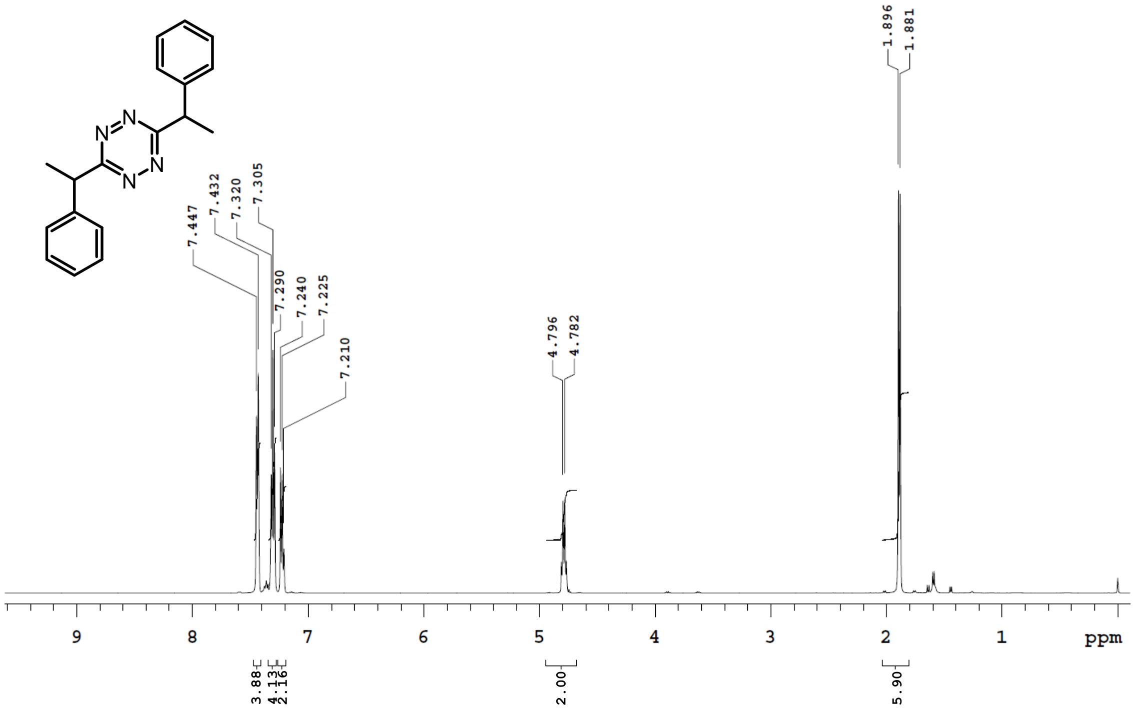

Figure S26: ^{1}H NMR spectrum of *3,6-bis(1-phenylethyl)-1,2,4,5-tetrazine* (500 MHz; $CDCl_3$).

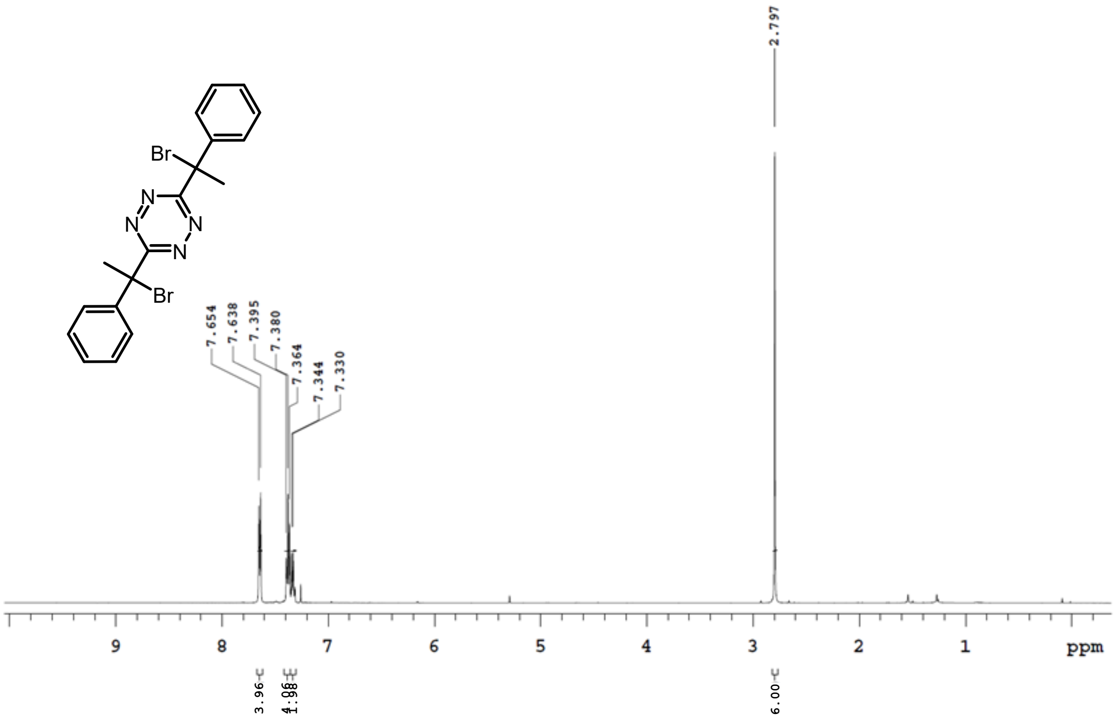

Figure S27: ^{1}H NMR spectrum of *3,6-bis(1-bromo-1-phenylethyl)-1,2,4,5-tetrazine* (500 MHz; $CDCl_3$).

^{19}F NMR spectra of synthesized compounds

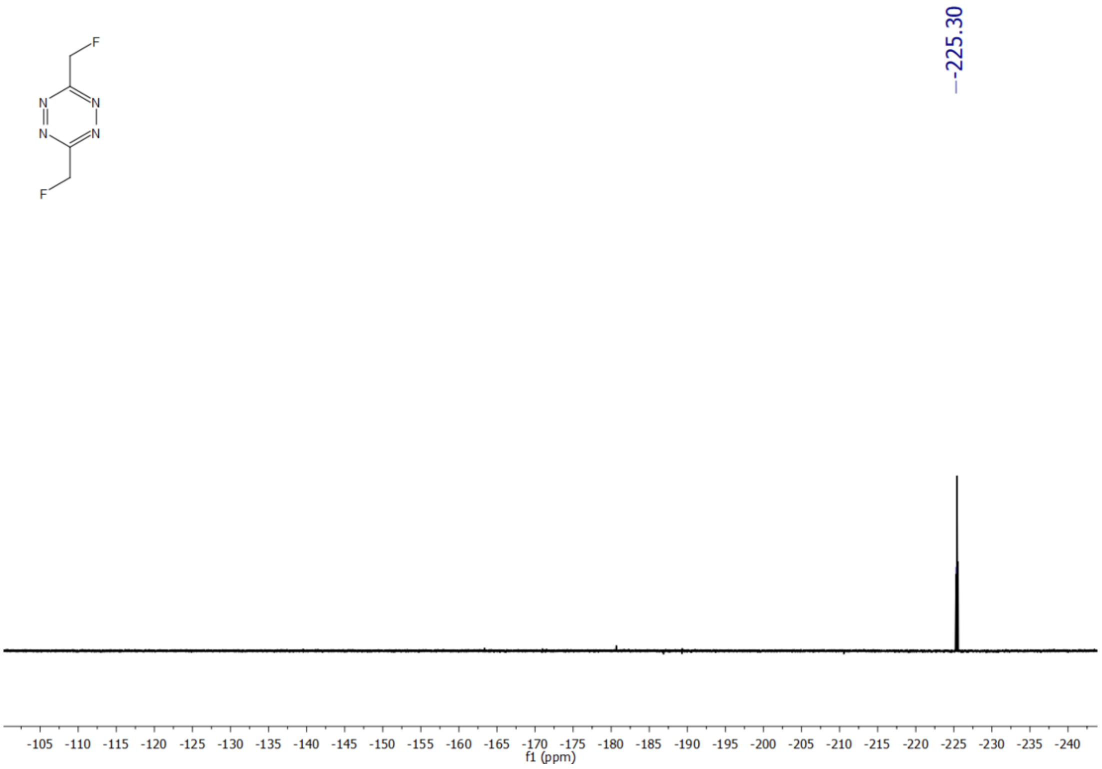

Figure S28: ^{19}F NMR of *3,6-bis(fluoromethyl)-1,2,4,5-tetrazine* (400 MHz, $CDCl_3$).

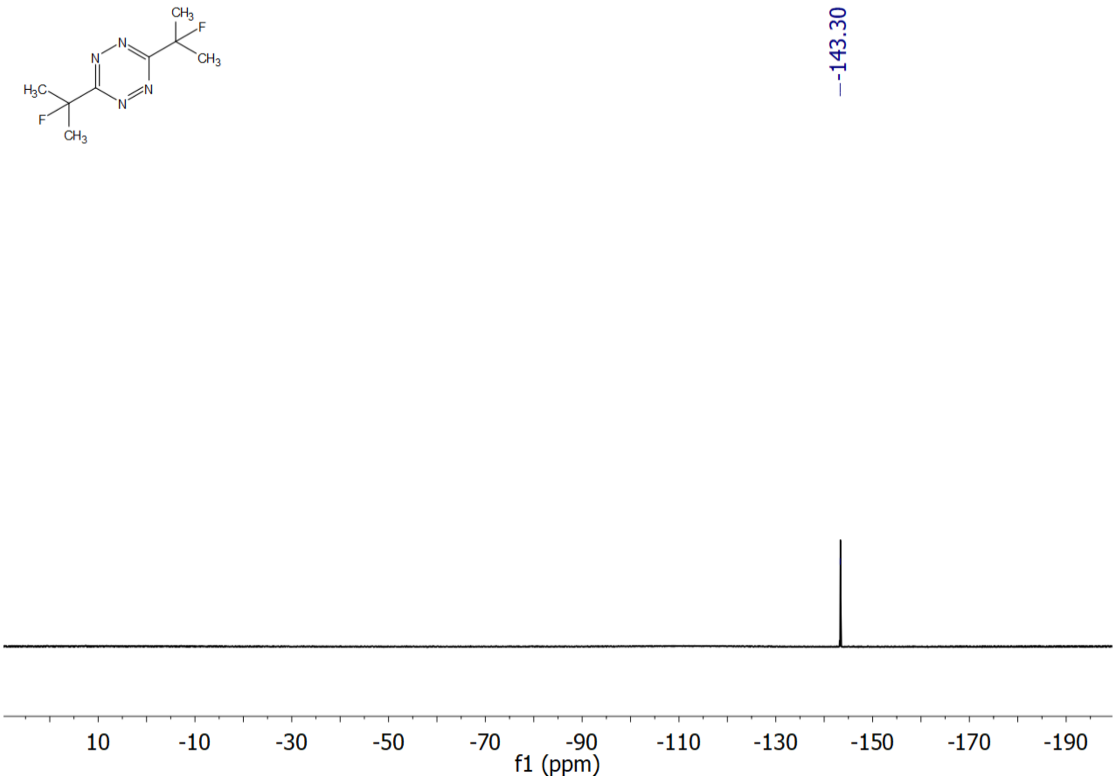

Figure S29: ^{19}F NMR of *3,6-bis(2-fluoropropan-2-yl)-1,2,4,5-tetrazin*e (400 MHz, $CDCl_3$).

^{13}C-NMR of synthesized compounds

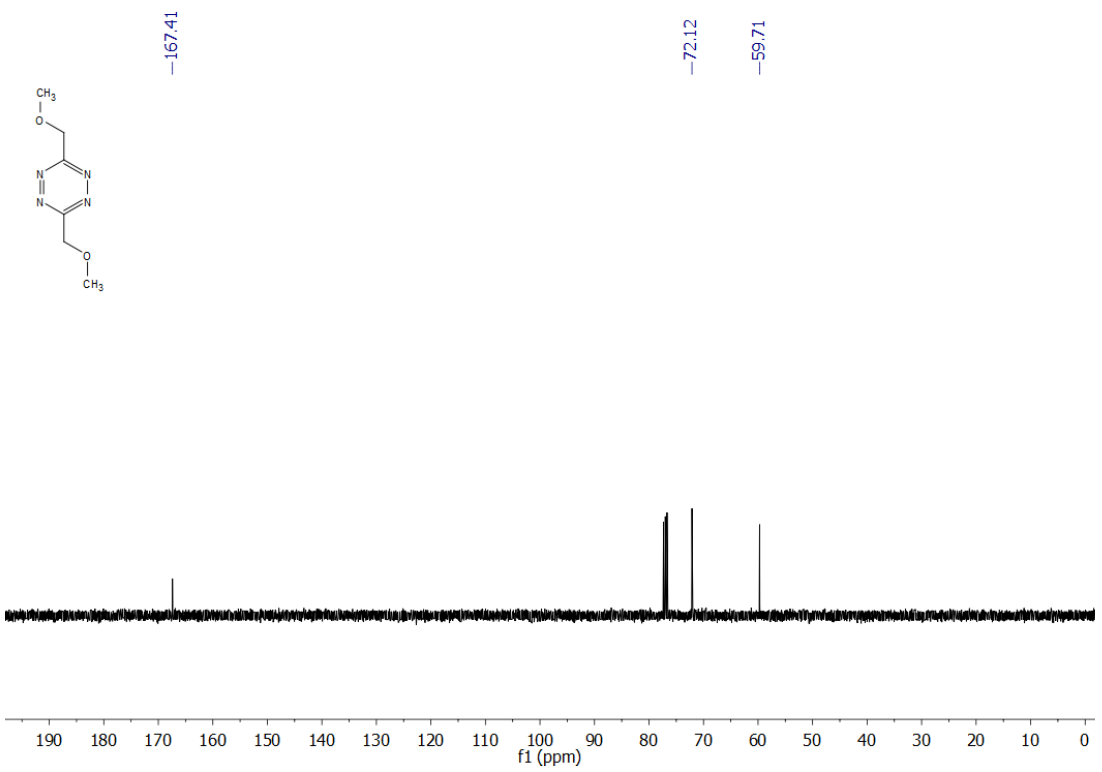

Figure S30: ^{13}C NMR of the *3,6-bis(methoxymethyl)-1,2,4,5-tetrazine* (125 MHz; $CDCl_3$).

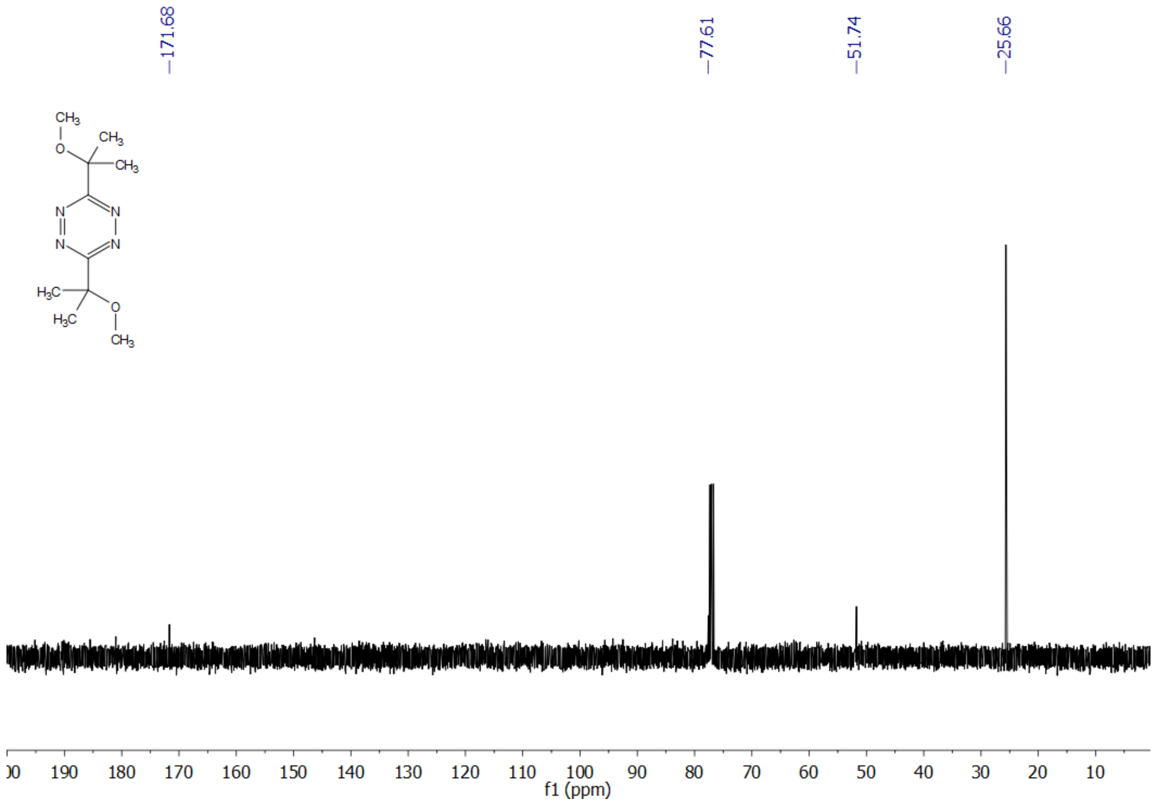

Figure S31: ^{13}C NMR of 3,6-bis(2-methoxyprop-2-yl)-1,2,4,5-tetrazine (125 MHz; $CDCl_3$).

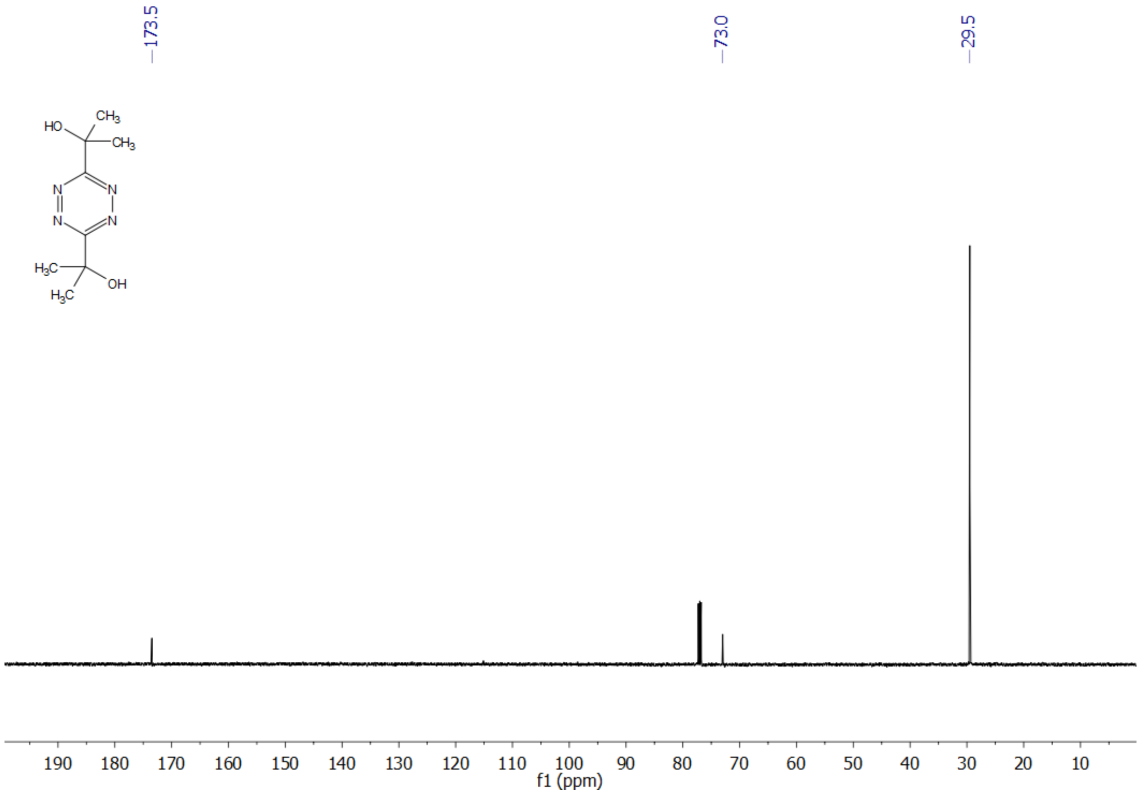

Figure S32: ^{13}C NMR of *2,2'-(1,2,4,5-tetrazine-3,6-diyl)bis(propane-2-ol) in* (125 MHz; $CDCl_3$).

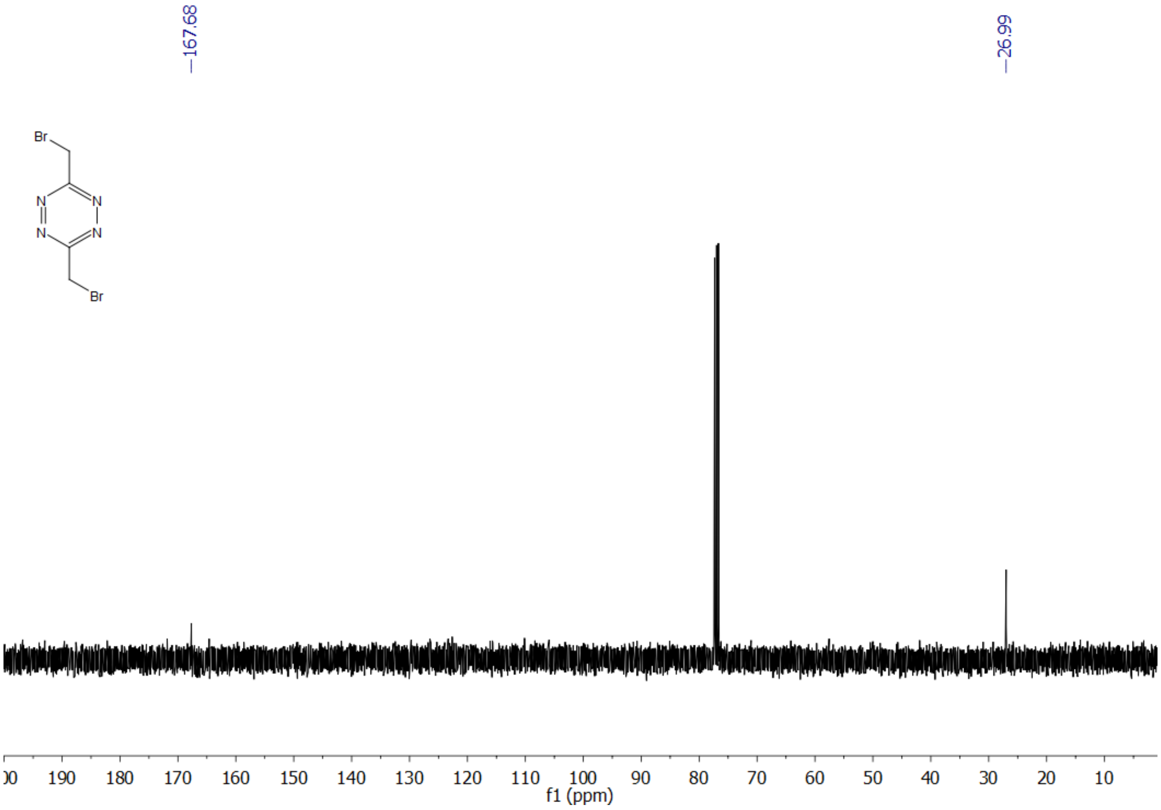

Figure S33: ^{13}C NMR of *3,6-bis(bromomethyl)-1,2,4,5-tetrazine* in (125 MHz; $CDCl_3$).

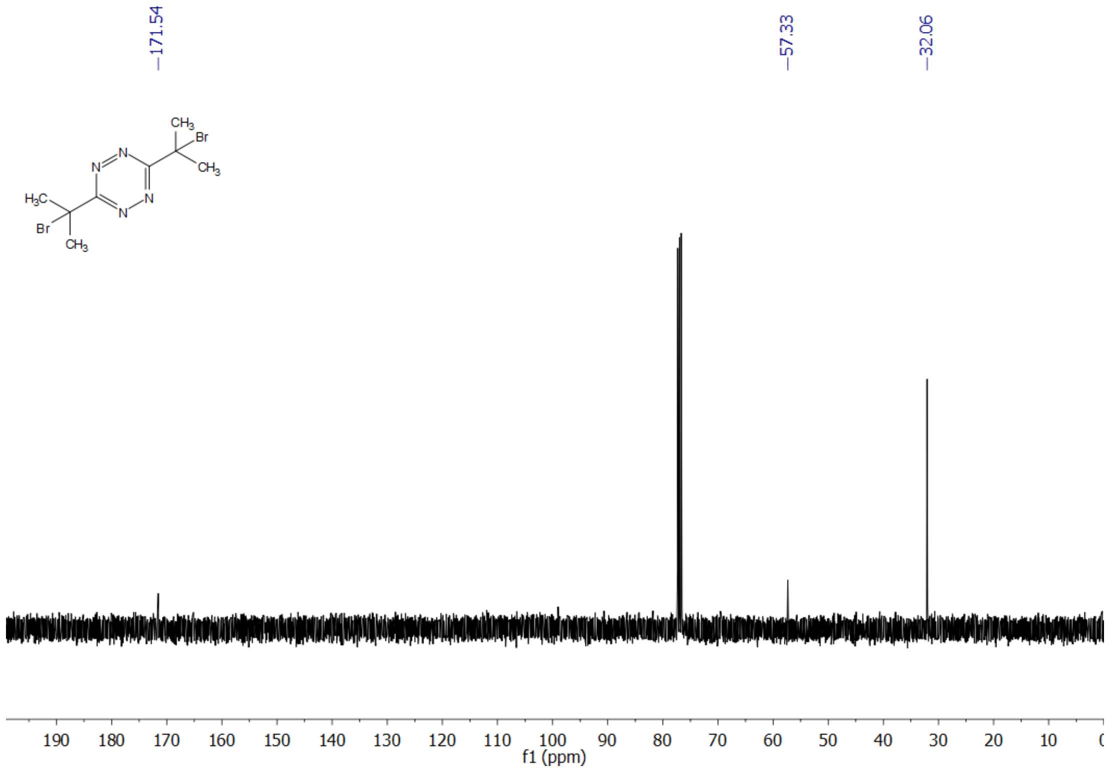

Figure S34: ^{13}C NMR of *3,6-bis(2-bromopropan-2-yl)-1,2,4,5-tetrazine* (125 MHz; $CDCl_3$).

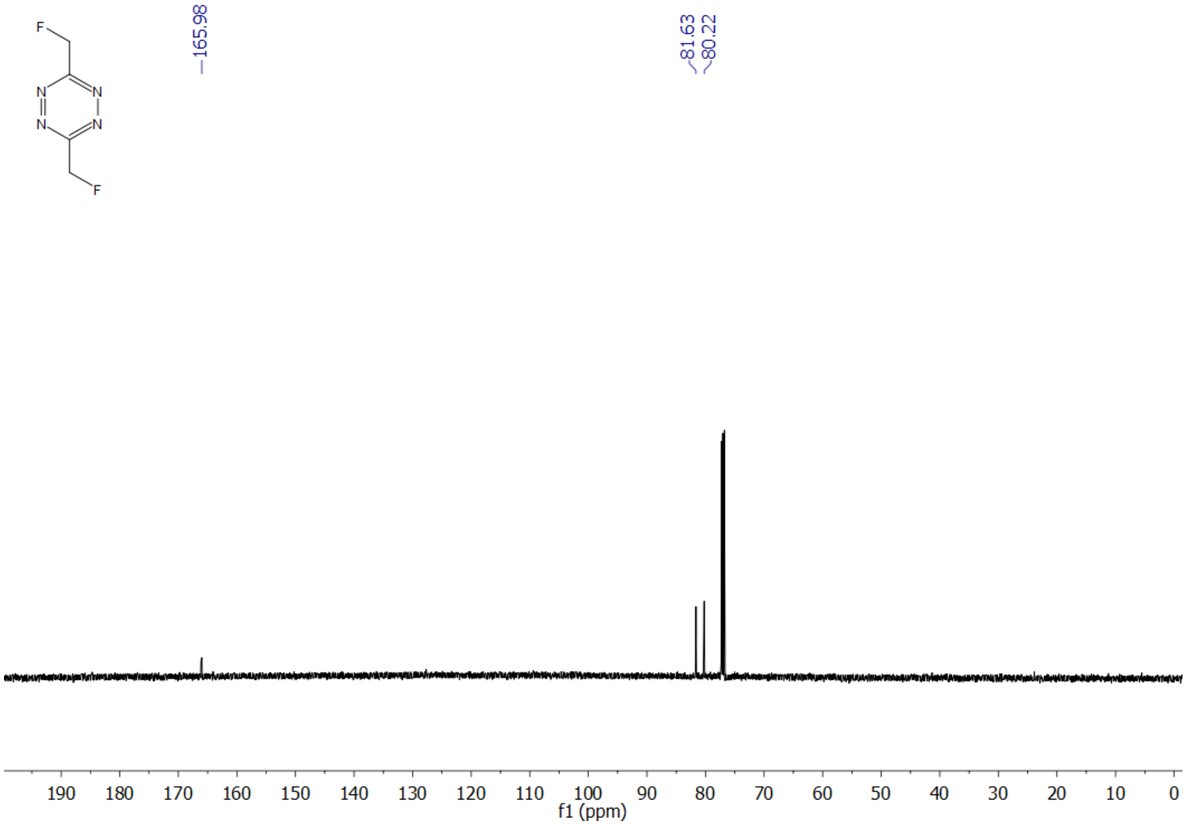

Figure S35: ^{13}C NMR of *3,6-bis(fluoromethyl)-1,2,4,5-tetrazine* (125 MHz; $CDCl_3$).

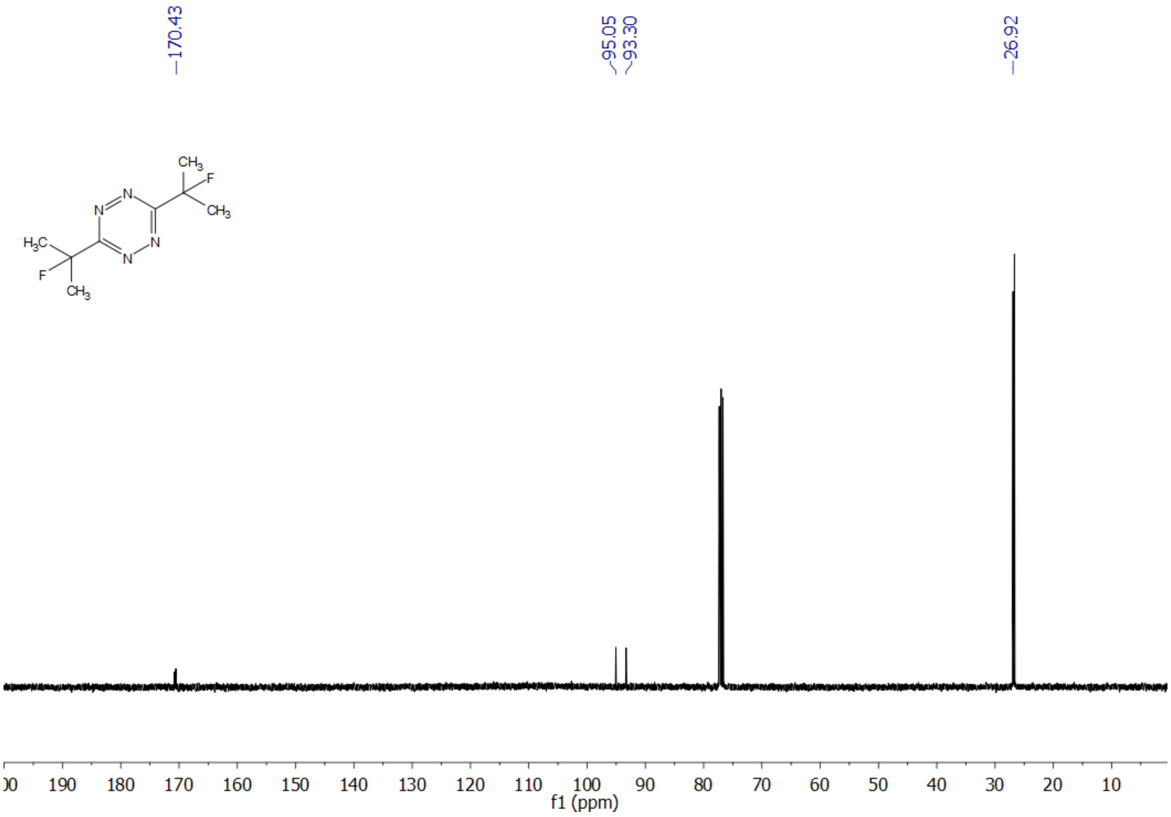

Figure S36: ^{13}C NMR of *3,6-bis(2-fluoroprop-2-yl)-1,2,4,5-tetrazin*e (125 MHz; $CDCl_3$).

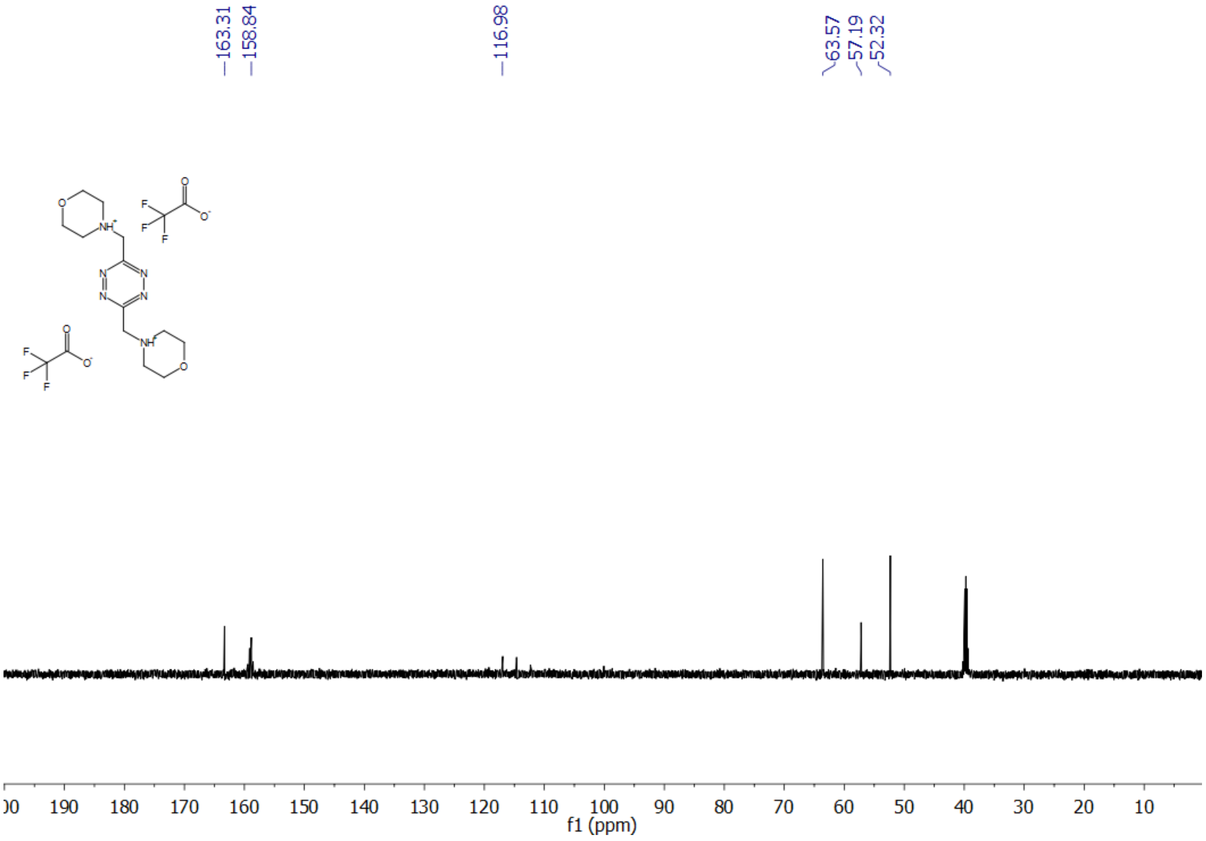

Figure S37: ^{13}C-NMR of *3,6-bis(morpholinomethyl)-1,2,4,5-tetrazine* (125 MHz; DMSO-d_6).

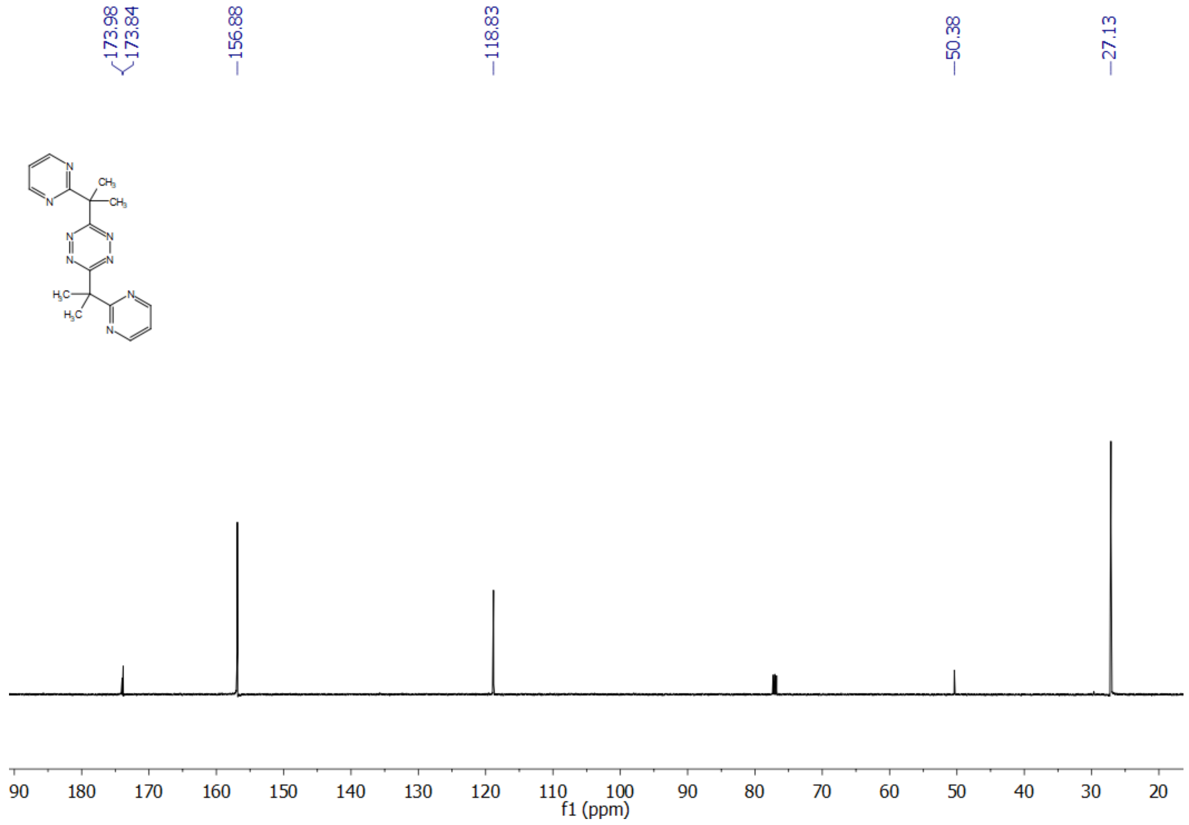

Figure S38: ^{13}C NMR of *3,6-bis(2-(pyrimidin-2-yl)prop-2-yl)-1,2,4,5-tetrazine* (125 MHz; $CDCl_3$).

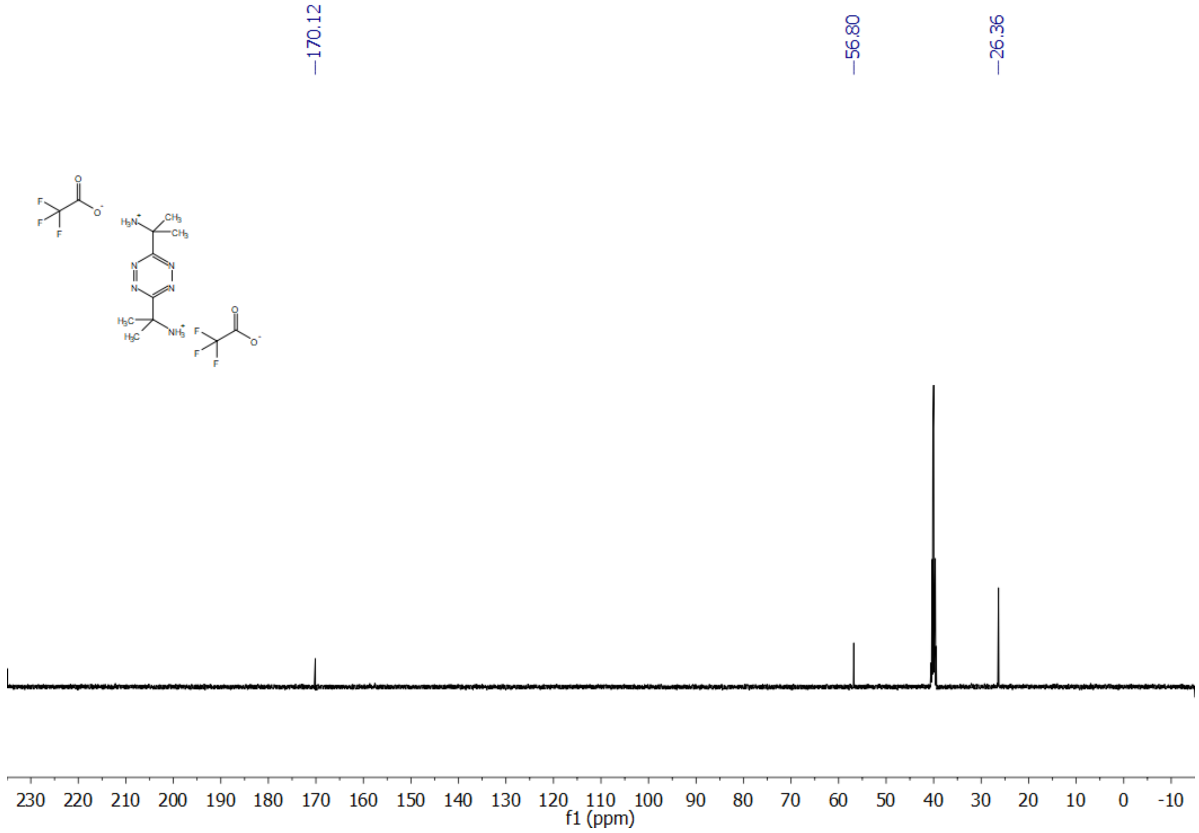

Figure S39: ^{13}C NMR of *2,2'-(1,2,4,5-tetrazine-3,6-diyl)bis(propan-2-aminium)* (125 MHz; DMSO-d_6).

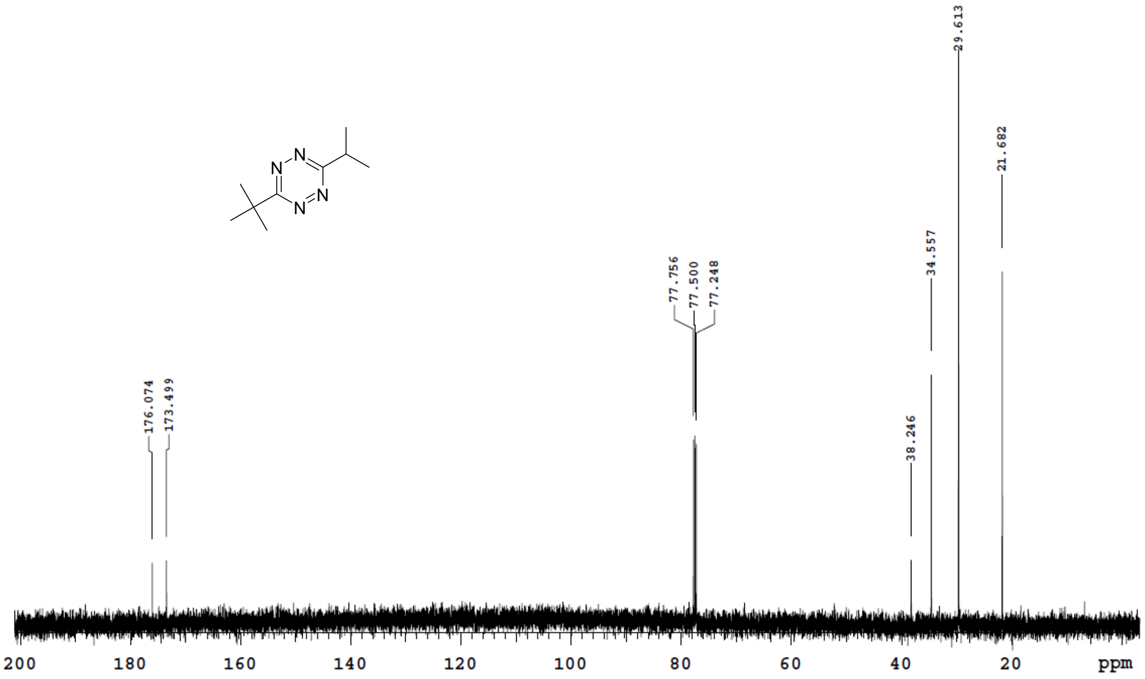

Figure S40: ^{13}C NMR spectrum of *3-tert-butyl-6-(prop-2-yl)-1,2,4,5-tetrazine* (125 MHz; $CDCl_3$).

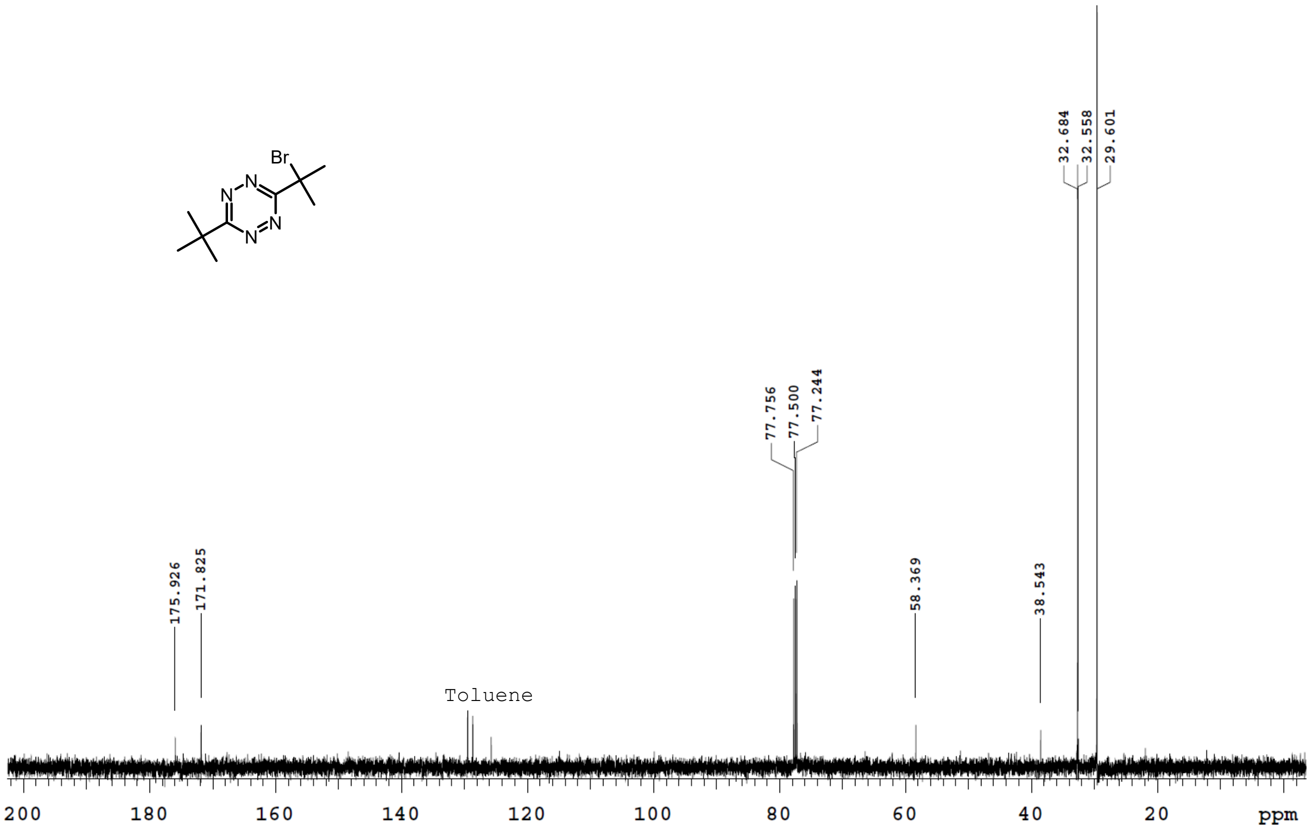

Figure S41: ^{13}C NMR spectrum of *3-(2-bromoprop-2-yl)-6-tert-butyl-1,2,4,5-tetrazine* (125 MHz; $CDCl_3$).

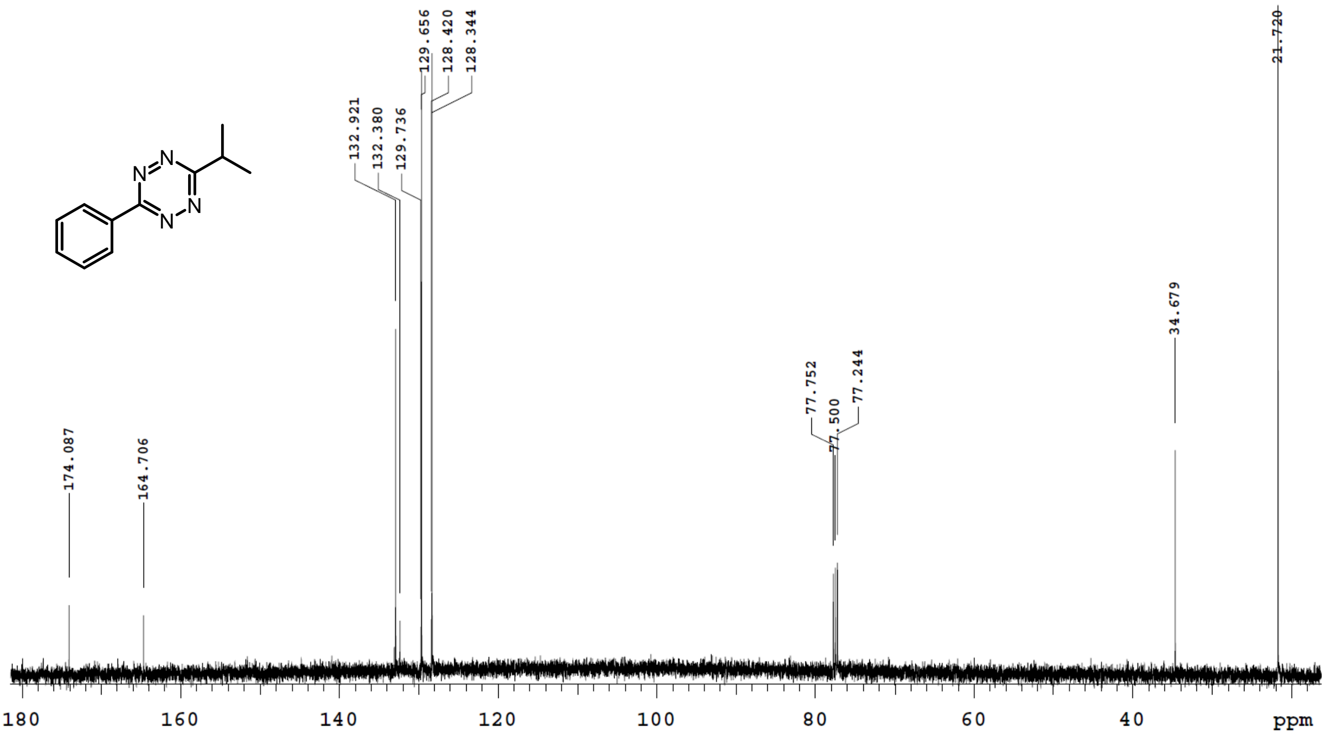

Figure S42: ^{13}C NMR spectrum of *3-phenyl-6-(prop-2-yl)-1,2,4,5-tetrazine* (125 MHz; $CDCl_3$).

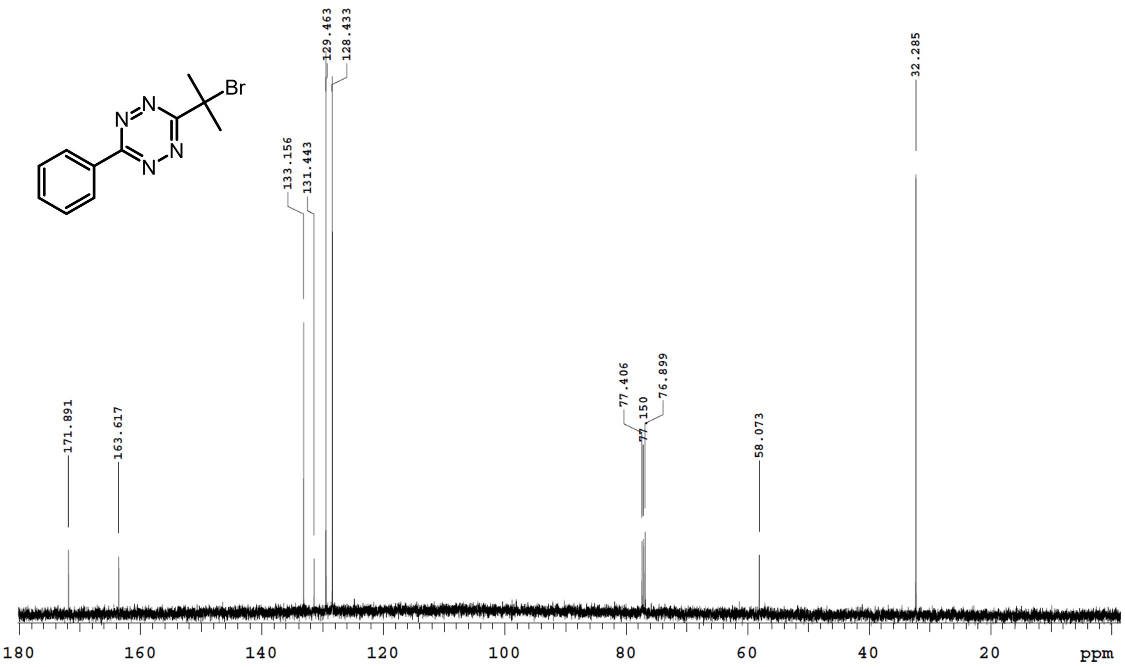

Figure S43: ^{13}C NMR spectrum of *3-(2-bromoprop-2-yl)-6-phenyl-1,2,4,5-tetrazine* (125 MHz; $CDCl_3$).

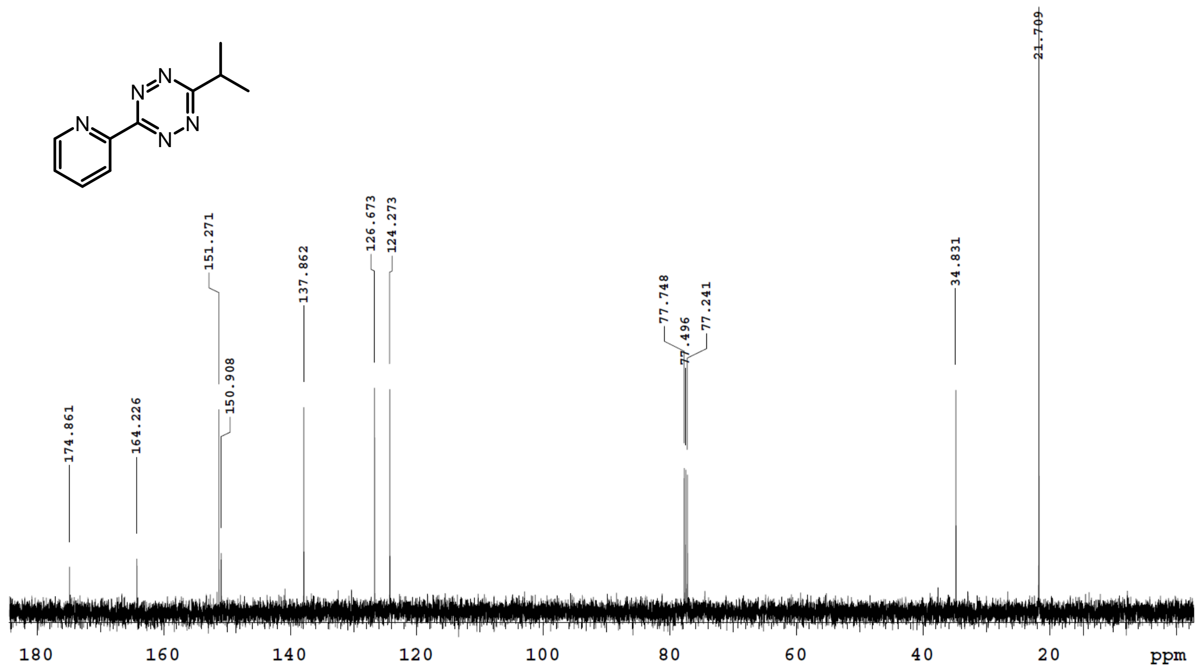

Figure S44: ^{13}C NMR spectrum of *3-(prop-2-yl)-6-(pyrid-2-yl)-1,2,4,5-tetrazine* (125 MHz; $CDCl_3$).

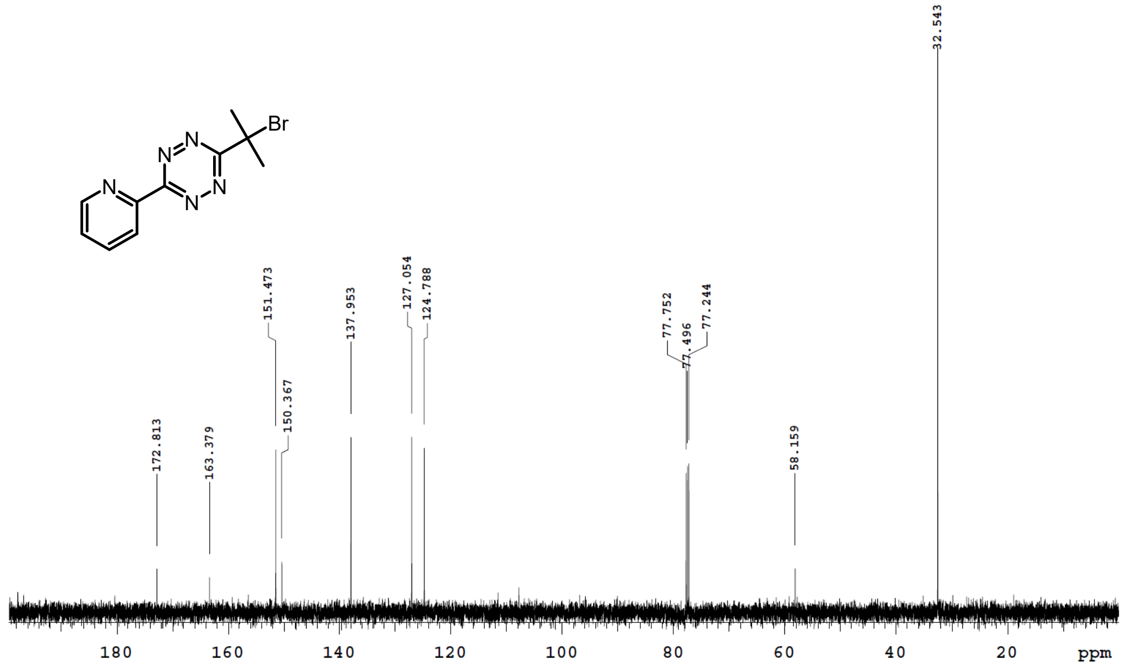

Figure S45: ^{13}C NMR spectrum of *3-(2-bromoprop-2-yl)-6-(pyrid-2-yl)-1,2,4,5-tetrazine* (125 MHz; $CDCl_3$).

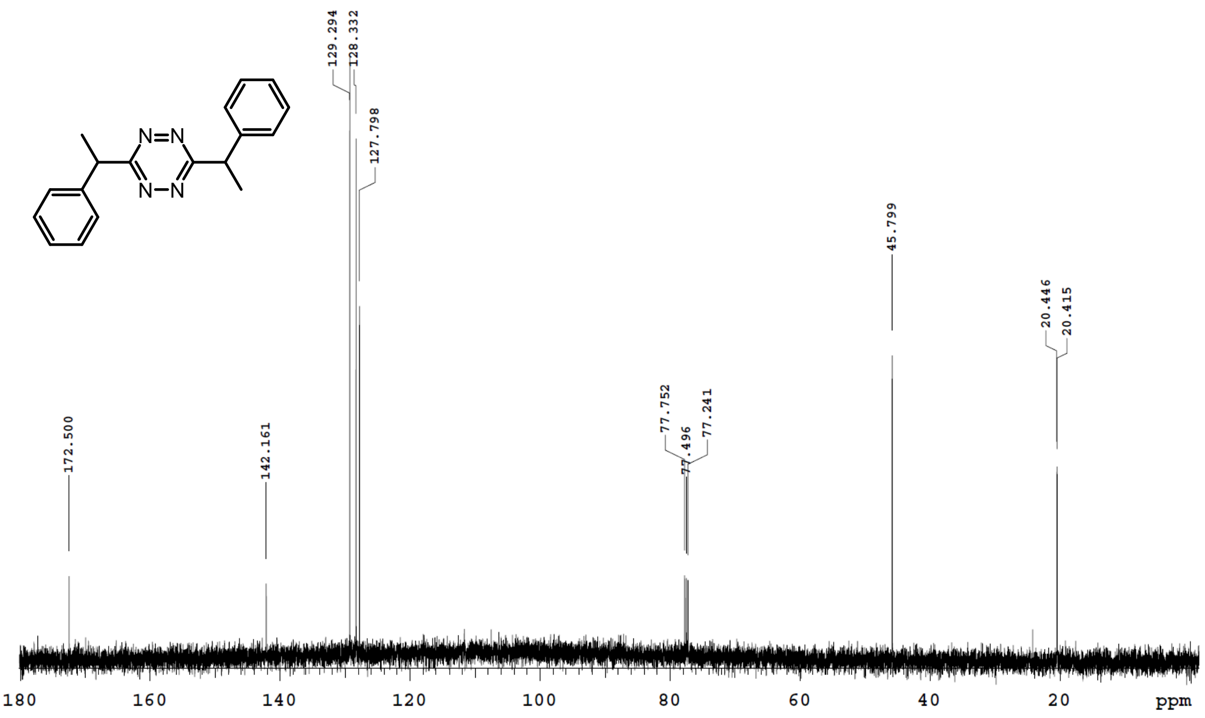

Figure S46: ^{13}C NMR spectrum of *3,6-bis(1-phenylethyl)-1,2,4,5-tetrazine* (125 MHz; $CDCl_3$).

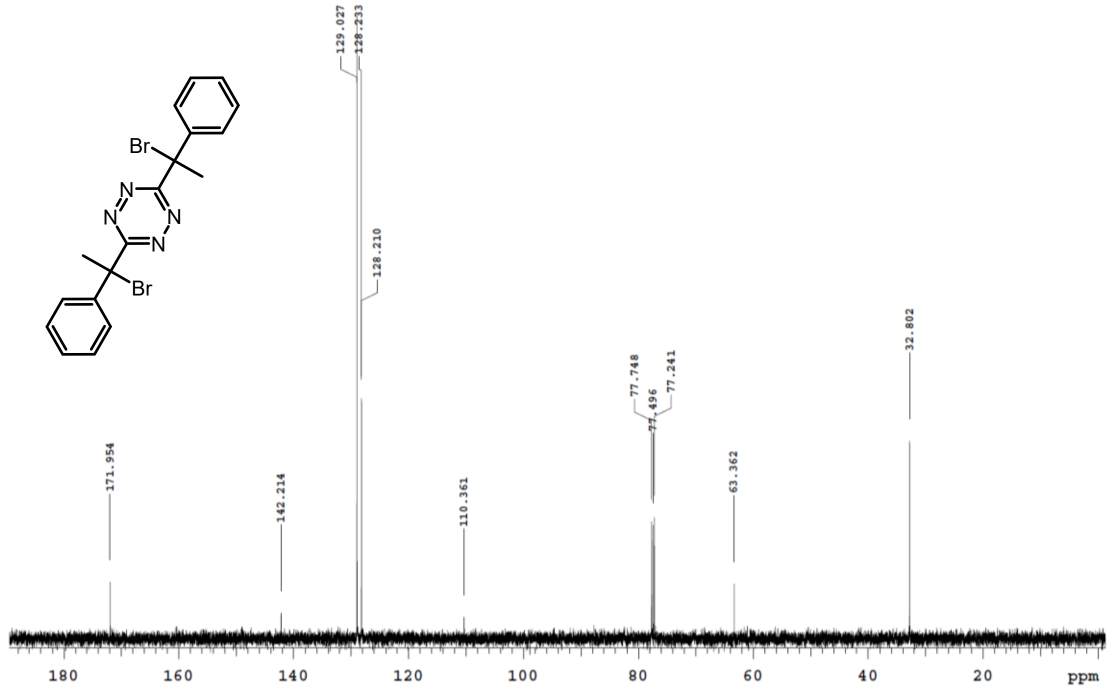

Figure S47: ^{13}C NMR spectrum of *3,6-bis(1-bromo-1-phenylethyl)-1,2,4,5-tetrazine* (125 MHz; $CDCl_3$).

Mass spectrometry data of the synthesized compounds

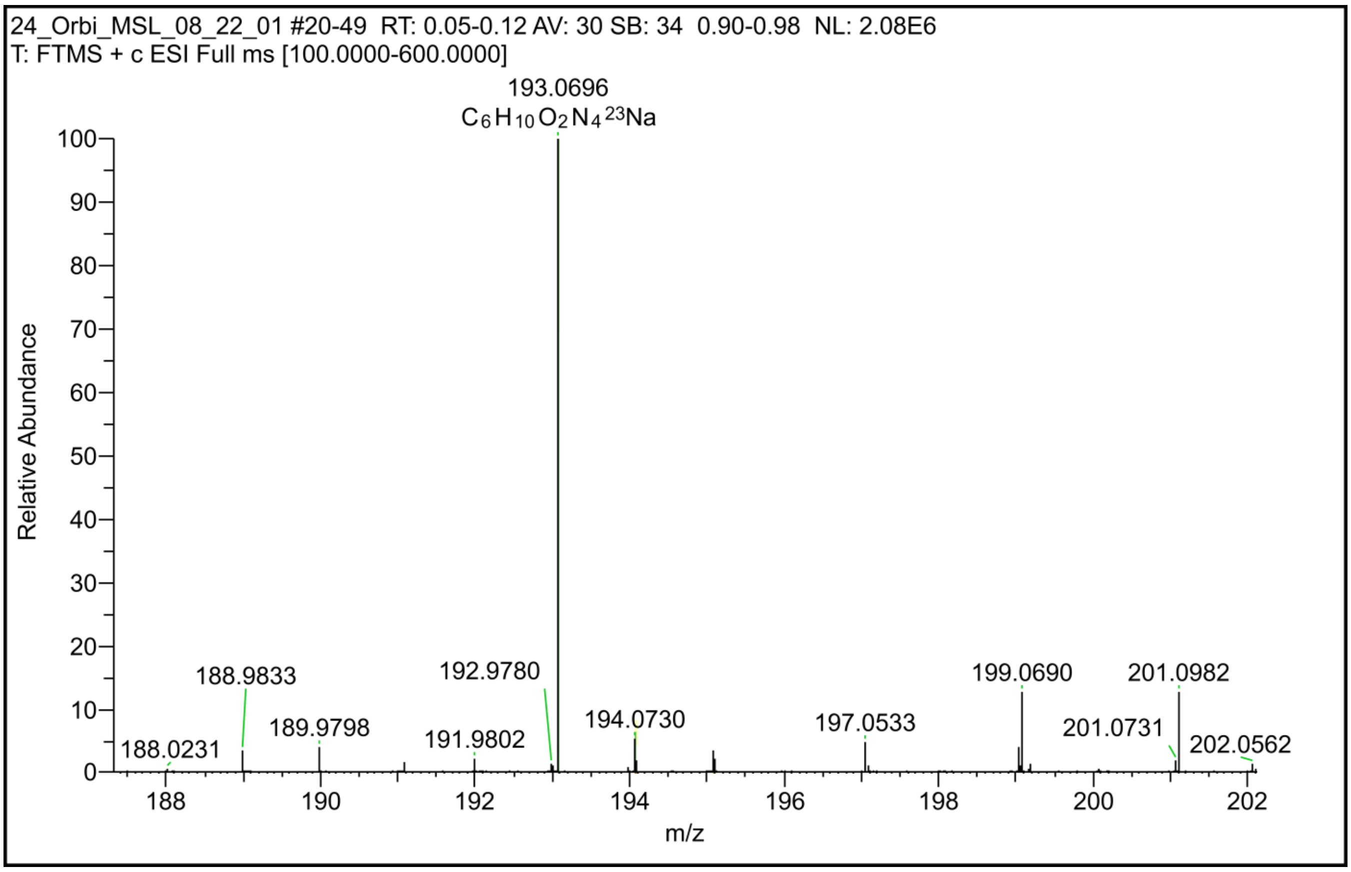

Figure S48: ESI-HRMS of compound **2a**.

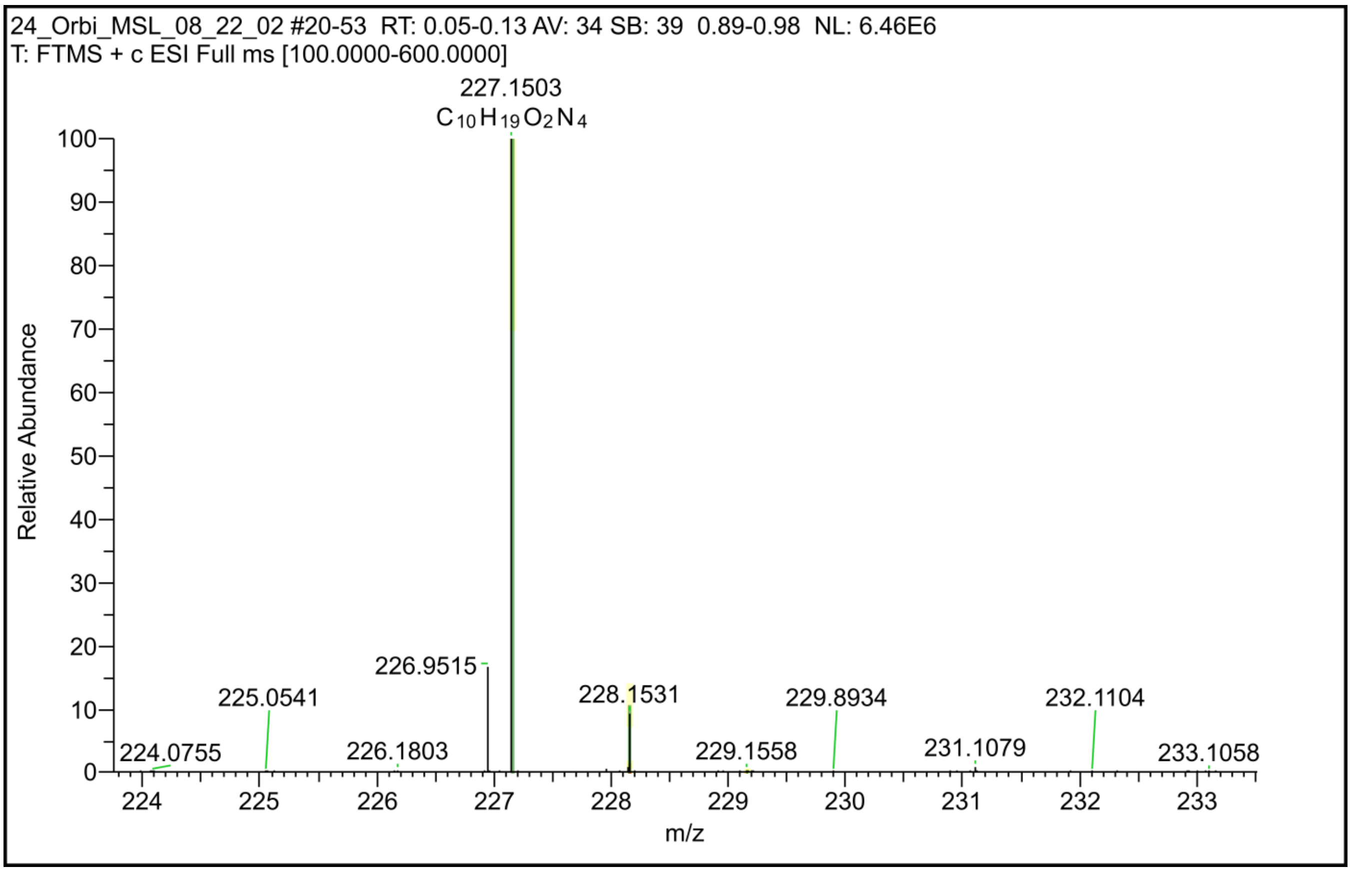

Figure S49: ESI-HRMS of compound **2b**.

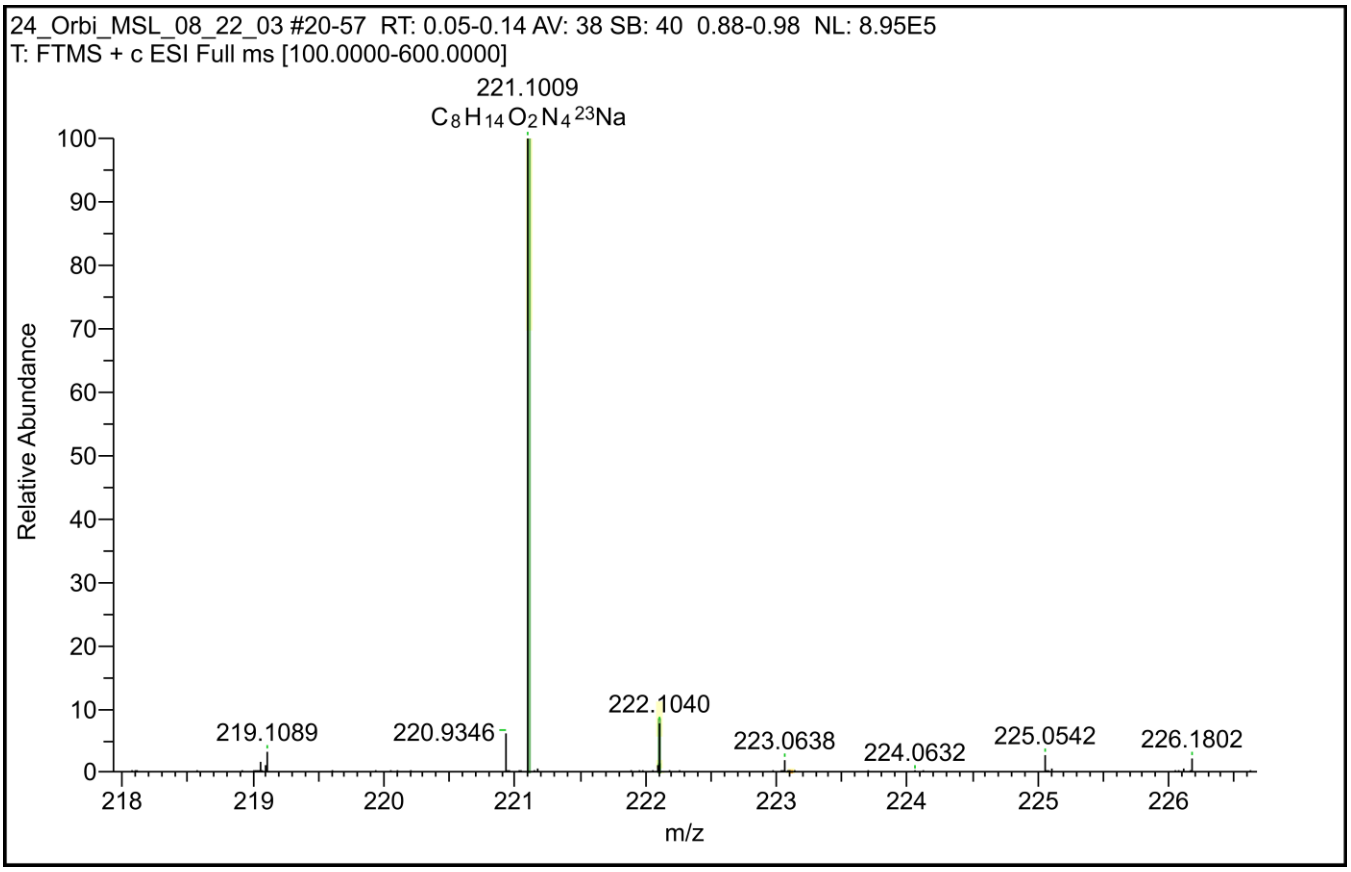

Figure S50: ESI-HRMS of compound **3b**.

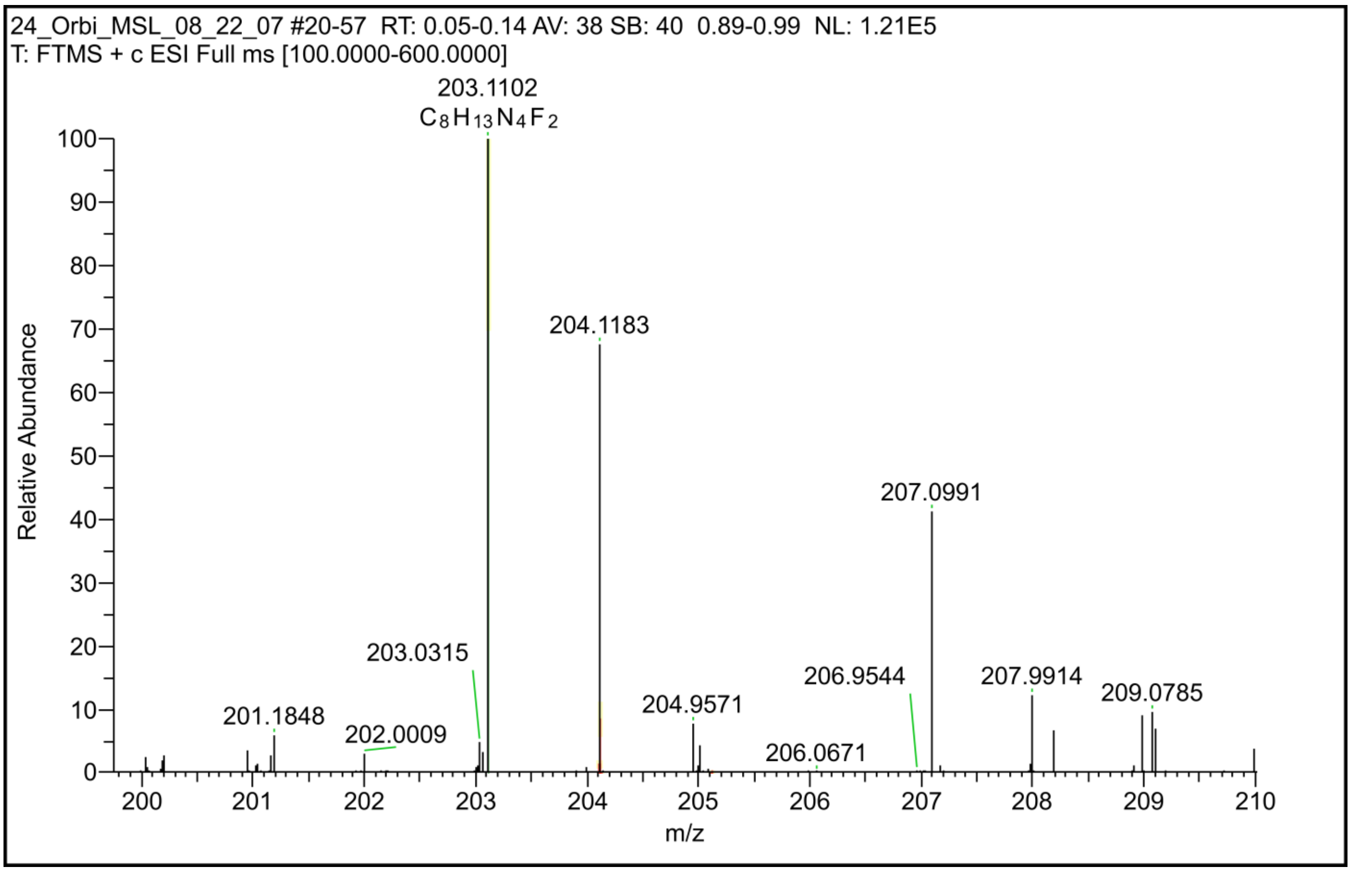

Figure S51: ESI-HRMS of compound **4b**.

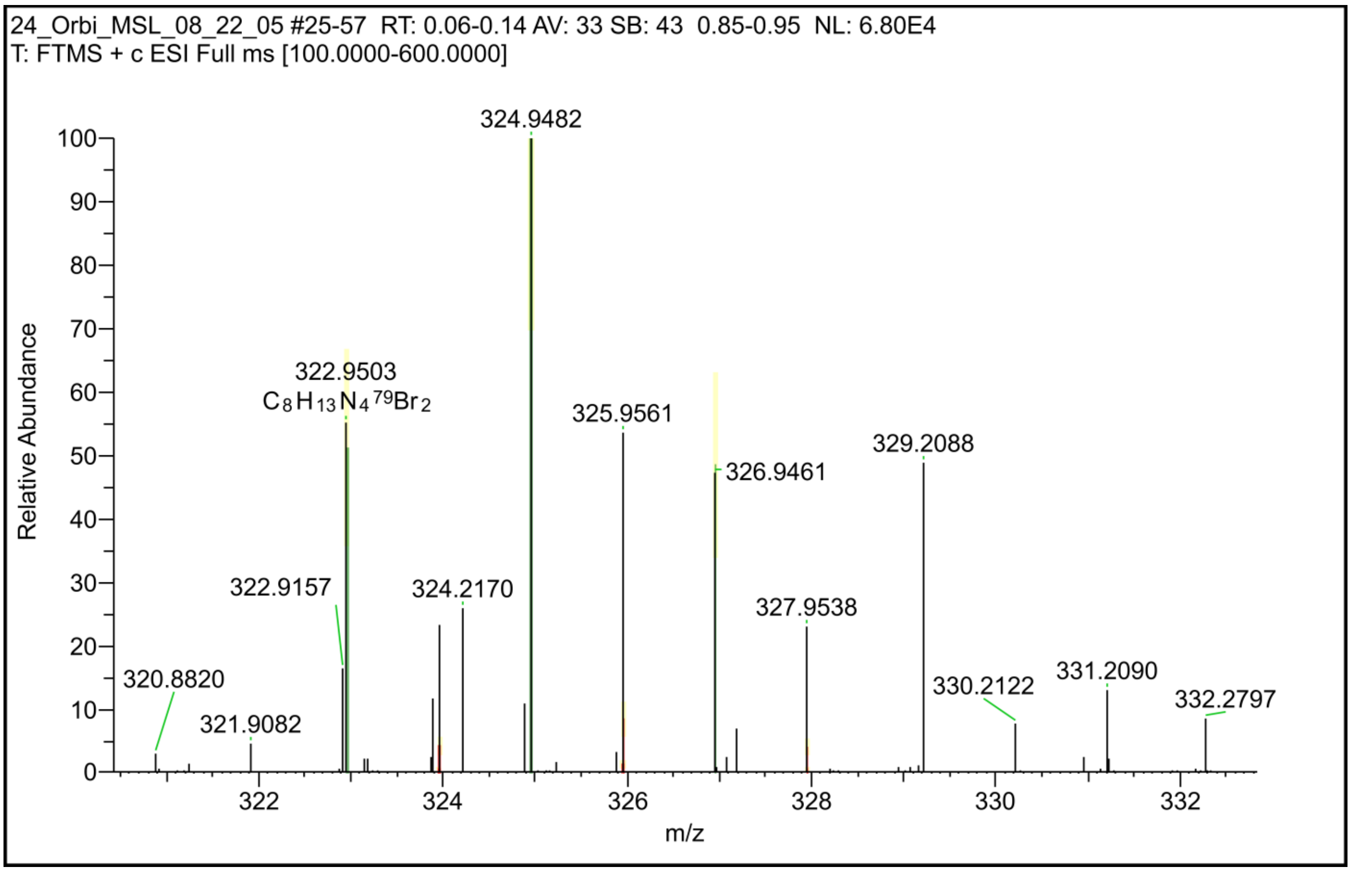

Figure S52: ESI-HRMS of compound **5b**.

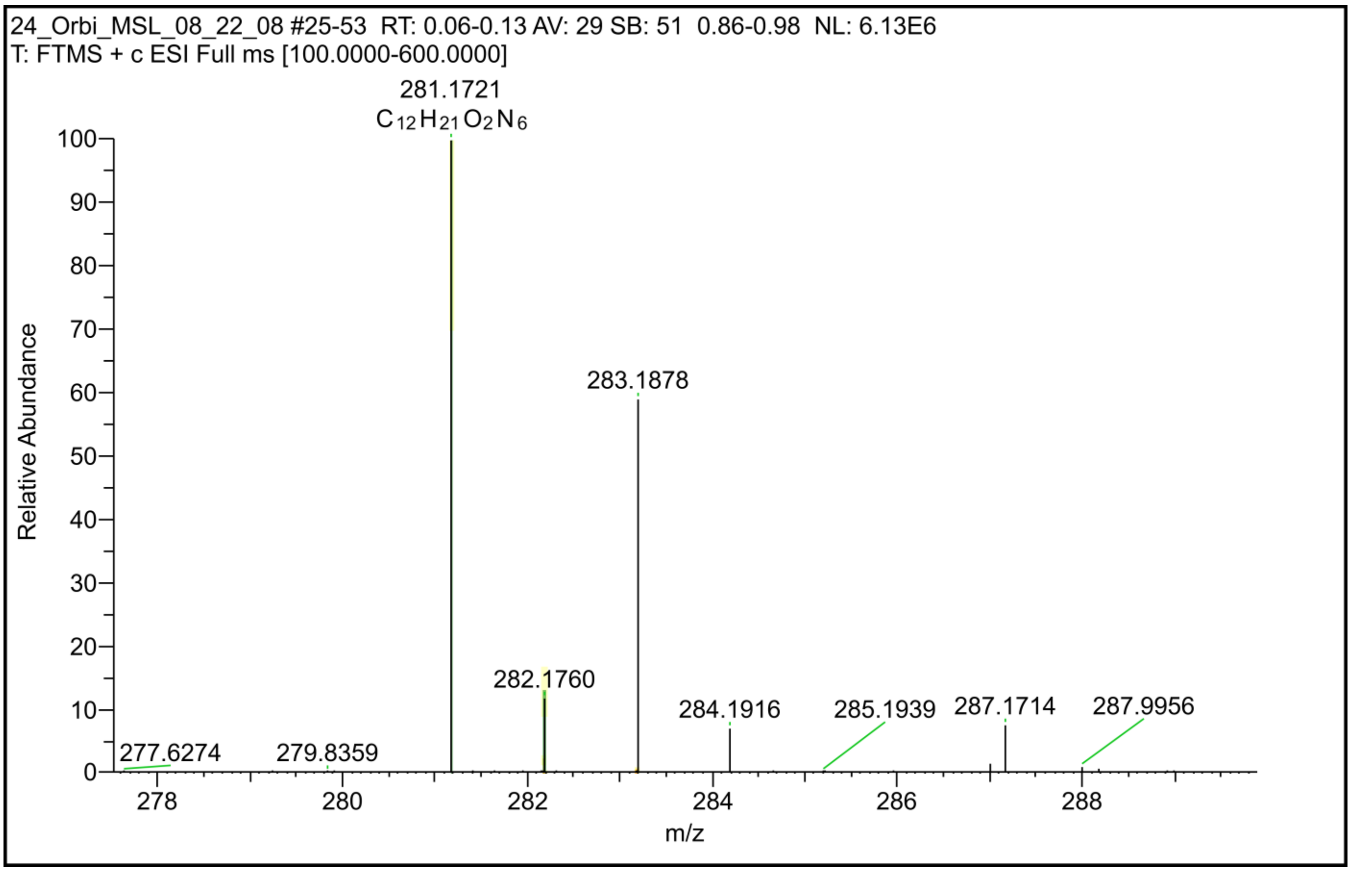

Figure S53: ESI-HRMS of compound **7**.

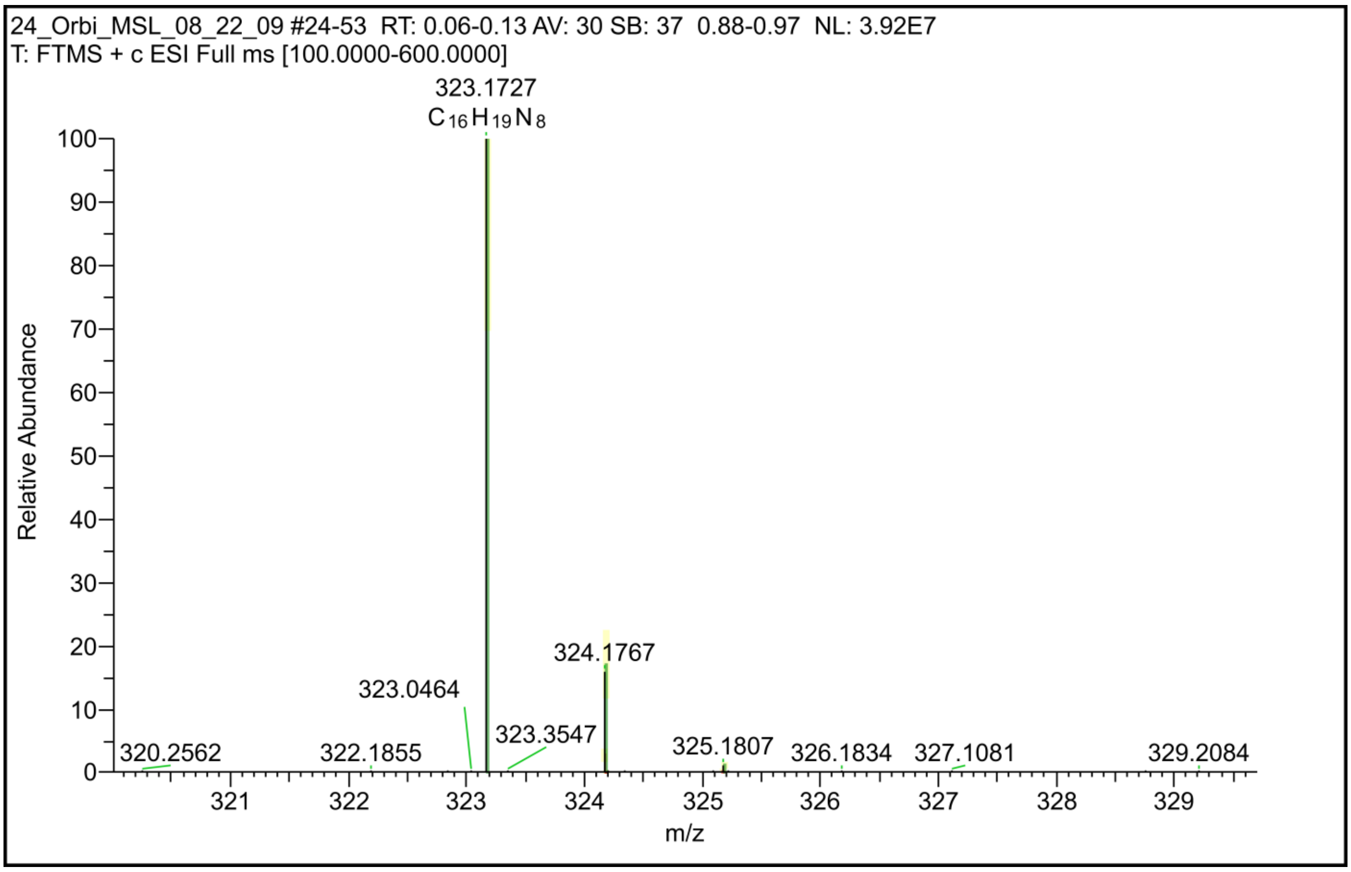

Figure S54: ESI-HRMS of compound **9**.

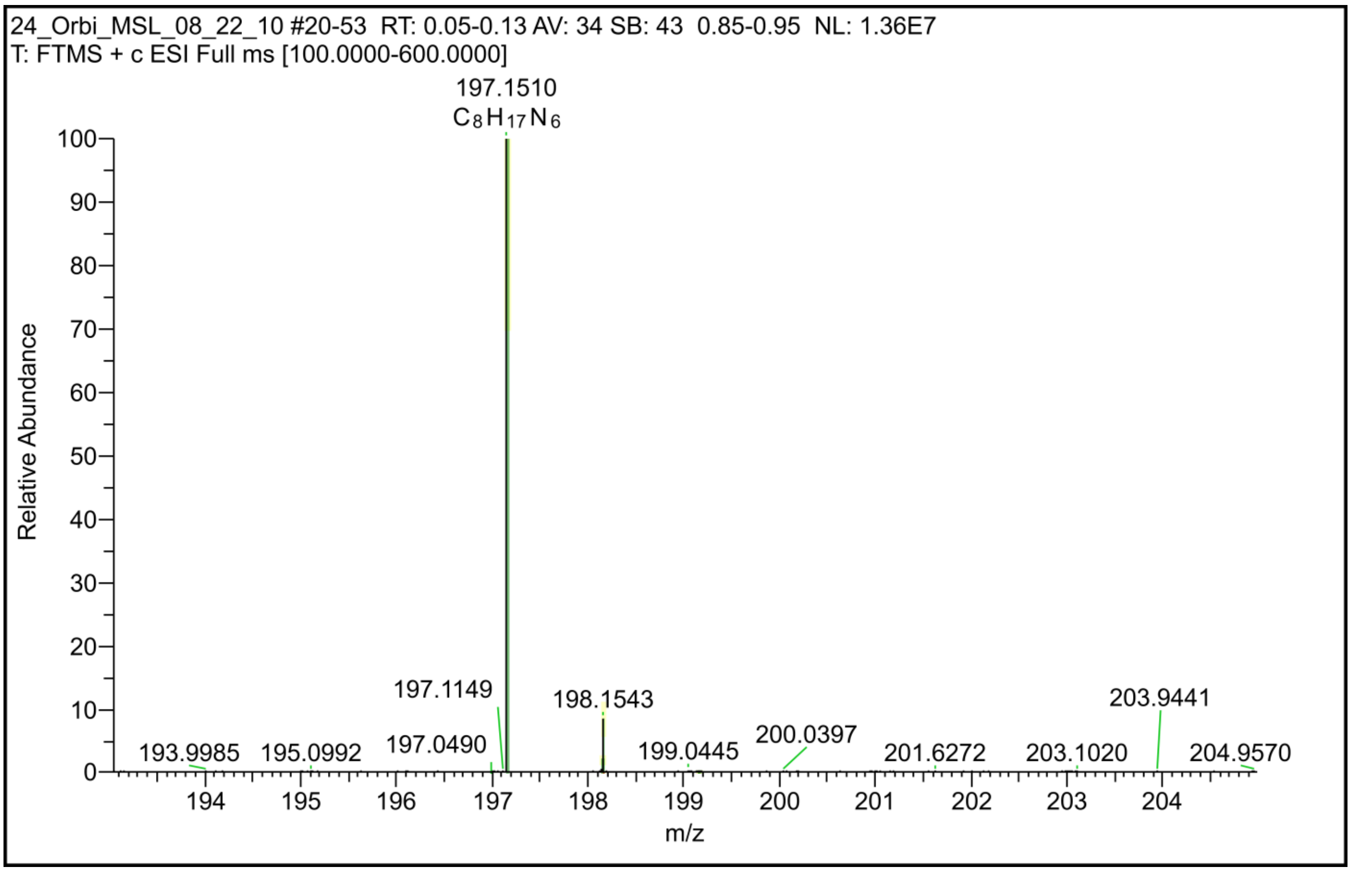

Figure S55: ESI-HRMS of compound **10**.

Cartesian co-ordinates of TS for theoretical calculations

For compound 4a:

C	-0.865256000000	-1.000471000000	-0.191363000000
N	-0.134752000000	-1.399257000000	-1.307409000000
N	1.124912000000	-1.146714000000	-1.186435000000
C	1.535804000000	-0.686501000000	0.030456000000
N	0.917209000000	-1.204607000000	1.132989000000
N	-0.340154000000	-1.460794000000	1.014988000000
C	-2.363476000000	-1.051587000000	-0.350346000000
C	2.902653000000	-0.109989000000	0.166752000000
H	-2.669292000000	-0.553342000000	-1.269588000000
H	-2.695339000000	-2.090625000000	-0.356234000000
H	3.370636000000	-0.436969000000	1.094508000000
H	3.517617000000	-0.363649000000	-0.695720000000
F	2.822638000000	1.304305000000	0.218887000000
F	-2.975455000000	-0.407019000000	0.728873000000
C	-0.382513000000	0.824713000000	-0.102855000000
N	-0.758452000000	1.917525000000	-0.126996000000
C	-1.163120000000	3.284265000000	-0.143858000000
H	-1.578547000000	3.514060000000	-1.124237000000
H	-1.919246000000	3.434953000000	0.625746000000
H	-0.296375000000	3.914335000000	0.053018000000

For compound 4b:

C	1.122410000	-0.639592000	0.007987000
N	0.555538000	-1.137907000	1.179397000
N	-0.728341000	-1.071497000	1.176238000
C	-1.281947000	-0.614367000	0.002138000
N	-0.724129000	-1.121381000	-1.147063000
N	0.560247000	-1.179385000	-1.147717000
C	2.628356000	-0.427400000	0.002131000
C	-2.745828000	-0.230343000	-0.006580000
F	-2.920760000	0.581964000	-1.171107000
F	2.899021000	0.413102000	1.124979000
C	3.362381000	-1.739317000	0.234379000
H	4.437239000	-1.553891000	0.273792000
H	3.035829000	-2.196068000	1.168481000
H	3.154152000	-2.427334000	-0.586849000
C	-3.644305000	-1.447333000	-0.173683000
H	-3.381733000	-1.989213000	-1.082786000
H	-3.521124000	-2.114638000	0.681508000
H	-4.688082000	-1.132042000	-0.225891000
C	-3.111047000	0.623338000	1.193942000
H	-2.960981000	0.055700000	2.113138000
H	-2.489896000	1.519399000	1.232649000
H	-4.159669000	0.916766000	1.127795000
C	3.102011000	0.303914000	-1.239789000
H	2.884165000	-0.293354000	-2.125877000
H	2.607656000	1.270527000	-1.340066000
H	4.179588000	0.461786000	-1.178351000
C	0.174395000	1.038080000	-0.015322000
N	0.296501000	2.190754000	-0.012014000

C	0.405457000	3.611110000	-0.002351000
H	0.975417000	3.914160000	0.875433000
H	0.924461000	3.929756000	-0.905943000
H	-0.591374000	4.050224000	0.029127000

For compound 5a:

C	-1.157370000000	0.180617000000	0.000044000000
N	-0.588676000000	-0.336374000000	-1.170403000000
N	0.691989000000	-0.251831000000	-1.167077000000
C	1.252796000000	0.209171000000	0.000072000000
N	0.691959000000	-0.251736000000	1.167246000000
N	-0.588705000000	-0.336280000000	1.170547000000
C	-2.638210000000	0.406094000000	0.000023000000
C	2.667996000000	0.642020000000	0.000085000000
H	-2.952709000000	0.933386000000	0.895221000000
H	-2.952686000000	0.933429000000	-0.895158000000
H	2.912613000000	1.202730000000	-0.895735000000
H	2.912626000000	1.202592000000	0.895989000000
Br	3.932327000000	-0.911786000000	-0.000045000000
Br	-3.628462000000	-1.304552000000	-0.000031000000
C	-0.341393000000	1.860120000000	-0.000017000000
N	-0.552778000000	2.998879000000	-0.000053000000
C	-0.742662000000	4.411720000000	-0.000078000000
H	-1.303949000000	4.688936000000	-0.891710000000
H	-1.303657000000	4.689002000000	0.891718000000
H	0.229028000000	4.904704000000	-0.000254000000

For compound 5b:

C	-1.040175000	-0.741472000	0.348398000
N	-0.497665000	-1.068601000	-0.894157000
N	0.720345000	-0.685676000	-1.023196000
C	1.290409000	-0.133683000	0.098872000
N	0.989010000	-0.751560000	1.283769000
N	-0.230872000	-1.137645000	1.415518000
C	-2.530123000	-0.960079000	0.551959000
C	2.559648000	0.656506000	-0.027285000
C	-2.867657000	-2.441091000	0.402744000
H	-2.348318000	-2.993601000	1.190554000
H	-3.940977000	-2.595660000	0.516468000
H	-2.545621000	-2.824760000	-0.564057000
C	2.570657000	1.521913000	-1.277750000
H	3.526618000	2.036642000	-1.371347000
H	2.385933000	0.934178000	-2.175070000
H	1.781037000	2.274368000	-1.188001000
C	2.868834000	1.445585000	1.235272000
H	2.083347000	2.193908000	1.379055000
H	2.898125000	0.803790000	2.113899000
H	3.822272000	1.963172000	1.132052000
C	-3.018135000	-0.389701000	1.875070000
H	-2.507044000	-0.908989000	2.689100000
H	-2.813631000	0.676706000	1.961456000
H	-4.090981000	-0.551283000	1.977320000
Br	-3.485288000	0.031483000	-0.933354000
Br	4.080557000	-0.718412000	-0.249955000
C	-0.590377000	1.123182000	0.348992000
N	-1.010852000	2.200566000	0.408369000

C	-1.547486000	3.518879000	0.449824000
H	-0.854442000	4.204710000	-0.036295000
H	-2.506311000	3.521801000	-0.068863000
H	-1.687753000	3.807713000	1.490878000

For compound methyl Isocyanide:

C	1.481075000	-0.000027000	-0.000016000
N	0.315252000	0.000041000	0.000031000
C	-1.112270000	0.000001000	-0.000023000
H	-1.473216000	0.993432000	-0.265163000
H	-1.473151000	-0.726445000	-0.727716000
H	-1.473229000	-0.267122000	0.992896000
